# Aldehydes as CO Releasing Molecules: *In Situ* and *Ex Situ* Giese Reactions and Palladium-Catalyzed Aminocarbonylations

**DOI:** 10.1021/acs.orglett.5c03618

**Published:** 2025-09-25

**Authors:** Elena Cassera, Maurizio Fagnoni

**Affiliations:** PhotoGreen Lab, Department of Chemistry, 19001 University of Pavia, Viale Taramelli 12, 27100 Pavia, Italy

## Abstract

We report the adoption of a two-chamber reactor making use of aliphatic aldehydes in the role of CO releasing molecules (CORMs). Upon photocatalytic conditions, an acyl radical (RCO^•^) was first formed, prone to lose CO. The resulting alkyl radical was employed in the *in situ* conjugate addition onto a Michael acceptor (in the first chamber), and the released CO was employed in *ex situ* palladium-catalyzed aminocarbonylations (in the second chamber) to ensure a 100% atom-economical reaction.

## Graphical abstract

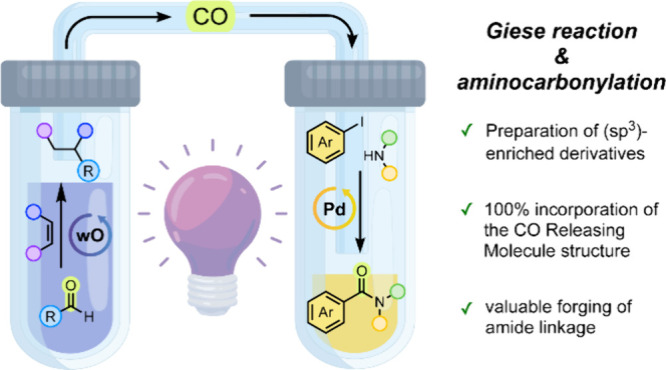

Carbon monoxide is deemed as a tasteless, colorless, odorless, very toxic gas that can induce illness and death if inhaled. Nevertheless, CO could act as a potential therapeutic agent exerting vasorelaxation or showing anti-inflammatory properties.^1^ Such a gas has important implications in synthesis as one of the most important C1 synthons (Scheme 1a).^2^ Due to the hazard in handling CO, scientists adopted its *in situ* release starting from safer and stable compounds dubbed as CORMs (CO Releasing Molecules).^3^ This decarbonylation may be an appealing route to induce defunctionalization in organic molecules,^4^ and the controlled release of CO is a hot topic in biology for therapeutic applications.^5^ Some CO surrogates have been introduced mostly activated by heating,^6^ but the CO photorelease is an option.^7^ The CORM is usually responsible for *in situ* generation of CO to be used in the same flask. The *ex situ* variant is gaining importance^8^ by adopting a two-chamber reactor where in one chamber (Chamber A) the CO is produced and diffused into an adjacent chamber (Chamber B) where a purposely designed carbonylation took place (Scheme 1b).^8^ A drawback of these procedures is that the remaining part of the CORM (after CO loss) is wasted, thus impacting the atom economy of the reaction. We then wondered if it was possible to design a two-chamber reactor where a purposely devised CORM will liberate CO that will be engaged in a typical carbonylation reaction, but the remaining fragment of the CORM will be used in another fruitful reaction in Chamber A. Recently, we tested some aldehydes where the formyl group, dubbed as “HAcTive group”, had the role to direct the photocatalyzed hydrogen atom abstraction in the substrate.^9^ In such a way, an acyl radical that resulted upon CO loss easily gave access to various substituted carbon-based radicals (e.g., *tert*-butyl, benzyl, α-oxy etc.).^9,10^

**Scheme 1:**
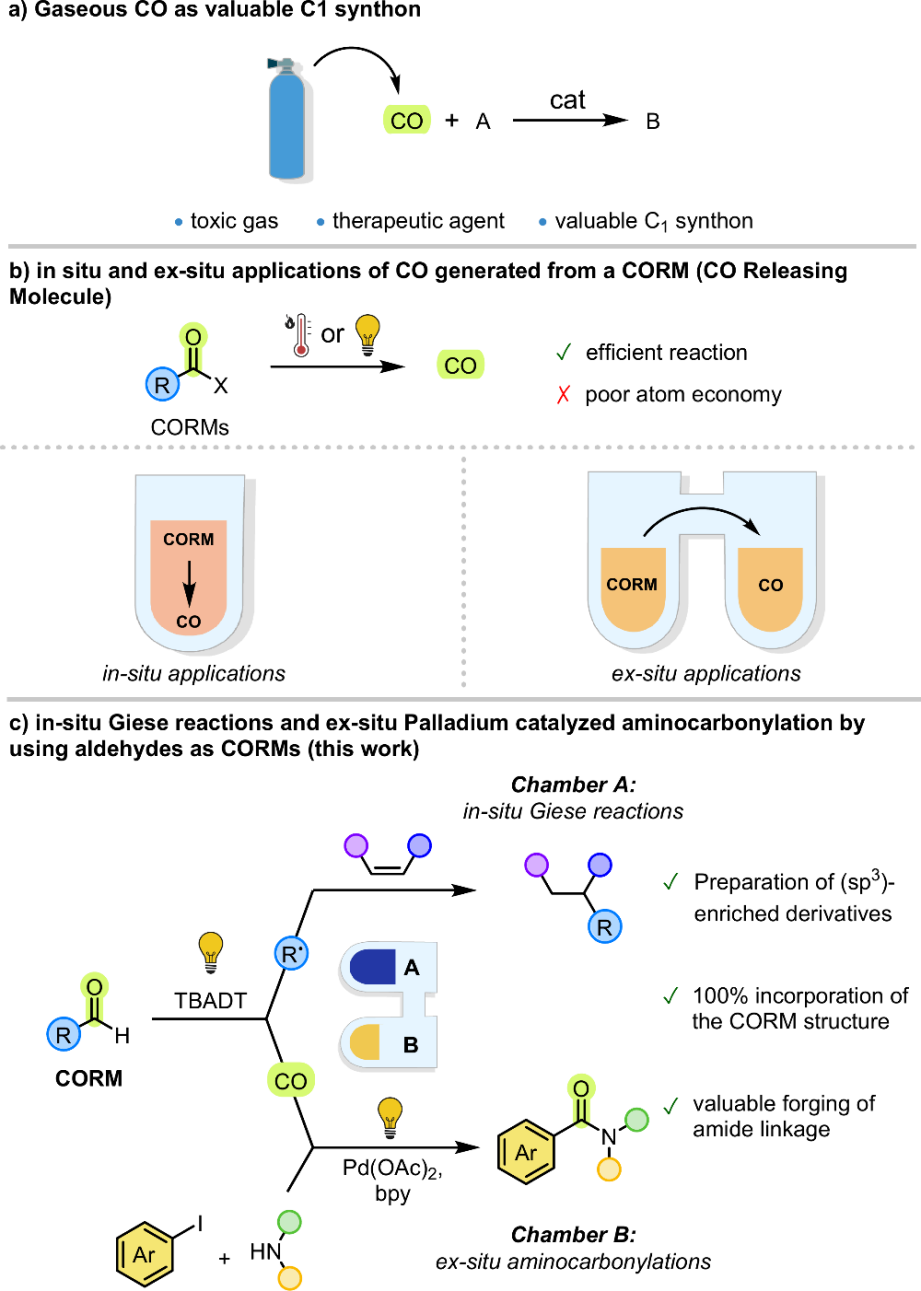
Aldehydes as CO Releasing Molecules (CORMs)

We then envisaged a two-chamber apparatus where in Chamber A an aldehyde underwent a HAT process upon light irradiation in the presence of the tetrabutylammonium salt of the decatungstate anion (TBADT). The carbon radical released in Chamber A then engaged in a Giese reaction to forge a C–C bond via conjugate addition onto Michael acceptors. Meanwhile, the CO photogenerated is free to reach Chamber B and is used in palladium-catalyzed aminocarbonylations. As a result, both *in situ* and *ex situ* reactions were performed in two distinct chambers assuring a 100% product incorporation of the CORM structure (Scheme 1c). We reasoned that, in Chamber A, the radicals liberated could be those having three-dimensional aliphatic (ring) fragments to increase both the lipophilicity of the resulting derivatives and the fractional sp^3^ character (Fsp^3^), a must in drug design strategy.^11^ To this aim, the introduction of quaternary carbons^12^ eventually containing a saturated (rather than aromatic^13^) ring was then planned. In Chamber B, for the valorization of CO as a C1 synthon, we explored aminocarbonylations starting from aryl iodides and amines to forge the amide bonds (−CONR_2_) due to their wide occurrence (Scheme 1c).^14–17^ Despite the supposed low reactivity of amides, they may be easily converted into other functional groups, e.g., amines.^18^ We started our investigation from pivaldehyde **1a** due to the importance of installing a *tert-*butyl group in an organic molecule.^19,20^ The reaction conditions of the preparation of the Giese adducts were chosen from literature precedents.^9,21^

The aminocarbonylations took place under photoinduced Pd-catalyzed conditions.^22^ The reaction was carried out with the idea to use the same 390 nm lamp employed for the generation of CO in Chamber A (see Figure S2, Supporting Information).^22c^ In Chamber B, aryl iodides (**3a**–**l**) were chosen as halides, and for amines (**4a**–**4j**), we preferentially selected saturated nitrogen heterocycles (e.g., the piperidine ring^23^) due to their occurrence in pharmaceuticals.

To test the feasibility of the system, we assembled the two-chamber photoreactor depicted in Figure 1 (and Figure S1, Supporting Information) to optimize the aminocarbonylation conditions. In Chamber A, it was planned to liberate two equiv of CO with respect to the amount of the aryl iodide in Chamber B. We then fixed the alkylation of dimethyl maleate (**2a**, 0.6 mmol) with pivaldehyde (**1a**, 1.5 equiv) in Chamber A, and we investigated the coupling between 4-iodoanisole (**3a**, 0.3 mmol) and pyrrolidine (**4a**, 3 equiv) chosen as a model reaction in Chamber B (Table 1, see Table S1 for further experiments). In Chamber A, the *tert-*butylation of the Michael acceptor smoothly occurred and led to succinate **5A** with a GC yield in the range of 92–98%. In Chamber B, the reaction carried out in 2-MeTHF/water 7:3 adopting Pd­(PPh_3_)_4_ (6 mol %) as the catalyst^24^ in the presence of a base (K_2_CO_3_, 0.3 mmol) led to an encouraging formation of amide **5B** (81% yield, entry 1).

**Figure 1:**
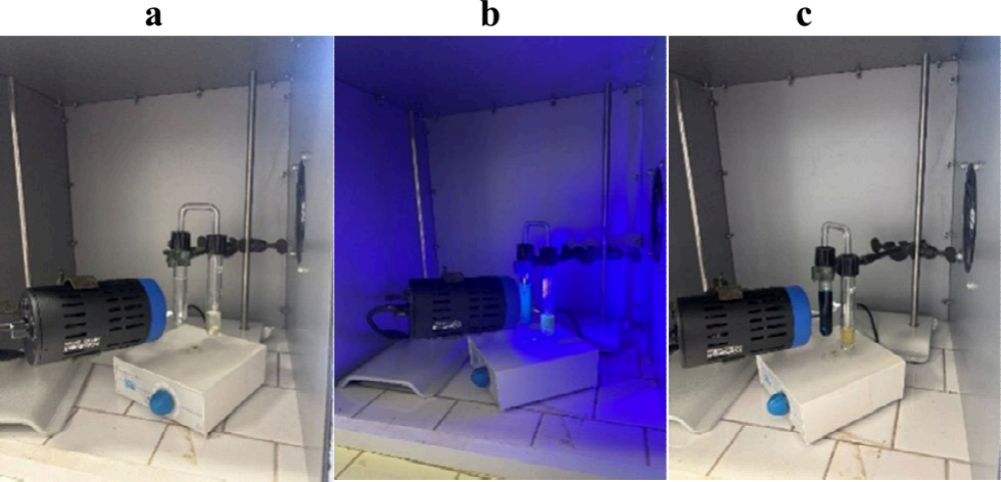
Setup employed for the *in situ* Giese reactions and *ex situ* palladium catalyzed aminocarbonylations **a.** before irradiation, **b.** during irradiation, and **c.** after irradiation.

**Table 1:**
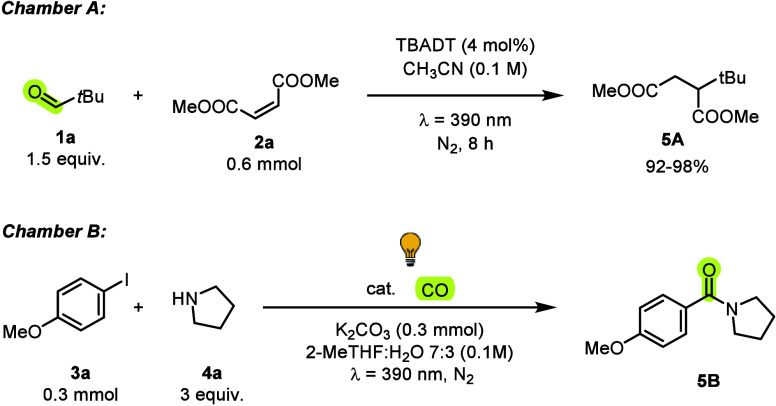
Selected Optimization Experiments

Entry	Catalyst (6 mol %)	Additive(s)	λ (nm)	Yield^a^
1	Pd(PPh_3_)_4_	/	390	81%
2	Pd(OAc)_2_	/	390	62%
**3**	**Pd(OAc)**_**2**_	**bpy (8 mol %)**	**390**	**84% (78%)**
4	Pd(OAc)_2_	bpy (8 mol %)	^b^	28%
5	/	/	390	nr
6	Pd(OAc)_2_	bpy (8 mol %), TEMPO (4 equiv)	390	15%

a GC yields determined by using 1,3,5 trimethoxybenzene as the external STD (in parentheses isolated yield).

b Chamber B was covered with an aluminum foil.

The main drawback of the process was the tedious isolation of **5B** by column chromatography due to the presence of Ph_3_PO as the byproduct. We then tested Pd­(OAc)_2_, but an unsatisfying yield (62%, entry 2) resulted. The addition of bpy (8 mol %) markedly improved the efficiency of the process (78% isolated yield, entry 3). Control experiments confirm that photolysis (entry 4) and the catalytic system (entry 5) are mandatory for efficient occurrence of amide preparation. The presence of TEMPO (entry 6) or oxygen (Table S1) or the adoption of alternative solvents (Table S1) dramatically reduced the formation of **5B**. We then used this photochemical system to allow the formation of two distinct products (Giese adducts, compounds **A**, and amides, compounds **B**) in one single step in the two-chamber reactor. The reactions were coupled to form new products in each experiment, as depicted in Scheme 2. We initially maintained pivaldehyde as the CORM (by changing the SOMOphile in Chamber A) and pyrrolidine as the secondary amine (by changing the aryl iodide in Chamber B). In Chamber A, the Giese adducts were consistently formed in more than 70% yield by adopting different unsaturated ketones, nitriles, esters amides, etc., including 2-vinylpyridine (compounds **5A**–**13A**). In Chamber B, various aryl­(pyrrolidin-1-yl)­methanones **5B**–**13B** were obtained in up to 78% yield by varying substituted phenyl iodides having either electron-donating (Me, OMe) or electron-withdrawing (CN, COMe, NO_2_ etc) groups. The synthesis of **5A**/**5B** was repeated on a 1 mmol scale by using a larger two-chamber reactor (see Figure S5). As a result, the overall yields were only slightly affected by the scale-up of the reaction (Scheme 2). When haloiodoaromatics (**3d**–**3f**) were used as arylating agents, only the iodine atom was removed in the carbonylation leaving untouched the other halogens (see compounds **8B**–**10B**). Carbonylation yields were likewise in the 50–75% range when using 1-iodonaphthalene (amide **14B**) or iodoheteroaromatics (amides **15B**-**16B**). With the aim to introduce lipophilic moieties in complex molecules, we prepared the acrylate ester of selected chiral alcohols such as menthol (**2j**), cedrol (**2k**), paracetamol (**2l**), cholesterol (**2m**), and Vitamin E (**2n**, Scheme 2). These esters were photocatalytically reacted with **1a** to form the corresponding Giese adducts (**14A**–**18A**) in a satisfying yield. The presence in solution of an excellent hydrogen donor such as an aldehyde prevented any undesired C–H activation (or racemization) induced by the photocatalyst on the starting acrylate,^25^ thus highlighting the exceptional features of this approach for selective late-stage functionalization.^9,26^

**Scheme 2:**
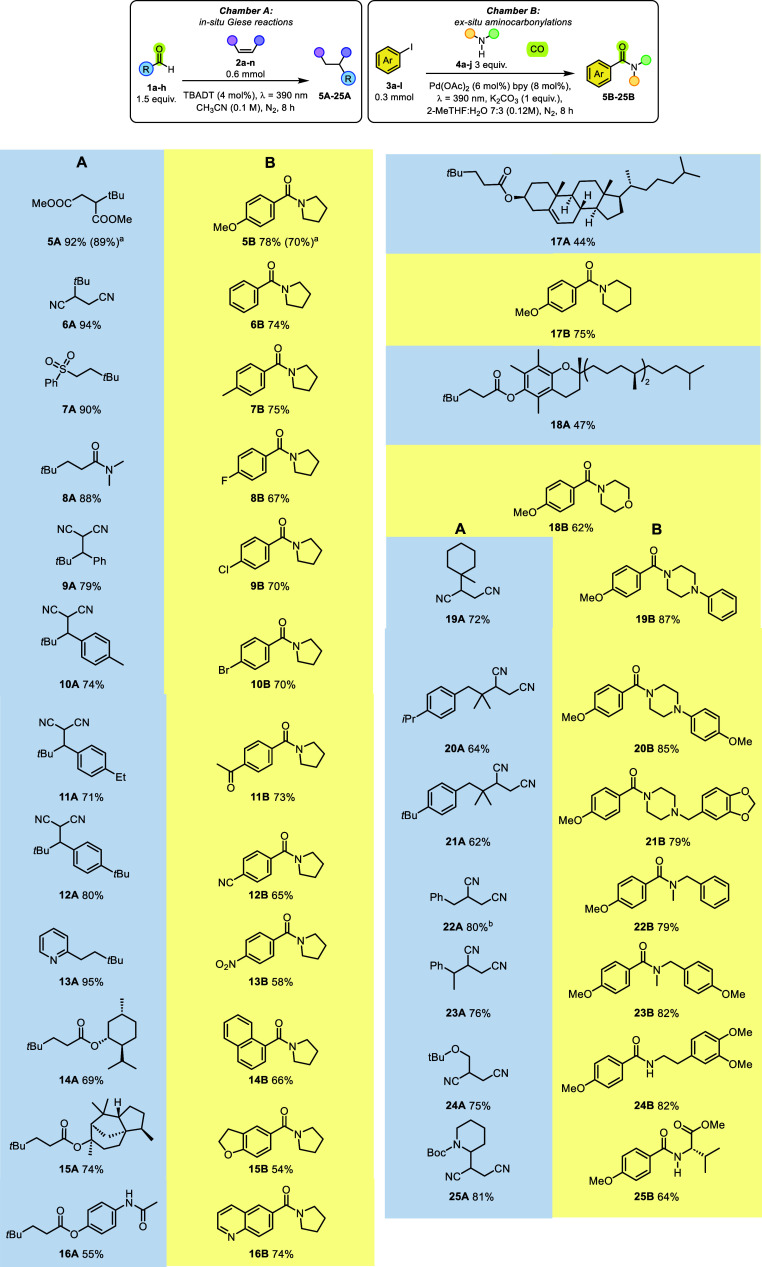
Formation of Giese Adducts (Compounds **A**) and an Amide (Compounds **B**) in One Single Step

a Yields referred to 1 mmol scale reaction.
b A mixture of MeCN:DCM 11:1 was employed as the solvent.

We then made a screening of other possible CORMs, and we used the resulting carbon radicals for the alkylation of fumarodinitrile **2b** in Chamber A, and, at the same time, we tested different amines by maintaining iodoaromatic **3a** in Chamber B. We investigated other tertiary aldehydes namely 1-methylcyclohexane-1-carbaldehyde (**1b**) and 3-(4-alkylphenyl)-2,2-dimethylpropanals (**1c**, **1d**). In both cases, the selective formyl hydrogen activation took place avoiding competition with the labile benzyl hydrogens in **1c,1d** with the exclusive formation of a tertiary alkyl radical (via CO loss) as witnessed by the isolation of compounds **19A**–**21A**. Phenylacetaldehydes **1e, 1f** were suitable substrates for the generation of (substituted) benzyl radicals to give **22A**–**23A**. The last case concerns the generation of α-to-heteroatom radicals from 2-(*tert*-butoxy)­acetaldehyde **1g** and *N*-boc-2-piperidinecarbaldehyde **1h**. As expected,^9^ decarbonylation was effective, and *tert*-butyl ether **24A** and *N*-Boc-protected derivative **25A** were isolated in 75% and 81% yield, respectively (Scheme 2). In Chamber B, the formation of amides was not affected by the choice of amine (whether cyclic, acyclic or even primary). Thus, various monocyclic nitrogen-containing six-membered rings, namely piperidine **4b**, morpholine **4c**, and *N*-substituted piperazines **4d**–**4f** were first tested, and amides **19B**–**21B** were exclusively formed. Shifting to secondary benzyl amines **4g** and **4h** assured a good yield of the corresponding amides (**22B**–**23B**). As a representative case of a primary amine, the biologically active 3,4-dimethoxyphenethylamine (DMPEA, **4i**) was easily converted into **24B** under the optimized conditions. Noteworthily, a C-protected amino acid ((*S*)-valine methyl ester hydrochloride **4j**) likewise gave benzamide **25B** in a satisfying yield. The protocol described here has several advantages. First, in a single run, the forging of a C­(sp^3^)–C­(sp^3^) bond (in Chamber A) and a C­(sp^2^)–C­(sp^2^)/C­(sp^2^)–N bond (in Chamber B) took place. This allows, on one hand, the hydroalkylation of (biobased derived) olefins by the incorporation of aliphatic groups to increase the Fsp^3^ value,^11,12^ which is a strategy for late-stage functionalization.^26^ On the other hand, the concomitant preparation of various benzamides mainly incorporating nitrogen-based heterocycles was formed. Cyclic and acyclic secondary amines as well as primary amines (or protected amino acids) are effective for their conversion in amides.

The present protocol is safe since it makes use of 1 atm of CO released at room temperature under very mild conditions without the need to handle CO cylinders or adopt stainless-steel autoclaves (or sealed reactors) to ensure high CO pressure. The released CO first saturates the liquid phase in Chamber A and then diffuses through the headspace into Chamber B. Dissolution of CO in the reaction medium completes these multiple transfer steps. The greenness of the reaction is witnessed by the incorporation of all the atoms of the CORM in the end products, resulting in a 100% atom-economical reaction. A bonus is the choice of the same light source same (390 nm LEDs) to promote both processes in each Chamber (Table 1).

On the basis of the experiments carried out, we propose a tentative mechanism of the reaction as depicted in Scheme 3. In Chamber A, the activation of the aldehyde was carried out by hydrogen atom abstraction^27^ photocatalyzed by the decatungstate anion^28^ upon UV LED irradiation. This polyoxometalate is the elective photocatalyst for the generation of acyl radicals from aldehydes.^27a,29^ As recently demonstrated, the formyl group may act as a suitable Traceless Activating Group (TAG) that reacts faster than other aliphatic C–H bonds (even more labile) present in the substrates.^9^ The aldehydes **1a**–**h** have been chosen for the lack of stability of the resulting acyl radical **I** that is prone to release CO and the corresponding alkyl radical **II** from it. The *in situ*-generated carbon-based radical is then trapped by a SOMOphile (an electron-poor olefin or a vinyl arene), and the resulting radical adduct **III** led to the Giese products **5A–25A** (by back hydrogen transfer, *b*HAT) causing the concomitant regeneration of the photocatalyst. The forging of a C­(sp^3^)–C­(sp^3^) bond resulted, and the alkyl groups incorporated in the olefin were mainly chosen to increase the Fsp^3^ value of the Giese adducts.^11^ We then proposed a plausible reaction mechanism occurring in Chamber B based on literature precedents.^30^ The active species is the Pd(0)­bpy complex *in situ* obtained by the reduction of the Pd­(II) precatalyst (Pd­(OAc)_2_) possibly by an interaction between the metal center and a suitable redox partner, e.g., amines **4a**–**4j** (See Figure S3b).^31^ Upon light irradiation, the excited Pd(0) catalyst thus formed initiates a radical-based mechanism, coordinating aryl iodides **3a**–**3l**, to give the Pd­(I)–radical species **IV**, after an inner-sphere single electron transfer (iSET).^30^ Figure S3a evidenced that the catalytic system absorbs at 390 nm, and on–off experiments (Figure S4) demonstrated that the reaction is markedly accelerated upon light exposure. Incorporation of carbon monoxide (coming from Chamber A) by **IV** results in the formation of the acyl intermediate **V**, which first coordinates amines **4** and then evolves in Pd­(II) complex **VI**, with the concomitant release of HI (neutralized by the basic environment).

**Scheme 3:**
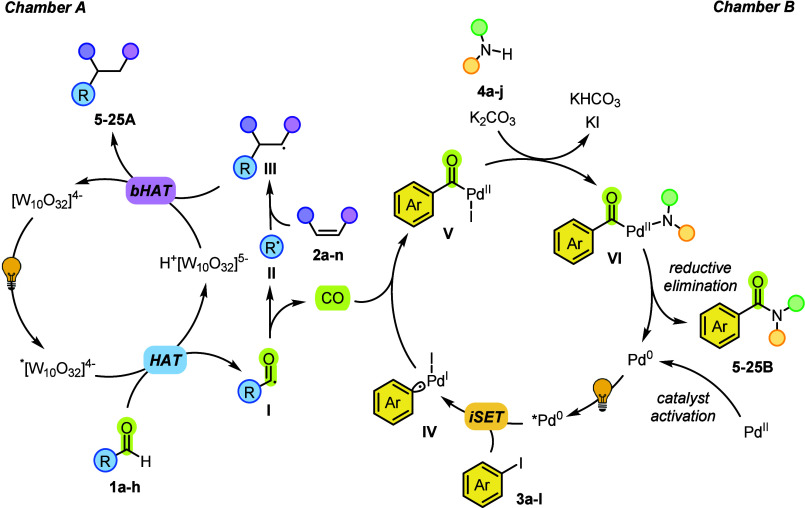
Proposed Mechanism

Finally, amides **5B–25B** are obtained after reductive elimination with the concomitant regeneration of the active catalytic species Pd(0)­bpy. The radical nature of the process occurring in Chamber B was supported by the efficient quenching of TEMPO on the reaction (Table 1). The apparatus designed allows for an efficient interchamber CO transfer leading to an impressive incorporation of the entire structure of the CORM in the functionalized olefins and in the amides.

In summary, we have developed a practical and sustainable protocol for the concomitant alkylation of electron-poor olefins along with the preparation of benzamides via carbonylation of aryl iodides in the presence of amines. The approach is based on the easy release of CO from widely available aldehydes in the role of CORMs upon decatungstate photocatalyzed hydrogen abstraction, followed by fragmentation of the resulting acyl radical. This is another valuable example of a biphasic gas–liquid reaction where a photoliberated low-molecular weight gas is used in synthetic planning.^32^

## Supplementary Material



## Data Availability

The data underlying this study are available in the published article and its Supporting Information and openly available in ChemRxiv at 10.26434/chemrxiv-2025-2s05k.

## Notes

Experimental procedures, characterizing data, details of additional experiments, NMR spectra (PDF)
