# Supplementary material for: Aldehydes as CO Releasing Molecules: In Situ and Ex Situ Giese Reactions and Palladium-Catalyzed Aminocarbonylations
Source: Org Lett. 2025 Sep 25;27(43):11999–2005. doi: 10.1021/acs.orglett.5c03618 (PMC12584138; doi:10.1021/acs.orglett.5c03618)

Supporting Information

Aldehydes as CO Releasing Molecules: *in-situ* and *ex-situ* Giese Reactions and Palladium Catalyzed Aminocarbonylations

*Elena Cassera, Maurizio Fagnoni**

PhotoGreen Lab, Department of Chemistry, University of Pavia, Viale Taramelli 12, 27100 Pavia, Italy. E-mail: maurizio.fagnoni@unipv.it

CONTENT:

1 Experimental Details p. S3

1.1 General information p. S3

1.2 Experimental setup p. S5

1.3 Chart of the starting materials p. S6

1.4 Absorption spectra of the Pd catalyst system p. S7

1.5 Optimization of the reaction conditions p. S8

1.6 Light On-Off experiments p. S9

1.7 Experimental procedures p. S10

1.8 Characterization data for products **5A-25A** p. S16

1.9 Characterization data for products **5B-25B** p. S22

2 References p. S30

3 NMR Spectra p. S32

1. Experimental Details

1.1 General information

Chemicals. All reagents (aldehydes **1a-h**, electron poor olefins **2a-n**, iodides **3a-l** amines **4a-j**) and chemicals used in this work were commercially available and were purchased from various commercial suppliers (TCI Europe, Sigma Aldrich, Fluorochem), except where otherwise noted. The solvents employed in this work were purchased from Carlo Erba or Sigma Aldrich and used as received, except where otherwise noted. Column chromatography was performed on an Isolera Spektra One purchased from Biotage, Sweden, using Sepachrom PUREZZA open-load flash cartridges purchased from Sepachrom Srl, Italy. n-hexane (Hex), ethyl acetate (EtOAc), dichloromethane (DCM) and methanol (MeOH) were used as eluants.

NMR spectroscopy. ^{1}H and ^{13}C NMR spectra were recorded at 300 K on a 300 MHz Bruker spectrometer and on a 400 MHz Bruker spectrometer, in $CDCl_3$, using the solvent residual signals as internal reference (7.26 and 77.0 ppm for $CDCl_3$, respectively for ^{1}H and ^{13}C). Multiplicity is reported as follows: s = singlet, bs = broad singlet, d = doublet, t = triplet, q = quadruplet, quint = quintuplet, hept = heptet, dd = doublet of doublets, td = triplet of doublets, pd = doublet of quintets, m = multiplet. Chemical shifts (δ) and coupling constants (*J*) are given in ppm and in Hz, respectively. NMR data were processed using the MestReNova 14 software package. Known products were characterized by comparing to the corresponding ^{1}H NMR, ^{13}C NMR with those available in the literature.

Melting point. Melting points were measured with an Electrothermal apparatus and are uncorrected.

UV–Vis spectroscopy. UV-Vis absorption spectra were recorded on a V-550 Jasco spectrophotometer.

Mass spectrometry. HRMS data were acquired using a X500B QTOF System (SCIEX, Framingham, MA 01701 USA), equipped with the Twin Sprayer ESI probe and coupled to an ExionLC™ system (SCIEX). The SCIEX OS software 2.1.6 was used as an operating platform. For MS detection the following parameters were applied: Curtain gas 30 psi, Ion source gas 1 45

psi, Ion source gas 2 55 psi, Temperature 450 °C, Polarity positive, Ion spray voltage -4500 V, TOF mass range 50-1600 Da, declustering potential -60 V and collision energy -10 V.

Reaction monitoring and compound quantification GC-FID analyses were performed on an HP 5890 series II chromatograph and on an Agilent 7820A chromatograph. In both cases, the injection was performed at 250°C in split mode. The initial oven temperature of 80 °C was maintained for 2 min, increased by 10 °C/min to 250 °C and held for 5 min. An Agilent HP5 30 m × 0.32 mm × 0.25 µm film thickness capillary column was used with nitrogen as the carrier gas at a constant flow rate of 6.0 mL·min $^{-1}$.

GC-MS analyses were carried out using a Thermo Scientific DSQII single quadrupole GC-MS system. A Restek Rtx-5MS (30 m x 0.25 mm x 0.25 µm) capillary column was used for analytes separation with helium as carrier gas at 1mL·min $^{-1}$. The injection in the GC system was performed in split mode and the injector temperature was 250 °C. The GC oven temperature was held at 80 °C for 2 min, increased to 220 °C by a temperature ramp of 10 °C·min $^{-1}$ and held for 10 min. The transfer line temperature was 250 °C and the ion source temperature was 250 °C. Mass spectral analyses were carried out in full scan mode. EI ionization potential was 70 keV. After irradiation, the yield of the products was determined by GC-MS analyses through calibration curves obtained with authentic samples of the relevant compounds, using 1,3,5-trimethoxybenzene or undecane as the internal standard.

1.2 Experimental setup

The photoreactor consists of (16x100 mm pyrex tubes, equipped with stirring bars), closed with SVL hole screw caps (with sealing rings), linked with a glass bridge (diameter 4 mm, Figure S1).

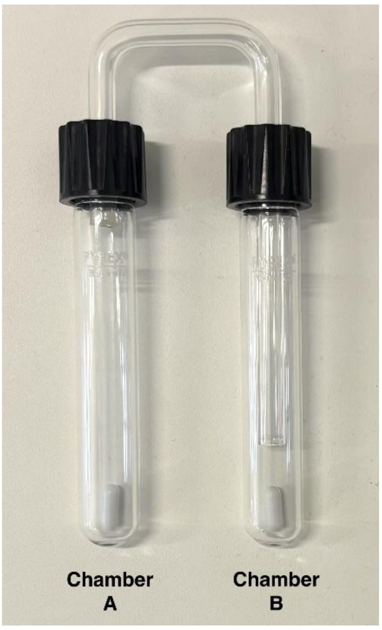

Figure S1. Two chambers photoreactor.

Irradiations were performed in a closed environment equipped with a fan cooling system, with a commercially available Kessil LED PR160L 390 nm, positioned 5 cm from the reactor (Figure S2).

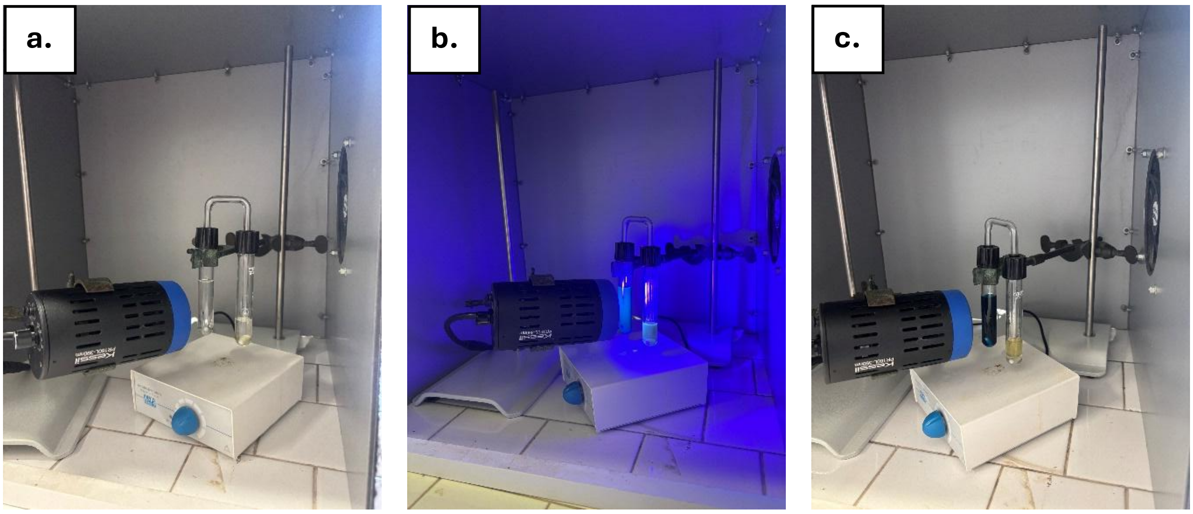

Figure S2. Setup employed for the in-situ Giese reactions and ex-situ Palladium catalyzed aminocarbonylations **a.** before irradiation, **b.** during irradiation, and **c.** after irradiation.

1.3 Chart of the starting materials

Aldehydes

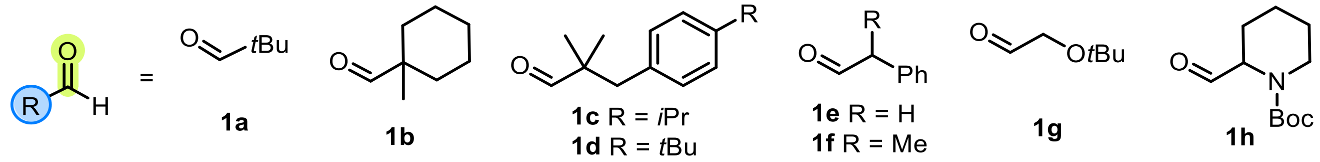

Electron-poor olefins

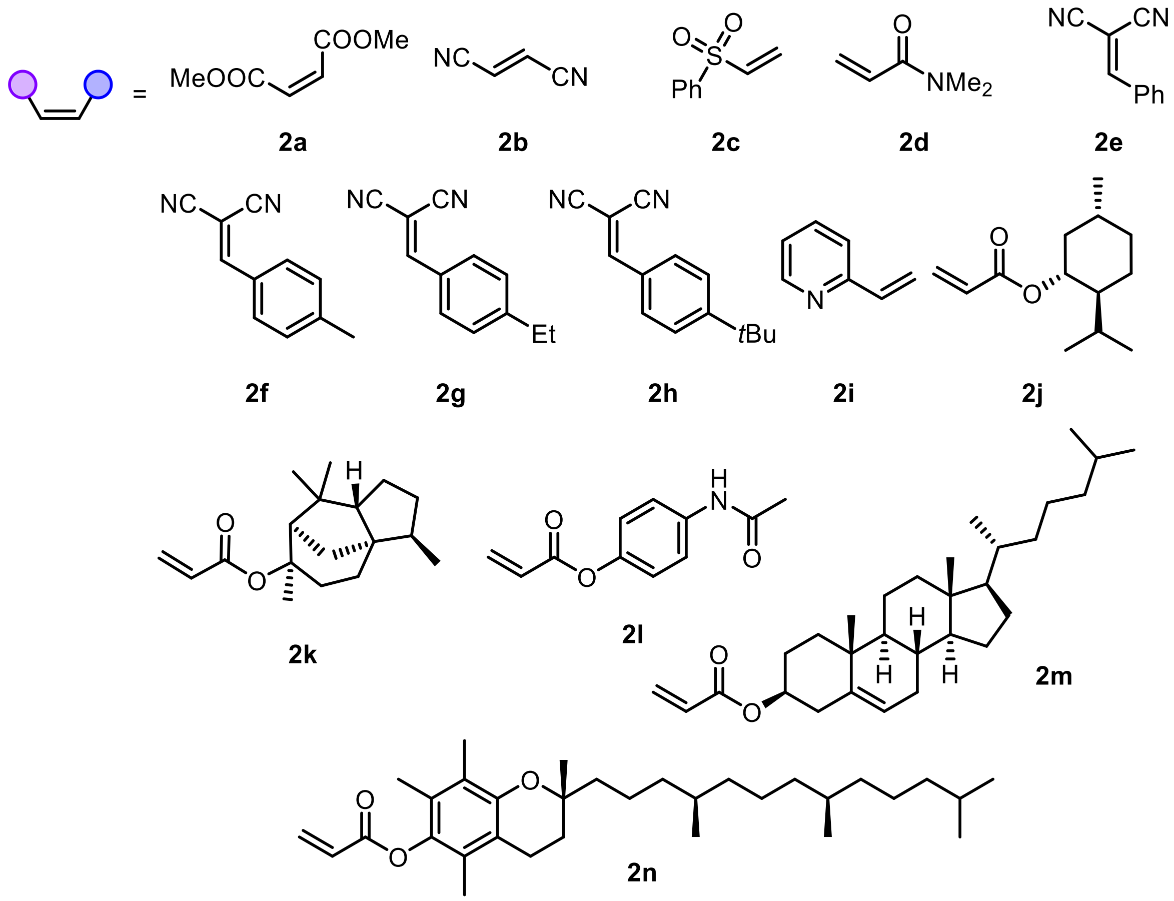

Aryl iodides

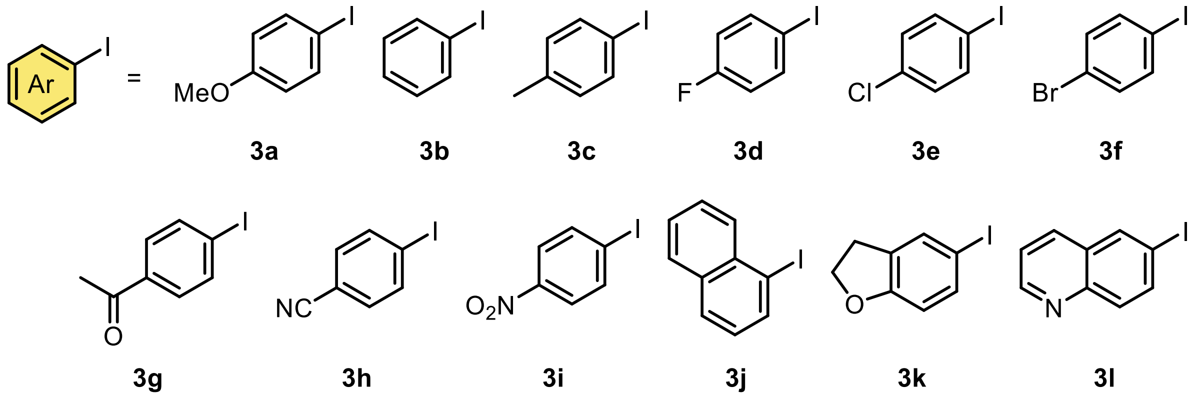

Amines

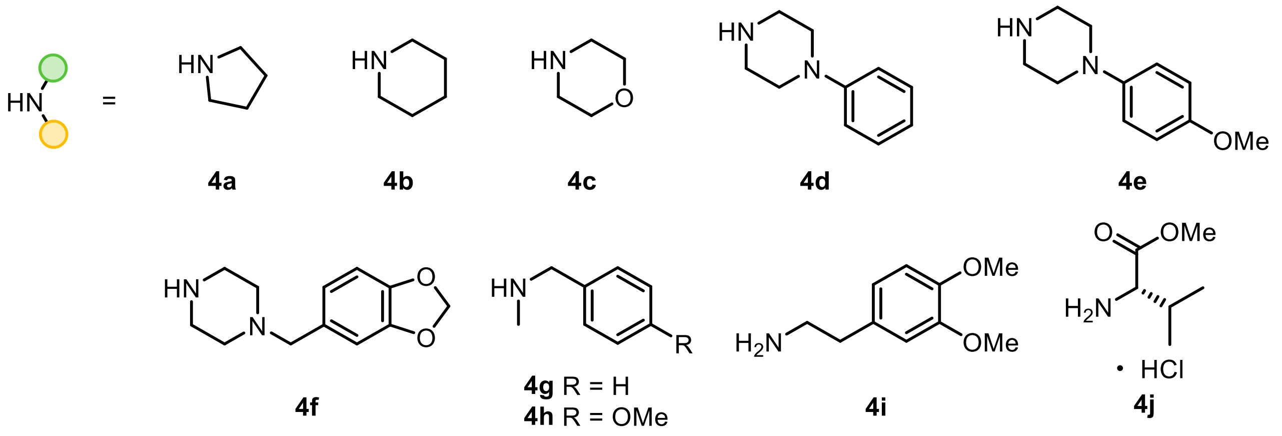

1.4 Absorption spectra of the Pd catalytic system

Stock solutions of the analytes ($Pd(OAc)_2$, bpy and Pd(Bpy)) were prepared by dissolving the appropriate amount of analyte(s) in 5 mL of 2-MeTHF. For the absorption measurements, 2.5 mL of the selected stock solution were placed in a quartz cuvette equipped with a PTFE stopper, purged with N_2 and the spectrum was collected.

A spectrum of the solution of Pd(Bpy) after the addition of 0.063 mmol of pyrrolidine was also measured, showing a blue-shift of the band relative to the d-d transition of the metal center from 400 nm towards 350 nm, possibly related to the reduction of Pd(II) to Pd(0). An immediate change in the color of the solution, from yellow to colorless also occurred. (**Figure S3b**).

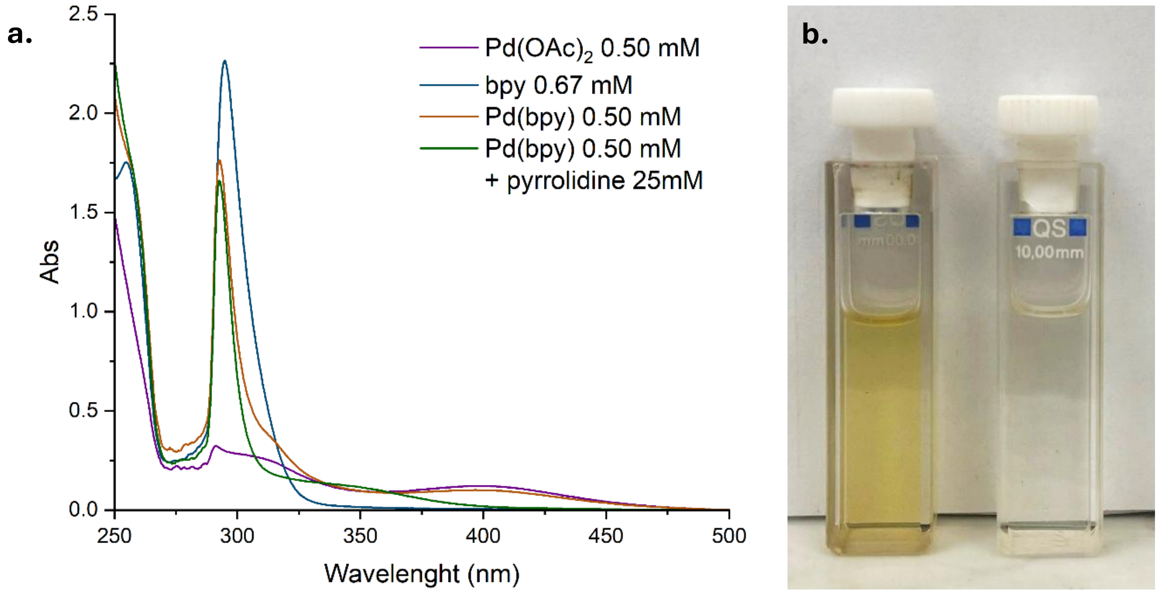

Figure S3. a. UV-Vis spectra of the Pd catalytic system **b.** Pd(Bpy) solution before and after the addition of pyrrolidine.

1.5 Optimization of the reaction conditions

The optimization of the reaction conditions for Chamber B was carried out by studying the coupling between 4-iodoanisole (**3a**, 0.3 mmol) and pyrrolidine (**4a**, 3 equiv.), to give (4-methoxyphenyl)(pyrrolidin-1-yl)methanone (**5B**).

Pivaldehyde (**1a**, 0.9 mmol) was employed as the CORM in the optimization, with dimethyl maleate (**2a**, 0.6 mmol) as the radical trap, to give dimethyl 2-(*tert*-butyl)succinate (**5A**).

Table S1. Optimization experiments

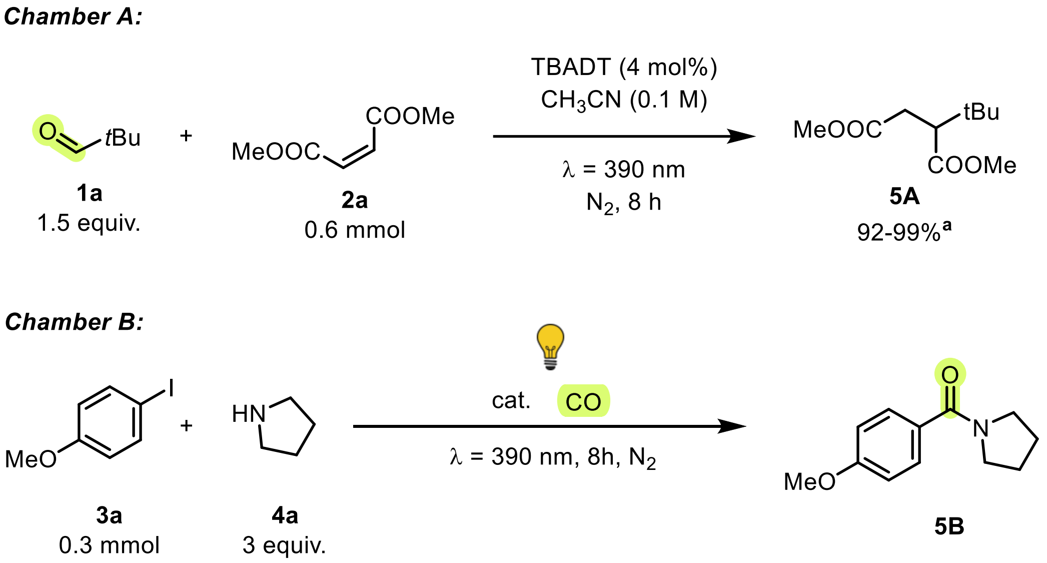

Entry	Catalyst	Additive(s)	Base	Solvent	λ (nm)	Yield[b]
1	$Pd(PPh_3)_4$ (6 mol%)	/	K_2CO_3 (1 equiv.)	2-MeTHF:H_2O 7:3	390	81%
2	$Pd(OAc)_2$ (6 mol%)	/	K_2CO_3 (1 equiv.)	2-MeTHF:H_2O 7:3	390	62%
3	$Pd(OAc)_2$ (2 mol%)	bpy (2.5 mol%)	K_2CO_3 (1 equiv.)	2-MeTHF:H_2O 7:3	390	26%
4	$Pd(OAc)_2$ (4 mol%)	bpy (5 mol%)	K_2CO_3 (1 equiv.)	2-MeTHF:H_2O 7:3	390	57%
**5**	**$Pd(OAc)_2$ (6 mol%)**	**bpy (8 mol%)**	**K_2CO_3 (1 equiv.)**	**2-MeTHF:H_2O 7:3**	**390**	**84% (78%)**
6	$Pd(OAc)_2$ (8 mol%)	bpy (12 mol%)	K_2CO_3 (1 equiv.)	2-MeTHF:H_2O 7:3	390	82%
7	$Pd(OAc)_2$ (6 mol%)	bpy (8 mol%)	KOH (1 equiv.)	2-MeTHF:H_2O 7:3	390	23%

8	$Pd(OAc)_2$ (6 mol%)	bpy (8 mol%)	TEA (1 equiv.)	2-MeTHF:H_2O 7:3	390	53%
9	$Pd(OAc)_2$ (6 mol%)	bpy (8 mol%)	*i*-Pr_2NEt (1 equiv.)	2-MeTHF:H_2O 7:3	390	42%
10	$Pd(OAc)_2$ (6 mol%)	bpy (8 mol%)	K_2CO_3 (1 equiv.)	THF:H_2O 7:3	390	49%
11	$Pd(OAc)_2$ (6 mol%)	bpy (8 mol%)	K_2CO_3 (1 equiv.)	MeCN:H_2O 7:3	390	44%
12	$Pd(OAc)_2$ (6 mol%)	bpy (8 mol%)	K_2CO_3 (1 equiv.)	Acetone:H_2O 7:3	390	38%
13	$Pd(OAc)_2$ (6 mol%)	bpy (8 mol%)	K_2CO_3 (1 equiv.)	Dioxane:H_2O 7:3	390	37%
14	$Pd(OAc)_2$ (6 mol%)	bpy (8 mol%)	K_2CO_3 (1 equiv.)	2-MeTHF:H_2O 7:3	[c]	28%
15	/	/	K_2CO_3 (1 equiv.)	2-MeTHF:H_2O 7:3	390	nr
16	$Pd(OAc)_2$ (6 mol%)	bpy (8 mol%), TEMPO (4 equiv.)	K_2CO_3 (1 equiv.)	2-MeTHF:H_2O 7:3	390	15%
17	$Pd(OAc)_2$ (6 mol%)	bpy (8 mol%)	/	2-MeTHF:H_2O 7:3	390	56%
18[d]	$Pd(OAc)_2$ (6 mol%)	bpy (8 mol%)	K_2CO_3 (1 equiv.)	2-MeTHF:H_2O 7:3	390	nr
19	$Pd(OAc)_2$ (6 mol%)	bpy (8 mol%), TEMPO (4 equiv.)	K_2CO_3 (1 equiv.)	2-MeTHF:H_2O 7:3	[c]	nr
20[e]	$Pd(OAc)_2$ (6 mol%)	bpy (8 mol%)	K_2CO_3 (1 equiv.)	2-MeTHF:H_2O 7:3	390	24%

[a] GC yields determined by using undecane as the external STD. [b] GC yields determined by using 1,3,5 trimethoxybenzene as the external STD (in parenthesis isolated yield). [c] Chamber B was covered with an aluminium foil. [d] *p*-bromoanisole was used instead of *p*-iodoanisole. [e] O_2 was bubbled into Chamber B for 5 min before the irradiation.

1.6 Light On-Off experiments

Light on-off experiments were also carried out to investigate the role of the light in the reaction mechanism, employing the same model reactions already mentioned in paragraph **1.5**. The investigation revealed that the carbonylative conversion of **3a** and **4a** to **5B** significantly increased only during the light-on period.

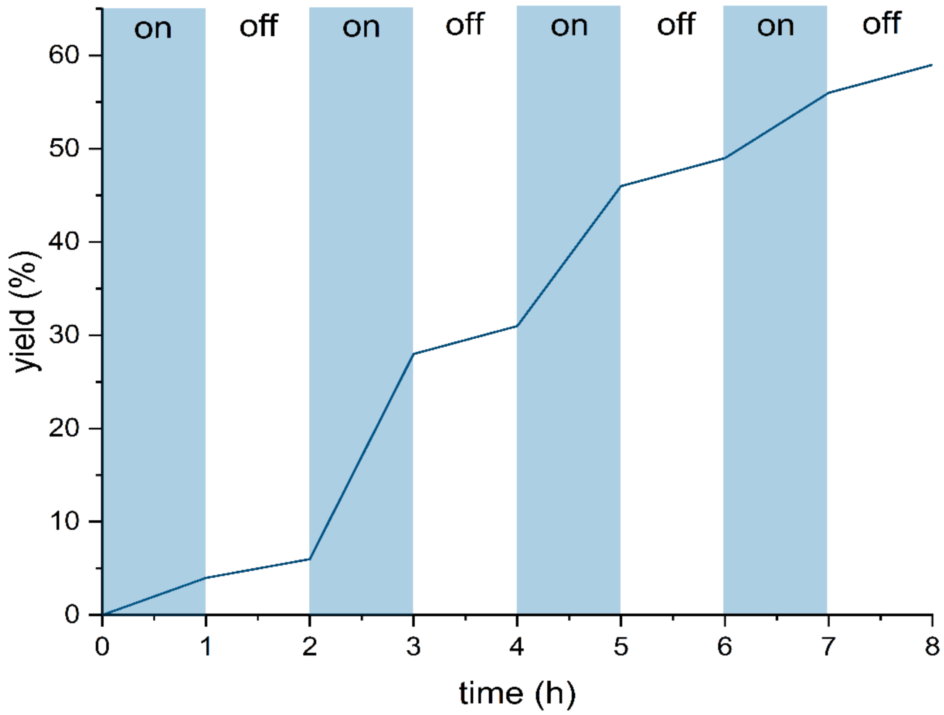

Figure S4. Light on–off experiment for the aminocarbonylation of 4-iodoanisole (**3a**) with pyrrolidine (**4a**). GC yields determined by using 1,3,5 trimethoxybenzene as the external STD. During the light-off periods, Chamber B was covered with an aluminium foil.

1.7 Experimental procedures

1.7.1 Synthesis of aldehydes 1b-1d

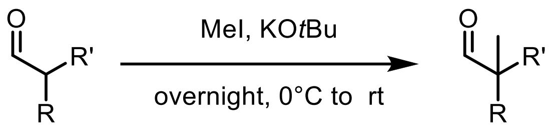

Tertiary aldehydes **1b-d** were prepared according to known procedures.[S1,S2] To a well stirred solution of the selected secondary aldehyde (3 mmol) in 15 mL of the proper solvent (DCM for **1b**, THF in the case of **1c-1d**) at 0 °C was added potassium *tert*-butoxide (438 mg, 3.9 mmol, 1.3 equiv.) in one portion, followed by methyl iodide (564 mL, 9 mmol, 3.0 equiv.). After 1h the reaction mixture was brought to rt and stirred till completion (monitored by TLC). It was then poured into brine; the organic layer was then dried on $MgSO_4$ and the solvent removed in vacuo, affording a light-yellow oil, which was then filtered on a pad of silica (eluant Hex:EtOAc 95:5), to obtain the desired aldehydes **1b-1d**.

1-Methylcyclohexane-1-carbaldehyde (1b)

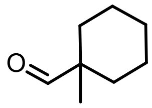

Pale yellow oil, 169.6 mg, 45% yield.

Spectroscopic data are in accordance with literature.[S1]

3-(4-Isopropylphenyl)-2,2-dimethylpropanal (1c)

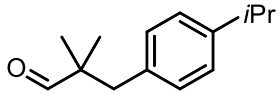

Pale yellow oil, 295.3 mg, 48% yield.

Spectroscopic data are in accordance with literature.[S2]

3-(4-(*tert*-Butyl)phenyl)-2,2-dimethylpropanal (1d)

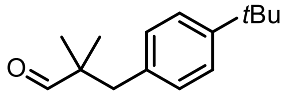

Pale yellow oil, 333.5 mg, 51% yield.

Spectroscopic data are in accordance with literature.[S2]

1.7.2 Synthesis of aldehyde 1g

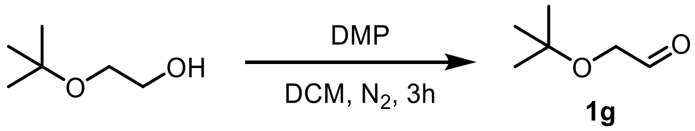

1g

Aldehyde **1g** was prepared following a procedure previously reported.[S3] In a flame-dried 100 mL round-bottomed flask 2-*tert*-butoxyethanol (6.8 mmol, 800 μL) was dissolved in 30 mL of dry DCM. Dess-Martin Periodinane (DMP, 3.2 g, 1.11 equiv.) was added, and the mixture was stirred at rt for 3 h under N_2 atmosphere. The reaction was quenched by 1:1 dilution with Et_2O, followed by addition of an aqueous saturated solution of $NaHCO_3$ and $Na_2S_2O_3$ (1:7). The layers were separated, and the aqueous one was extracted three times with 20 mL of Et_2O. The combined organic layers were washed with brine and dried over Na_2SO_4 before elimination of the solvent under reduced pressure. The crude product was purified by silica gel column chromatography (Pentane/ Et_2O 4:1), to give 340 mg of aldehyde **1g** (44% yield) as a colourless oil.

Spectroscopic data are in accordance with literature.[S3]

1.7.3 General procedure for the synthesis of electron-poor styrenes 2f-h

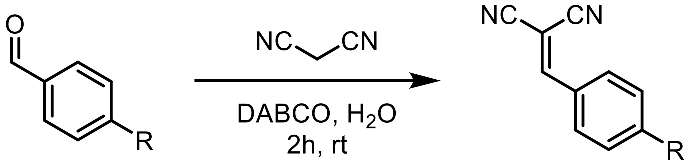

Electron-poor styrene **2f** was prepared following a procedure previously reported;[S4,S5] the same method was also adapted for the synthesis of **2g** and **2h**. In particular, the chosen aromatic aldehyde (6 mmol) malononitrile (1.0 equiv.) in the presence of 10 mol% of 1,4-diazabicyclo[2.2.2]octane (DABCO) was dissolved in water (1.0 M). After 2h under vigorous stirring at room temperature, the solid precipitated was filtered, washed with water and recrystallized from n-hexane.

2-(4-Methylbenzylidene)malononitrile (2f)

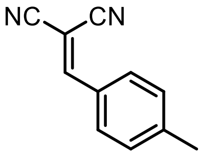

2f was obtained from 4-methylbenzaldehyde, following the above-mentioned procedure.

White solid, 889.6 mg, 88% yield.

Characterization data are in accordance with literature.[S4]

2-(4-Ethylbenzylidene)malononitrile (2g)

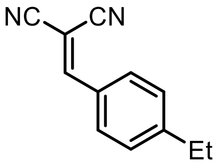

2g was obtained from 4-ethylbenzaldehyde, following the above-mentioned procedure

Pale yellow solid, 915.5 mg, 84% yield.

Characterization data are in accordance with literature[S6]

2-(4-*tert*-Butylbenzylidene)malononitrile 2h

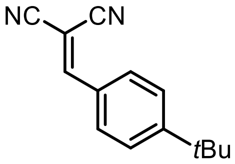

2h was obtained from 4-*tert*-butylbenzaldehyde, following the above-mentioned procedure

Pale yellow solid, 1.04 mg, 83% yield

Characterization data are in accordance with literature.[S7]

1.7.4 General procedure for the synthesis of acrylates 2j-2n

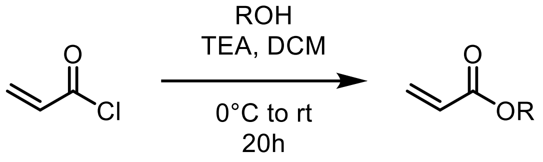

Acrylates **2j-2n** were prepared following a procedure previously reported in the literature.[S8]

At 0 °C, triethylamine (842 µL, 1.5 equiv.) was added dropwise to a solution of the chosen alcohol (1.2 equiv.) in 50 mL of dichloromethane. After 15 min, acryloyl chloride (322 µL, 2.0 mmol) was added to the reaction mixture, which was subsequently allowed to warm up to room temperature. After 20 h, the solvent was concentrated under reduced pressure; the obtained residue was redissolved in ethyl acetate. The insoluble impurities were filtered off on fritted glass, and the filtrate was concentrated and purified by silica gel flash column chromatography (eluant: Hex/EtOAc).

2-Isopropyl-5-methylcyclohexyl acrylate)2j)

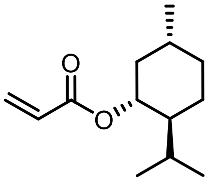

2j was obtained from (-)-menthol, following the above-mentioned procedure, and purified by flash column chromatography on silica gel (Hex:EtOAc 9:1). Colorless oil, 400.9 mg, 95% yield.

Spectroscopic data are in accordance with literature.[S8]

(3R,3aS,6R,7R,8aS)-3,6,8,8-Tetramethyloctahydro-1H-3a,7-methanoazulen-6-yl acrylate (2k)

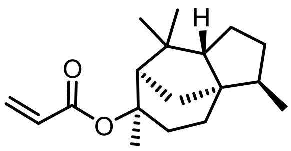

2k was obtained from cedrol, following the above-mentioned procedure, and purified by flash column chromatography on silica gel (Hex:EtOAc 98:2).

Pale yellow oil, 377.3 mg, 68% yield.

Spectroscopic data are in accordance with literature.[S8]

4-Acetamidophenyl acrylate (2l)

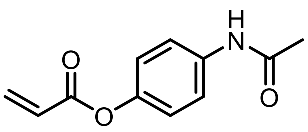

2l was obtained from *p*-acetamidophenol, following the above-mentioned procedure, and purified by flash column chromatography on silica gel (Hex:EtOAc 7:3).

White solid, 344.2 mg, 84% yield.

Characterization data are in accordance with literature.[S8]

(*3S,8S,9S,10R,13R,14S,17R*)-10,13-Dimethyl-17-((R)-6-methylheptan-2-yl)-2,3,4,7,8,9,10,11,12,13,14,15,16,17 tetradecahydro-1H-cyclopenta[a]phenanthren-3-yl acrylate (2m)

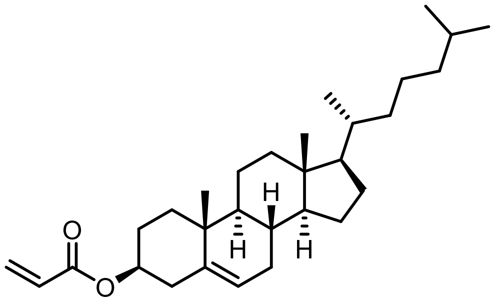

2m was obtained from cholesterol, following the above-mentioned procedure, and purified by flash column chromatography on silica gel (Hex:EtOAc 98:2).

White solid, 451.5 mg, 51% yield.

Characterization data are in accordance with literature.[S8]

(*R*)-2,5,7,8-Tetramethyl-2-((4R,8R)-4,8,12-trimethyltridecyl)chroman-6-yl acrylate (2n)

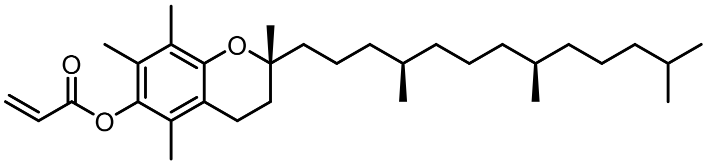

2n was obtained from tocopherol, following the above-mentioned procedure, and purified by flash column chromatography on silica gel (Hex:EtOAc 9:1).

Yellow oil, 878.3 mg, 91% yield.

Spectroscopic data are in accordance with literature.[S8]

1.7.5 General procedure for photochemical reactions (see Figure S1)

Chamber A - CO-Producing/Giese reaction Chamber: Chamber A, equipped with a stirring bar, was loaded with the desired SOMOphile (**2a-2n**, 0.6 mmol, 2 equiv., 0.1 M), the aldehyde (**1a-1h**, 0.9 mmol, 3 equiv.), TBADT (4 mol %) and 6 mL of MeCN. The chamber was purged with N_2 and then irradiated for 8 h at 390 nm.

Purification of the products was performed by flash column chromatography (Hex:EtOAc as the eluants).

Chamber B - Amino Carbonylation Chamber: Chamber B, equipped with a stirring bar, was loaded with a mixture containing the chosen alkyl iodide (**3a-3l**, 0.3 mmol, 1 equiv., 0.12 M), $Pd(OAc)_2$ (6 mol %), bpy (8 mol %) K_2CO_3, (0.3 mmol, 1 equiv.), freshly distilled 2-MeTHF (1.75

mL), the selected amine (**4a-4j**, 0.9 mmol, 3 equiv.), and water (750 μL). The chamber was purged with N_2 and then irradiated for 8 h at 390 nm.

The reaction mixture was extracted twice with DCM and directly adsorbed on silica, to perform flash column chromatography (Hex:EtOAc or DCM:MeOH as the eluants).

1.7.6 Synthesis of 5A/5B on a 1 mmol scale

The photoreactor employed for the 1 mmol scale photochemical reactions consists of two pyrex vessels (diameter 2.5 cm), equipped with stirring bars, closed with SVL hole screw caps (with sealing rings), linked with a glass bridge (diameter 4 mm, Figure S5).

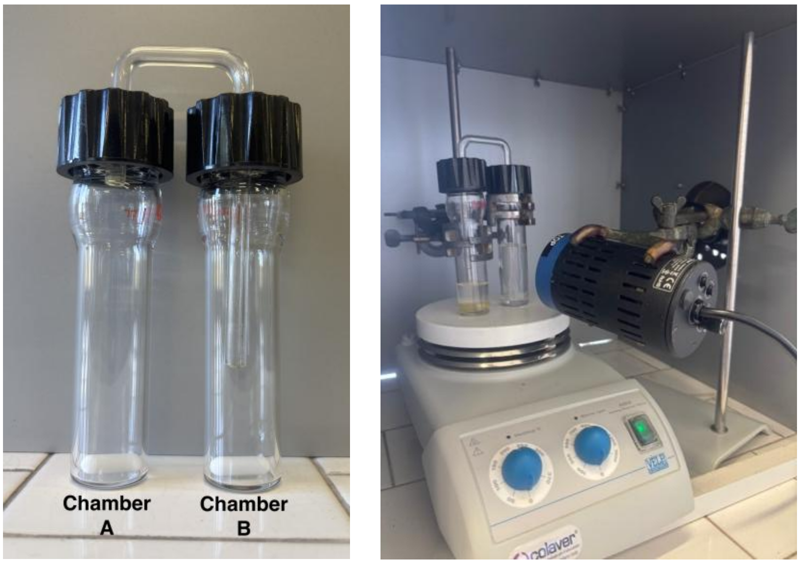

Figure S5. Setup employed for the 1 mmol scale reaction.

Chamber A - CO-Producing/Giese reaction Chamber: Chamber A, equipped with a stirring bar, was loaded with dimethyl maleate **2a** (250 μL, 2 mmol, 2 equiv., 0.1 M), pivaldehyde **1a** (326 μL, 3 mmol, 3 equiv.), TBADT (267 mg, 4 mol %) and 20 mL of MeCN. The chamber was purged with N_2 and then irradiated for 8 h at 390 nm.

Purification of the product was performed by flash column chromatography (Hex:EtOAc 9:1), to afford product **5A** as a colorless oil (360.7 mg. 89% yield after isolation).

Chamber B - Amino Carbonylation Chamber: Chamber B, equipped with a stirring bar, was loaded with a mixture containing 4-iodoanisole **3a** (234 mg, 1 mmol, 1 equiv., 0.12 M), $Pd(OAc)_2$ (13.5 mg, 6 mol %), bpy (12.5 mg, 8 mol %) K_2CO_3, (138 mg, 1 mmol, 1 equiv.), freshly distilled 2-MeTHF (5.8 mL), pyrrolidine **4a** (246 μL, 3 mmol, 3 equiv.), and water (2500 μL). The chamber was purged with N_2 and then irradiated for 8 h at 390 nm.

The reaction mixture was extracted twice with DCM and directly adsorbed on silica, to perform flash column chromatography (Hex/EtOAc 3:2) to afford product **5B** as a pale yellow solid (142.5 mg, 70% yield after isolation).

1.8 Characterization data for products 5A-24A

Dimethyl 2-(*tert*-butyl)succinate (5A)

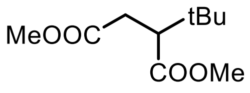

The crude was purified by flash chromatography on silica (Hex/EtOAc 9:1) to afford the product as a colorless oil (111.5 mg, 92% yield after isolation).

^{1}H NMR (300 MHz, Chloroform-*d*) δ 3.67 (s, 3H), 3.63 (s, 3H), 2.93–2.32 (m, 3H), 0.94 (s, 9H).

^{13}C NMR (75 MHz, Chloroform-*d*) δ 174.8, 173.3, 51.9, 51.5, 51.3, 32.7, 27.9.

Spectroscopic data are in accordance with literature.[S9]

2-(*tert*-Butyl)succinonitrile (6A)

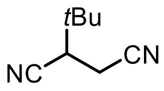

The crude was purified by flash chromatography on silica (Hex/EtOAc 9:1) to afford the product as a pale yellow oil (76.9 mg, 94% yield after isolation).

^{1}H NMR (300 MHz, Chloroform-*d*) δ 3.01–2.34 (m, 3H), 1.13 (s, 9H).

^{13}C NMR (75 MHz, Chloroform-*d*) δ 118.3, 116.6, 40.8, 33.7, 27.1, 17.2.

Spectroscopic data are in accordance with literature.[S10]

((3,3-Dimethylbutyl)sulfonyl)benzene (7A)

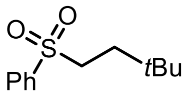

The crude was purified by flash chromatography on silica (Hex/EtOAc 8:2) to afford the product as a pale yellow oil (122.0 mg, 90% yield after isolation).

^{1}H NMR (300 MHz, Chloroform-*d*) δ 7.94–7.87 (m, 2H), 7.70–7.51 (m, 3H), 3.21–2.90 (m, 2H), 1.79–1.45 (m, 2H), 0.85 (s, 9H).

^{13}C NMR (75 MHz, Chloroform-*d*) δ 139.3, 133.7, 129.4, 128.1, 53.0, 35.7, 30.1, 29.0.

Spectroscopic data are in accordance with literature.[S9]

N,N,4,4-Tetramethylpentanamide (8A)

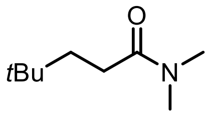

The crude was purified by flash chromatography on silica (Hex/EtOAc 8:2) to afford the product as a colorless oil (82.8 mg, 88% yield after isolation).

^{1}H NMR (300 MHz, Chloroform-*d*) δ 3.00 (s,3H), 2.93 (s, 3H), 2.40–2.16 (m, 2H), 1.75–1.41 (m, 2H), 0.90 (s, 9H).

^{13}C NMR (75 MHz, Chloroform-*d*) δ 173.9, 39.0, 37.5, 35.6, 30.2, 29.2, 29.2.

Spectroscopic data are in accordance with literature.[S9]

2-(2,2-Dimethyl-1-phenylpropyl)malononitrile (9A)

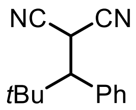

The crude was purified by flash chromatography on silica (Hex/EtOAc 95:5) to afford the product as a colorless oil (100.3 mg, 79% yield after isolation).

^{1}H NMR (300 MHz, Chloroform-*d*) δ 7.39 (s, 5H), 4.22 (d, *J* = 5.7 Hz, 1H), 3.01 (d, *J* = 5.7 Hz, 1H), 1.11 (s, 9H).

^{13}C NMR (75 MHz, Chloroform-*d*) δ 136.4, 129.4, 128.8, 128.7, 113.3, 113.2, 56.9, 35.1, 28.6, 25.2.

Spectroscopic data are in accordance with literature.[S9]

2-(1-(4-(Tolyl)-2,2-dimethylpropyl)malononitrile (10A)

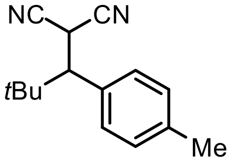

The crud e was purified by flash chromatography on silica (Hex/EtOAc 98:2) to afford the product as a colorless oil (101.0 mg, 74% yield after isolation).

^{1}H NMR (300 MHz, Chloroform-*d*) δ 7.30 (d, *J* = 8.4 Hz, 2H), 7.26 (d, *J* = 7.8 Hz, 2H), 4.20 (d, *J* = 5.7 Hz, 1H), 2.98 (d, *J* = 5.7 Hz, 1H), 2.36 (s, 3H), 1.09 (s, 9H).

^{13}C NMR (75 MHz, Chloroform-*d*) δ 138.5, 133.3, 129.5, 129.3, 113.4, 113.3, 56.6, 35.0, 28.6, 25.3, 21.2.

HRMS (ESI) m/z: $[M + H]^+$ Calcd for $C_{15}H_{19}N_2^+$ 227.1548; Found 227.1545.

2-(1-(4-(Ethyl)phenyl)-2,2-dimethylpropyl)malononitrile (11A)

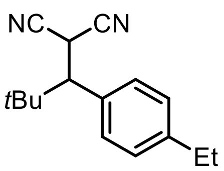

The crude was purified by flash chromatography on silica (Hex/EtOAc 98:2) to afford the product as a pale yellow oil (102.5 mg, 71% yield after isolation).

^{1}H NMR (300 MHz, Chloroform-*d*) δ 7.29 (d, *J* = 8.1 Hz, 2H), 7.21 (d, *J* = 8.2 Hz, 2H), 4.20 (d, *J* = 5.6 Hz, 1H), 2.98 (d, *J* = 5.6 Hz, 1H), 2.67 (q, *J* = 7.6 Hz, 2H), 1.25 (t, *J* = 7.6 Hz, 3H), 1.10 (s, 9H).

^{13}C NMR (75 MHz, Chloroform-*d*) δ 144.7, 133.5, 129.4, 128.3, 113.4, 113.3, 56.7, 35.1, 28.7, 28.6, 25.3, 15.3.

HRMS (ESI) m/z: $[M + H]^+$ Calcd for $C_{16}H_{21}N_2^+$ 241.1705; Found 241.1701.

2-(1-(4-(*tert*-Butyl)phenyl)-2,2-dimethylpropyl)malononitrile (12A)

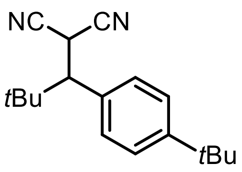

The crude was purified by flash chromatography on silica (Hex/EtOAc 98:2) to afford the product as a pale yellow oil (128.6 mg, 80% yield after isolation).

^{1}H NMR (300 MHz, Chloroform-*d*) δ 7.38 (d, *J* = 8.5 Hz, 2H), 7.30 (d, *J* = 8.5 Hz, 2H), 4.21 (d, *J* = 5.4 Hz, 1H), 2.98 (d, *J* = 5.4 Hz, 1H), 1.32 (s, 9H), 1.10 (s, 9H).

^{13}C NMR (75 MHz, Chloroform-*d*) δ 151.6, 133.2, 129.1, 125.7, 113.4, 113.3, 56.6, 35.2, 34.7, 31.4, 28.7, 25.2.

Spectroscopic data are in accordance with literature.[S11]

2-(3,3-Dimethylbutyl)pyridine (13A)

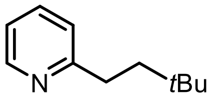

The crude was purified by flash chromatography on silica (Hex/EtOAc 9:1) to afford the product as a colorless oil (93.2 mg, 95% yield after isolation).

^{1}H NMR (300 MHz, Chloroform-*d*) δ 8.60–8.42 (m, 1H), 7.56 (td, *J* = 7.6, 1.9 Hz, 1H), 7.13 (d, *J* = 7.8 Hz, 1H), 7.10–7.03 (m, 1H), 2.85–2.64 (m, 2H), 1.86–1.52 (m, 2H), 0.96 (s, 9H).

^{13}C NMR (75 MHz, Chloroform-*d*) δ 163.3, 149.3, 136.4, 122.7, 120.9, 44.4, 34.0, 30.6, 29.4.

Spectroscopic data are in accordance with literature.[S9]

(*1R,2S,5R*)-2-Isopropyl-5-methylcyclohexyl 4,4-dimethylpentanoate (14A)

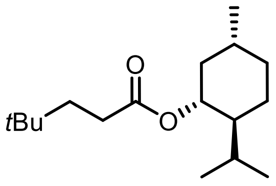

The crude was purified by flash chromatography on silica (Hex/EtOAc 95:5) to afford the product as a colorless oil (110.6 mg, 69% yield after isolation).

^{1}H NMR (300 MHz, Chloroform-*d*) δ 4.67 (td, *J* = 10.8, 4.4 Hz, 1H), 2.33–2.14 (m, 2H), 2.06–1.79 (m, 2H), 1.74–1.29 (m, 6H), 1.15–0.80 (m, 18H), 0.75 (d, *J* = 7.0 Hz, 3H).

^{13}C NMR (75 MHz, Chloroform-*d*) δ 174.2, 74.0, 47.2, 41.1, 38.8, 34.4, 31.5, 30.6, 30.2, 29.2, 26.4, 23.6, 22.2, 20.9, 16.5.

HRMS (ESI) m/z: $[M + Na]^+$ Calcd for $C_{17}H_{32}NaO_2^+$ 291.2300; Found 291.2296.

(*3R,3aS,6R,7R,8aS*)-3,6,8,8-tetramethyloctahydro-1H-3a,7-methanoazulen-6-yl 4,4-dimethylpentanoate (15A)

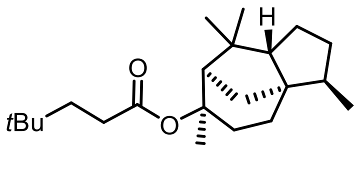

The crude was purified by flash chromatography on silica (Hex/EtOAc 98:2) to afford the product as a colorless oil (148.7 mg, 74% yield after isolation).

¹H NMR (300 MHz, Chloroform-*d*) δ 2.38 (d, *J* = 5.2 Hz, 1H), 2.28–1.24 (m, 17H), 1.16 (s, 3H), 0.98 (s, 2H), 0.89–0.81 (m, 15H).

¹³C NMR (75 MHz, Chloroform-*d*) δ 173.7, 86.3, 57.2, 56.9, 54.1, 43.5, 41.5, 41.2, 38.6, 37.1, 33.3, 31.7, 31.4, 30.1, 29.2, 28.6, 27.3, 26.0, 25.4, 15.7.

HRMS (ESI) m/z: $[M + Na]^+$ Calcd for $C_{22}H_{38}NaO_2^+$ 357.2769; Found 357.2765.

4-Acetamidophenyl 4,4-dimethylpentanoate (16A)

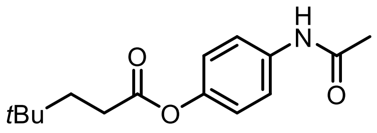

The crude was purified by flash chromatography on silica (Hex/EtOAc 8:2) to afford the product as a white solid (87.2 mg, 55% yield after isolation).

Melting point: 148-151 °C

¹H NMR (300 MHz, Chloroform-*d*) δ 7.49 (d, *J* = 8.9 Hz, 2H), 7.02 (d, *J* = 8.9 Hz, 2H), 2.57–2.45 (m, 2H), 2.16 (s, 3H), 1.74–1.63 (m, 2H), 0.95 (s, 9H).

¹³C NMR (75 MHz, Chloroform-*d*) δ 173.2, 168.3, 147.1, 135.6, 122.1, 120.9, 38.7, 30.4, 30.3, 29.2, 24.7.

HRMS (ESI) m/z: $[M + H]^+$ Calcd for $C_{15}H_{22}NO_3^+$ 264.1600; Found 264.1593

***(3S,8S,9S,10R,13R,14S,17R)*-10,13-dimethyl-17-((R)-6-methylheptan-2-yl)-2,3,4,7,8,9,10,11,12,13,14,15,16,17-tetradecahydro-1H-cyclopenta[a]phenanthren-3-yl 4,4-dimethylpentanoate (17A)**

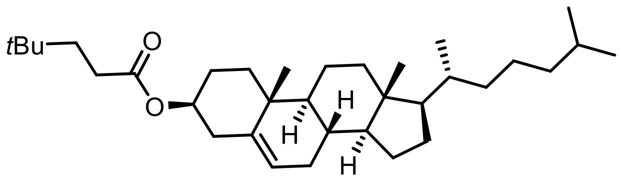

The crude was purified by flash chromatography on silica (Hex/EtOAc 98:2) to afford the product as a white solid (130.7 mg, 44% yield after isolation).

Melting point: 93-96 °C

¹H NMR (300 MHz, Chloroform-*d*) δ 5.37 (d, *J* = 5.0 Hz, 1H), 4.69–4.53 (m, 1H), 2.37–2.19 (m, 4H), 2.00 (m, 2H), 1.91–1.74 (m, 3H), 1.63–0.81 (m, 44H), 0.68 (s, 3H).

¹³C NMR (75 MHz, Chloroform-*d*) δ 174.0, 139.9, 122.7, 73.9, 56.8, 56.3, 50.2, 42.5, 39.9, 39.7, 38.8, 38.3, 37.2, 36.8, 36.3, 35.9, 32.1, 32.0, 30.7, 30.2, 29.2, 28.4, 28.2, 27.9, 24.4, 23.4, 23.0, 22.7, 21.2, 19.5, 18.9, 12.0.

HRMS (ESI) m/z: $[M + Na]^+$ Calcd for $C_{34}H_{58}NaO_2^+$ 521.4335; Found 521.4331.

(*R*)-2,5,7,8-Tetramethyl-2-((4R,8R)-4,8,12-trimethyltridecyl)chroman-6-yl 4,4-dimethylpentanoate (18A)

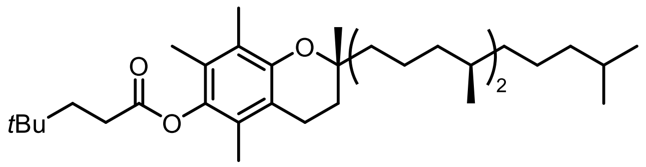

The crude was purified by flash chromatography on silica (Hex/EtOAc 9:1) to afford the product as a yellow oil (152.5 mg, 47% yield after isolation).

^{1}H NMR (300 MHz, Chloroform-*d*) δ 2.66–2.51 (m, 4H), 2.09 (s, 3H), 2.01 (s, 3H), 1.97 (s, 3H), 1.87–1.68 (m, 4H), 1.54 –1.01 (m, 24H), 0.97 (s, 9H), 0.91–0.80 (m, 12H).

^{13}C NMR (75 MHz, Chloroform-*d*) δ 173.1, 149.5, 140.6, 126.8, 125.1, 123.9, 117.5, 75.2, 39.5, 39.0, 37.7, 37.6, 37.5, 37.4, 32.9, 32.8, 31.2, 30.3, 30.91, 29.2, 28.1, 25.0, 24.6, 22.9, 22.8, 21.1, 20.8, 19.9, 19.8, 19.7, 13.1, 12.3, 12.0.

Spectroscopic data are in accordance with literature.[S12]

2-(1-Methylcyclohexyl)succinonitrile (19A)

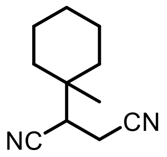

The crude was purified by flash chromatography on silica (Hex/EtOAc 9:1) to afford the product as a pale yellow oil (76.3 mg, 72% yield after isolation).

^{1}H NMR (300 MHz, Chloroform-*d*) δ 2.87 (dd, *J* = 8.9, 5.7 Hz, 1H), 2.70–2.58 (m, 2H), 1.70–1.23 (m, 10H), 1.07 (s, 3H).

^{13}C NMR (75 MHz, Chloroform-*d*) δ 118.3, 116.8, 40.2, 36.1, 36.0, 35.0, 25.6, 21.7, 21.5, 20.6, 16.2.

HRMS (ESI) m/z: $[M + Na]^+$ Calcd for $C_{11}H_{16}N_2Na^+$ 199.1211; Found 199.1205.

2-(1-(4-Isopropylphenyl)-2-methylpropan-2-yl)succinonitrile (20A)

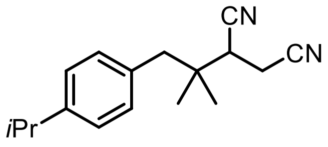

The crude was purified by flash chromatography on silica (Hex/EtOAc 9:1) to afford the product as a yellowish oil (94.5 mg, 64% yield after isolation).

^{1}H NMR (300 MHz, Chloroform-*d*) δ 7.19 (d, *J* = 8.1 Hz, 2H), 7.09 (d, *J* = 8.1 Hz, 2H), 2.90 (hept, *J* = 7.1 Hz, 1H), 2.84–2.73 (m, 2H), 2.69–2.58 (m, 3H), 1.27 (s, 3H), 1.24 (s, 3H), 1.15 (s, 3H), 1.04 (s, 3H).

^{13}C NMR (75 MHz, Chloroform-*d*) δ 147.9, 133.1, 130.4, 126.7, 118.5, 116.7, 46.1, 38.5, 37.3, 33.8, 24.6, 24.2, 24.1, 17.0.

HRMS (ESI) m/z: $[M + H]^+$ Calcd for $C_{17}H_{23}N_2^+$ 255.1861; Found 255.1857.

2-(1-(4-(*tert*-Butyl)phenyl)-2-methylpropan-2-yl)succinonitrile (21A)

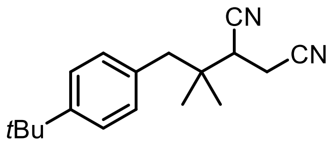

The crude was purified by flash chromatography on silica (Hex/EtOAc 9:1) to afford the product as a yellowish oil (99.6 mg, 62% yield after isolation).

^{1}H NMR (300 MHz, Chloroform-*d*) δ 7.34 (d, *J* = 8.3 Hz, 2H), 7.10 (d, *J* = 8.2 Hz, 2H), 2.82–2.74 (m, 2H), 2.69–2.59 (m, 3H), 1.32 (s, 9H), 1.15 (s, 3H), 1.04 (s, 3H).

^{13}C NMR (75 MHz, Chloroform-*d*) δ 150.2, 132.8, 130.2, 125.5, 118.5, 116.6, 46.0, 38.5, 37.4, 34.6, 31.5, 24.6, 24.2, 17.0.

HRMS (ESI) m/z: [M + H]$^+$ Calcd for $C_{18}H_{25}N_2^+$ 269.2018; Found 269.2015.

2-Benzylsuccinonitrile (22A)

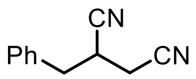

In this case, 6 mL of MeCN:DCM 11:1 were employed as the solvent in chamber A.

The crude was purified by flash chromatography on silica (Hex/EtOAc 8:2) to afford the product as a colorless oil (81.4 mg, 80% yield after isolation).

^{1}H NMR (300 MHz, Chloroform-*d*) δ 7.44–7.20 (m, 5H), 3.19–3.00 (m, 3H), 2.63 (d, *J* = 5.9 Hz, 2H).

^{13}C NMR (75 MHz, Chloroform-*d*) δ 134.5, 129.3, 129.2, 128.3, 118.6, 115.6, 37.1, 30.2, 20.2.

Spectroscopic data are in accordance with literature.[S13]

2-(1-Phenylethyl)succinonitrile (23A)

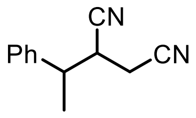

The crude was purified by flash chromatography on silica (Hex/EtOAc 8:2) to afford the product (1:1 mixture of diasteroisomers) as a colorless oil (84.2 mg, 76% yield after isolation).

^{1}H NMR (300 MHz, Chloroform-*d,* Mixture of diastereoisomers) δ 7.49–7.17 (m, 5H), 3.31–2.91 (m, 2H), 2.72–2.33 (m, 2H), 1.60–1.57 (m, 3H).

^{13}C NMR (75 MHz, Chloroform-*d,* Mixture of diastereoisomers) δ 140.3, 138. 9, 129.5, 129.3, 128.5, 128.3, 127.9, 127.1, 118.4, 117.8, 115.9, 115.6, 41.4, 40.1, 36.0, 35.7, 20.1, 19.6, 19.5, 19.2.

Spectroscopic data are in accordance with literature.[S13]

2-(*tert*-Butoxymethyl)succinonitrile (24A)

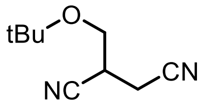

The crude was purified by flash chromatography on silica (Hex/EtOAc 9:1) to afford the product as a pale yellow oil (74.7 mg, 75% yield after isolation).

^{1}H NMR (300 MHz, Chloroform-*d*) δ 3.72–3.56 (m, 2H), 3.09–3.01 (m, 1H), 2.86–2.77 (m, 2H), 1.22 (s, 9H).

^{13}C NMR (75 MHz, Chloroform-*d*) δ 117.7, 115.8, 59.8, 29.7, 27.4, 18.0.

Spectroscopic data are in accordance with literature.[S3]

2-(1-Methylcyclohexyl)succinonitrile (25A)

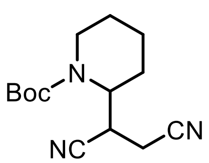

The crude was purified by flash chromatography on silica (Hex/EtOAc 9:1) to afford the product as a pale yellow oil (127.6 mg, 81% yield after isolation).

^{1}H NMR (300 MHz, Chloroform-*d*) δ 4.52 (d, *J* = 10.9 Hz, 1H), 4.08 (bs, 1H), 3.37 (dt, *J* = 10.8, 6.5 Hz, 1H), 2.82 – 2.48 (m, 3H), 2.12 – 2.03 (m, 1H), 1.86 – 1.60 (m, 5H), 1.48 (s, 9H).

^{13}C NMR (75 MHz, Chloroform-*d*) δ 154.8, 117.9, 116.0, 81.5, 51.8, 40.2, 29.1, 28.4, 26.8, 24.7, 19.0, 18.5.

HRMS (ESI) m/z: $[M + H]^+$ Calcd for $C_{14}H_{22}N_3O_2^+$ 264.1712; Found 264.1705.

1.9 Characterization data for products 5B-24B

(4-Methoxyphenyl)(pyrrolidin-1-yl)methanone (5B)

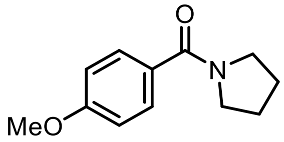

The crude was purified by flash chromatography on silica (Hex/EtOAc 3:2) to afford the product as a pale yellow solid (47.8 mg, 78% yield after isolation).

Melting point: 73-76°C

^{1}H NMR (300 MHz, Chloroform-*d*) δ 7.51 (d, *J* = 8.8 Hz, 2H), 6.89 (d, *J* = 8.7 Hz, 2H), 3.83 (s, 3H), 3.72–3.38 (m,4H), 2.16–1.84 (m, 4H).

^{13}C NMR (75 MHz, Chloroform-*d*) δ 169.6, 160.9, 129.6, 129.3, 113.5, 55.5, 49.9, 46.5, 26.6, 24.6.

Characterization data are in accordance with literature.[S14]

Phenyl(pyrrolidin-1-yl)methanone (6B)

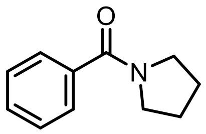

The crude was purified by flash chromatography on silica (Hex/EtOAc 3:2) to afford the product as a colorless oil (40.0 mg, 74% yield after isolation).

^{1}H NMR (300 MHz, Chloroform-*d*) δ 7.54–7.44 (m, 2H), 7.37 (m, 1.9 Hz, 3H), 3.63 (t, *J* = 6.9 Hz, 2H), 3.40 (t, *J* = 6.5 Hz, 2H), 1.99–1.78 (m, 4H).

^{13}C NMR (75 MHz, Chloroform-*d*) δ 169.8, 137.3, 129.8, 128.3, 127.1, 49.7, 46.2, 26.5, 24.5.

Spectroscopic data are in accordance with literature.[S15]

Pyrrolidin-1-yl(p-tolyl)methanone (7B)

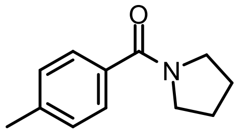

The crude was purified by flash chromatography on silica (Hex/EtOAc 3:2) to afford the product as a pale yellow solid (42.7 mg, 75% yield after isolation).

Melting point: 82-85°C

^{1}H NMR (300 MHz, Chloroform-*d*) δ 7.42 (d, *J* = 8.1 Hz, 2H), 7.18 (d, *J* = 7.9 Hz, 2H), 3.63 (t, *J* = 6.9 Hz, 2H), 3.43 (t, *J* = 6.5 Hz, 2H), 2.37 (s, 3H), 2.14–1.70 (m, 4H).

^{13}C NMR (75 MHz, Chloroform-*d*) δ 170.0, 140.0, 134.5, 128.9, 127.3, 49.8, 46.3, 26.5, 24.6, 21.5.

Characterization data are in accordance with literature.[S15]

(4-Fluorophenyl)(pyrrolidin-1-yl)methanone (8B)

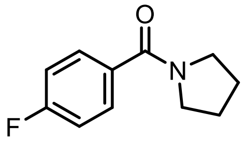

The crude was purified by flash chromatography on silica (Hex/EtOAc 3:2) to afford the product as a white solid (38.6 mg, 67% yield after isolation).

Melting point: 89-91 °C

^{1}H NMR (300 MHz, Chloroform-*d*) δ 7.62–7.44 (m, 2H), 7.17–6.96 (m, 2H), 3.63 (t, *J* = 6.8 Hz, 2H), 3.42 (t, *J* = 6.5 Hz, 2H), 2.07–1.84 (m, 4H)

^{13}C NMR (75 MHz, $CDCl_3$) δ 168.8, 165.2, 161.9, 133.4, 133.4, 129.6, 129.5, 115.5, 115.2, 49.8, 46.4, 26.6, 24.6.

Characterization data are in accordance with literature.[S15]

(4-Chlorophenyl)(pyrrolidin-1-yl)methanone (9B)

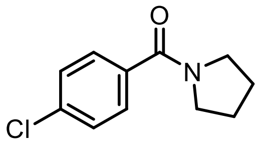

The crude was purified by flash chromatography on silica (Hex/EtOAc 3:2) to afford the product as a white solid (44.1 mg, 70% yield after isolation).

Melting point: 67-71 °C

^{1}H NMR (400 MHz, Chloroform-*d*) δ 7.45 (d, *J* = 8.5 Hz, 2H), 7.36 (d, *J* = 8.5 Hz, 2H), 3.62 (t, *J* = 6.9 Hz, 2H), 3.40 (t, *J* = 6.6 Hz, 2H), 2.09–1.65 (m, 4H).

^{13}C NMR (101 MHz, Chloroform-*d*) δ 168.7, 135.9, 135.7, 128.8, 128.6, 49.7, 46.4, 26.5, 24.5.

Characterization data are in accordance with literature.[S14]

(4-Bromophenyl)(pyrrolidin-1-yl)methanone (10B)

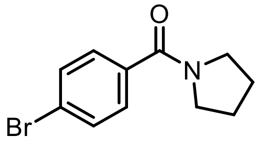

The crude was purified by flash chromatography on silica (Hex/EtOAc 3:2) to afford the product as a pale yellow solid (53.5 mg, 70% yield after isolation).

Melting point: 80-82 °C

^{1}H NMR (300 MHz, Chloroform-*d*) δ 7.53 (d, *J* = 8.5 Hz, 2H), 7.39 (d, *J* = 8.5 Hz, 2H), 3.63 (t, *J* = 6.8 Hz, 2H), 3.40 (t, *J* = 6.5 Hz, 2H), 2.01–1.83 (m, 4H).

^{13}C NMR (75 MHz, Chloroform-*d*) δ 168.7, 136.2, 131.6, 129.0, 124.2, 49.7, 46.4, 26.6, 24.6.

Characterization data are in accordance with literature.[S14]

1-(4-(Pyrrolidine-1-carbonyl)phenyl)ethan-1-one (11B)

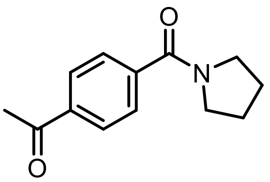

The crude was purified by flash chromatography on silica ((Hex/EtOAc 1:1) to afford the product as a colorless oil (47.3 mg, 73% yield after isolation).

^{1}H-NMR (300 MHz, Chloroform-*d*) δ 7.97 (d, *J* = 8.5 Hz, 2H), 7.58 (d, *J* = 8.5 Hz, 2H), 3.64 (t, *J* = 6.8 Hz, 2H), 3.37 (t, *J* = 6.5 Hz, 2H), 2.61 (s, 3H), 2.04–1.82 (m, 4H).

^{13}C NMR (75 MHz, Chloroform-*d*) δ 197.6, 168.7, 141.6, 137.9, 128.4, 127.4, 49.5, 46.3, 26.8, 26.5, 24.5.

Spectroscopic data are in accordance with literature.[S15]

4-(Pyrrolidine-1-carbonyl)benzonitrile (12B)

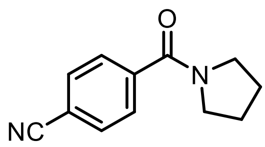

The crude was purified by flash chromatography on silica (Hex/EtOAc 1:1) to afford the product as a white solid (39.2 mg, 65% yield after isolation).

Melting point: 80-82 °C

^{1}H NMR (300 MHz, Chloroform-*d*) δ 7.68 (d, *J* = 8.3 Hz, 2H), 7.59 (d, *J* = 8.3 Hz, 2H), 3.62 (t, *J* = 6.8 Hz, 2H), 3.34 (t, *J* = 6.5 Hz, 2H), 2.13–1.67 (m, 4H).

^{13}C NMR (75 MHz, Chloroform-*d*) δ 167.7, 141.5, 132.3, 127.8, 118.2, 113.6, 49.5, 46.4, 26.4, 24.4.

Characterization data are in accordance with literature.[S15]

(4-Nitrophenyl)(pyrrolidin-1-yl)methanone (13B)

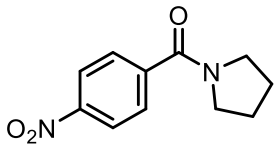

The crude was purified by flash chromatography on silica (Hex/EtOAc 1:1) to afford the product as a pale orange solid (38.5 mg, 58% yield after isolation).

Melting point: 60-61 °C

^{1}H NMR (300 MHz, Chloroform-*d*) δ 8.25 (d, *J* = 8.7 Hz, 2H), 7.67 (d, *J* = 8.7 Hz, 2H), 3.65 (t, *J* = 6.8 Hz, 2H), 3.37 (t, *J* = 6.5 Hz, 2H), 2.09–1.75 (m, 4H).

^{13}C NMR (75 MHz, Chloroform-*d*) δ 167.5, 148.5, 143.3, 128.2, 123.8, 49.5, 46.5, 26.5, 24.5.

Characterization data are in accordance with literature.[S16]

Naphthalen-1-yl(pyrrolidin-1-yl)methanone (14B)

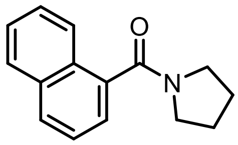

14B

The crude was purified by flash chromatography on silica (Hex/EtOAc 7:3) to afford the product as a pale yellow oil (44.7 mg, 66% yield after isolation).

^{1}H NMR (300 MHz, Chloroform-*d*) δ 7.92–7.75 (m, 3H), 7.60–7.30 (m, 4H), 3.78 (t, *J* = 7.0 Hz, 2H), 3.10 (t, *J* = 6.8 Hz, 2H), 1.98 (quint, *J* = 6.8 Hz, 2H), 1.80 (quint, *J* = 6.7 Hz, 2H).

^{13}C NMR (75 MHz, Chloroform-*d*) δ 169.3, 135.9, 133.6, 129.3, 129.1, 128.4, 127.0, 126.3, 125.2, 125.0, 123.8, 48.5, 45.7, 26.1, 24.7.

Spectroscopic data are in accordance with literature.[S17]

(2,3-Dihydrobenzofuran-5-yl)(pyrrolidin-1-yl)methanone (15B)

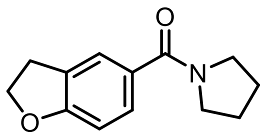

The crude was purified by flash chromatography on silica (Hex/EtOAc 7:3) to afford the product as a pale yellow oil (35.6 mg, 54% yield after isolation).

^{1}H NMR (300 MHz, Chloroform-*d*) δ 7.42 (d, *J* =1.5 Hz, 1H), 7.35–7.24 (m, 1H), 6.75 (d, *J* =8.2 Hz 1H), 4.60 (t, *J* = 8.7 Hz, 2H), 3.66–3.39 (m, 4H), 3.22 (t, *J* = 8.7 Hz, 2H), 2.01–1.81 (m, 4H).

^{13}C-NMR (300 MHz, Chloroform-*d*) δ 169.9, 161.6, 129.6, 128.1, 127.2, 124.8, 108.7, 71.8, 50.0, 46.5, 29.5, 26.6, 24.6.

HRMS (ESI) m/z: $[M + H]^+$ Calcd for $C_{13}H_{16}NO_2^+$ 218.1181; Found 218.1171.

Pyrrolidin-1-yl(quinolin-6-yl)methanone (16B)

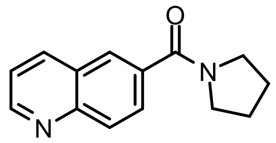

The crude was purified by flash chromatography on silica (DCM/MeOH 98:2) to afford the product as a dark yellow oil (50.1 mg, 74% yield after isolation).

^{1}H NMR (300 MHz, Chloroform-*d*) δ 8.94 (dd, *J* = 4.2, 1.8 Hz, 1H), 8.18 (d, *J* = 7.9 Hz, 1H), 8.12 (d, *J* = 8.7 Hz, 1H), 7.99 (d, *J* = 1.9 Hz, 1H), 7.83 (dd, *J* = 8.7, 1.9 Hz, 1H), 7.42 (dd, *J* = 8.3, 4.2 Hz, 1H), 3.69 (t, *J* = 6.9 Hz, 2H), 3.46 (t, *J* = 6.5 Hz, 2H), 2.02–1.75 (m, 4H).

^{13}C-NMR (75 MHz, Chloroform-*d*) δ 169.0, 151.6, 148.6, 136.7, 135.4, 129.7, 128.1, 127.7, 127.1, 121.8, 49.8, 46.4, 26.5, 24.6.

Spectroscopic data are in accordance with literature.[S18]

(4-Methoxyphenyl)(pyperidin-1-yl)methanone (17B)

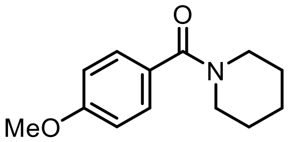

The crude was purified by flash chromatography on silica (Hex/EtOAc 3:2) to afford the product as a pale yellow oil (49.2 mg, 75% yield after isolation).

^{1}H NMR (300 MHz, Chloroform-*d*) δ 7.36 (d, *J* = 8.7 Hz, 2H), 6.89 (d, *J* = 8.7 Hz, 2H), 3.82 (s, 3H), 3.53 (bs, 4H), 1.73–1.53 (m, 6H).

^{13}C NMR (75 MHz, Chloroform-*d*) δ 170.4, 160.6, 129.0, 128.8, 113.7, 55.4, 49.0, 43.9, 26.4, 24.8.

Spectroscopic data are in accordance with literature.[S19]

(4-Methoxyphenyl)(morpholino)methanone (18B)

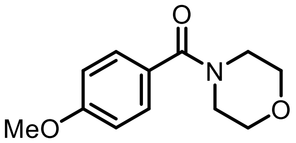

The crude was purified by flash chromatography on silica (Hex/EtOAc 3:2) to afford the product as a colorless oil (41.3 mg, 62% yield after isolation).

^{1}H-NMR (300 MHz, Chloroform-*d*) δ 7.38 (d, *J* = 8.7 Hz, 2H), 6.91 (d, *J* = 8.7 Hz, 2H), 3.83 (s, 3H), 3.66 (m, 8H).

^{13}C NMR (75 MHz, Chloroform-*d*) δ 170.6, 161.0, 129.3, 127.5, 113.9, 67.1, 55.5, 44.7.

Spectroscopic data are in accordance with literature.[S19]

(4-Methoxyphenyl)-(4-phenylpiperidin-1-yl)methanone (19B)

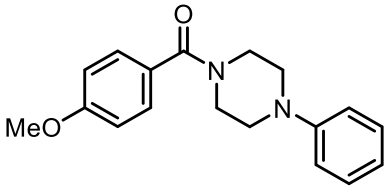

The crude was purified by flash chromatography on silica (DCM/MeOH 99:1) to afford the product as a yellow oil (77.2 mg, 87% yield after isolation).

^{1}H NMR (300 MHz, Chloroform-*d)* δ 7.37 (d, *J* = 8.7 Hz, 2H), 7.25–7.21 (m, 2H), 6.90–6.83 (m, 5H), 3.79–3.73 (m, 7H), 3.14 (bs, 4H).

^{13}C NMR (75 MHz, Chloroform-*d*) δ 170.5, 161.0, 151.1, 129.4, 129.3, 127.8, 120.7, 116.8, 113.9, 55.5, 49.9, 43.3.

HRMS (ESI) m/z: $[M + H]^+$ Calcd for $C_{18}H_{21}N_2O_2^+$ 297.1603; Found 297.1598.

(4-Methoxyphenyl)-(4-phenylpiperidin-1-yl)methanone (20B)

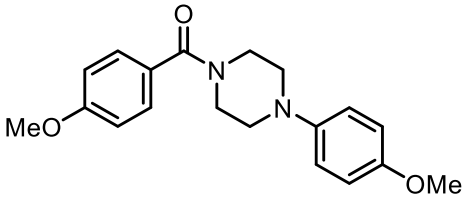

The crude was purified by flash chromatography on silica (DCM/MeOH 99:1) to afford the product as a yellow oil (83.5 mg, 85% yield after isolation).

^{1}H NMR (300 MHz, Chloroform-*d*) δ 7.41 (d, *J* = 8.7 Hz, 2H), 7.03–6.71 (m, 6H), 3.98–3.65 (m, 10H), 3.06 (bs, 4H).

^{13}C NMR (75 MHz, Chloroform-*d*) δ 170.5, 161.0, 154.5, 145.4, 129.3, 127.9, 119.0, 114.6, 113.9, 55.7, 55.5, 51.3, 47.3, 43.1.

HRMS (ESI) m/z: $[M + H]^+$ Calcd for $C_{19}H_{23}N_2O_3^+$ 327.1709; Found 327.1698.

(4-(Benzo[d][1,3]dioxol-5-ylmethyl)piperazin-1-yl)(4-methoxyphenyl)methanone (21B)

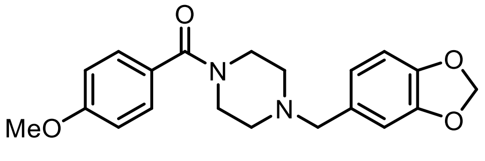

The crude was purified by flash chromatography on silica (DCM/MeOH 99:1) to afford the product as a pale yellow oil (84.2 mg, 79% yield after isolation).

^{1}H NMR (300 MHz, Chloroform-*d*) δ 7.37 (d, *J* = 8.7 Hz, 2H), 6.95–6.81 (m, 3H), 6.73 (s, 2H), 5.94 (s, 2H), 3.82 (s, 3H), 3.65 (bs, 4H), 3.43 (s, 2H), 2.43 (bs, 4H).

^{13}C NMR (75 MHz, $CDCl_3$) δ 170.4, 160.8, 147.8, 146.9, 131.6, 129.2, 128.0, 122.3, 113.8, 109.5, 108.0, 101.1, 62.5, 55.5, 53.1, 47.5, 41.9.

HRMS (ESI) m/z: $[M + H]^+$ Calcd for $C_{20}H_{23}N_2O_4^+$ 355.1658; Found 355.1650.

***N*-benzyl-4-methoxy-N-methylbenzamide (22B)**

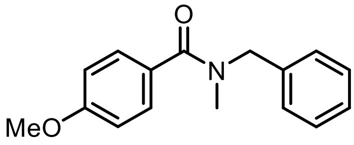

The crude was purified by flash chromatography on silica (Hex/EtOAc 3:2) to afford the product as a colorless oil (60.7 mg, 79% yield after isolation).

^{1}H-NMR (300 MHz, Chloroform-d) δ 7.50–7.17 (m, 7H), 6.89 (d, *J* = 8.1 Hz, 2H), 4.66 (bs, 2H), 3.82 (s, 3H), 2.97 (bs, 3H).

^{13}C-NMR (75 MHz, Chloroform-d) δ 160.8, 137.2, 129.1, 128.9, 128.5, 128.2, 128.2, 127.6, 113.8, 55.5, 51.2, 36.6, 33.8.

Spectroscopic data are in accordance with literature.[S20]

***N*-benzyl-4-methoxy-N-methylbenzamide (23B)**

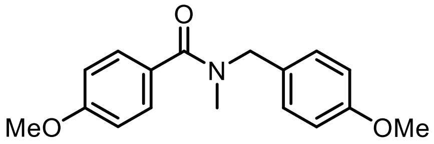

The crude was purified by flash chromatography on silica (Hex/EtOAc 3:2) to afford the product as a colorless oil (70.0 mg, 82% yield after isolation).

^{1}H NMR (300 MHz, Chloroform-*d*) δ 7.42 (d, *J* = 8.7 Hz, 2H), 7.17 (bs, 2H), 6.88 (d, *J* = 8.6 Hz, 4H), 4.58 (bs, 2H), 3.81 (s, 3H), 3.81 (s, 3H), 2.93 (bs, 3H).

^{13}C-NMR (75 MHz, Chloroform-*d*) δ 160.8, 159.2, 129.4, 129.1, 128.7, 128.6, 128.4, 114.3, 113.8, 55.4, 50.6, 37.0, 33.9.

Spectroscopic data are in accordance with literature.[S21]

^{1}H NMR (300 MHz, Chloroform-*d*) δ 7.17 (s, 0H), 6.89 (d, *J* = 8.6 Hz, 2H), 4.58 (s, 2H), 3.81 (d, *J* = 2.7 Hz, 5H), 2.93 (s, 2H).

***N*-(3,4-Dimethoxyphenethyl)-4-methoxybenzamide (24B)**

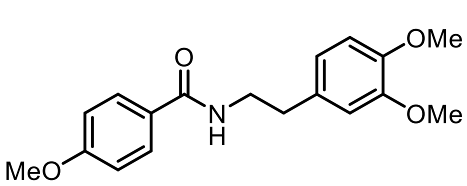

The crude was purified by flash chromatography on silica (DCM/MeOH 98:2) to afford the product as a pale yellow oil (77.7 mg, 82% yield after isolation).

^{1}H NMR (300 MHz, Chloroform-*d*) δ 7.66 (d, *J* = 8.8 Hz, 2H), 6.89 (d, *J* = 8.8 Hz, 2H), 6.87–6.71 (m, 3H), 6.09 (bs, 1H), 3.86 (s, 3H), 3.83 (s, 6H), 3.67 (q, *J* = 6.8 Hz, 2H), 2.86 (t, *J* = 6.9 Hz, 2H).

^{13}C NMR (75 MHz, , Chloroform-*d*) δ 167.1, 162.3, 149.2, 147.9, 131.7, 128.7, 127.0, 120.8, 113.9, 112.2, 111.6, 56.1, 56.0, 55.5, 41.3, 35.4.

Spectroscopic data are in accordance with literature.[S22]

Methyl (4-methoxybenzoyl)-L-valinate (25B)

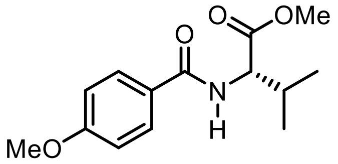

4 equiv. of K_2CO_3 were employed (instead of 1 equiv) to liberate the free the methyl valinate amine from the correspondng chlorhydrate. The crude was purified by flash chromatography on silica (Hex/EtOAc 7:3) to afford the product as a pale yellow oil (51.2 mg, 64% yield after isolation).

^{1}H NMR (300 MHz, Chloroform-*d*) δ 7.78 (d, *J* = 8.8 Hz, 2H), 6.94 (d, *J* = 8.8 Hz, 2H), 6.53 (d, *J* = 8.6 Hz, 1H), 4.77 (dd, *J* = 8.6, 4.9 Hz, 1H), 3.85 (s, 3H), 3.77 (s, 3H), 2.38 (pd, *J* = 6.9, 4.9 Hz, 1H), 0.99 (t, *J* = 6.6 Hz, 6H).

^{13}C NMR (75 MHz, Chloroform-*d*) δ 173.0, 166.9, 162.6, 129.0, 126.6, 114.0, 57.5, 55.6, 52.4, 31.8, 19.1, 18.1.

Spectroscopic data are in accordance with literature.[S23]

2. References

(S1) Ponath, S.; Menger, M.; Grothues, L.; Webwe, M.; Lentz, D.; Strohmann, C.; Christmann, M. Mechanistic Studies on the Organocatalytic α-Chlorination of Aldehydes: The Role and Nature of Off-Cycle Intermediates. *Angew. Chem., Int. Ed.* **2018**, *57*, 11683−11687.

(S2) Nouaille, A.; Terzani, F.; Fakih, Y.; Hannedouche, J.; Magnier, E.; Gosmini, C.; Dagousset G. Metal-Free Multicatalytic Decarbonylation of Aldehydes Driven by Light. *Angew. Chem. Int. Ed.* **2025***, 64*, e202424459.

(S3) Cassera, E.; Martini, V.; Abrami, S.; Della Ca', N.; Ravelli, D.; Fagnoni, M.; Capaldo, L. A Decarbonylative Strategy to Enhance Efficiency and Regioselectivity in Photocatalyzed Hydrogen-Atom Transfer. *JACS Au* **2025**, *5*, 3491–3499.

(S4) Capaldo, L.; Merli, D.; Fagnoni, M.; Ravelli, D. Visible Light Uranyl Photocatalysis: Direct C−H to C−C Bond Conversion. *ACS Catal.* **2019**, *9*, 3054−3058.

(S5) Yu, Y.-Q.; Wang, Z.-L. A simple, efficient and green procedure for Knoevenagel condensation in water or under solvent-free conditions. *J. Chin. Chem. Soc.* **2012**, *60*, 288–292.

(S6) Anamika, Yadav, C. L.; Drew, M. G. B.; Kumar, K.; Singh, N. Ferrocene-Functionalized Dithiocarbamate Zinc(II) Complexes as Efficient Bifunctional Catalysts for the One-Pot Synthesis of Chromene and Imidazopyrimidine Derivatives via Knoevenagel Condensation Reaction. *Inorg. Chem.* **2021**, *60*, 6446−6462.

(S7) Gontala, A.; Woo, S. K. Visible-Light Photoredox-Catalyzed α-Regioselective Conjugate Addition of Allyl Groups to Activated Alkenes. *Adv. Synth. Catal.* **2020**, *362*, 3223–3228.

(S8) Fichez, J.; Lapuh, M. I.; Buttard, F.; Besset, T. Selectivity Switch in Non-Directed Palladium-Catalyzed C−H Olefination of Chlorobenzene Derivatives**.** *Adv. Synth. Catal.* **2024**, *366*, 2811−2822.

(S9) Luguera Ruiz, A.; Mariani, E.; Protti, S.; Fagnoni, M. Photoredox catalyzed release of carbon-based radicals from 2-substituted-1,3-imidazolidines. *Org. Chem. Front.* **2024**, *11*, 661–667.

(S10) Kiyokawa, K.; Nagata, T.; Hayakawa, J.; Minakata, S. Straightforward Synthesis of 1,2-Dicyanoalkanes from Nitroalkenes and Silyl Cyanide Mediated by Tetrabutylammonium Fluoride. *Chem.- Eur. J.* **2015**, *21*, 1280−1285.

(S11) Laudadio, G.; Deng, Y.-C.; van der Wal, K.; Ravelli, D.; Nuño, M.; Fagnoni, M.; Guthrie, D.; Sun, Y.-H.; Noël, T. C(sp^3)–H functionalizations of light hydrocarbons using decatungstate photocatalysis in flow. *Science* **2020,** *369,* 92–96.
(S12) Xie, H.; Guo, J.; Wang, Y.-Q.; Wang, K.; Guo, P.; Su, P.-F.; Wang, X., Shu, X.-Z. Radical Dehydroxylative Alkylation of Tertiary Alcohols by Ti Catalysis. *J. Am. Chem. Soc*. **2020**, *142*, 16787−16794.
(S13) Montanaro, S.; Ravelli, D.; Merli, D.; Fagnoni, M.; Albini, A. Decatungstate As Photoredox Catalyst: Benzylation of Electron-Poor Olefins. *Org. Lett.* **2012**, *14*, 4218−4221.
(S14) Kumar, V.; Connon, S. J. Direct, efficient NHC-catalysed aldehyde oxidative amidation: *in situ* formed benzils as unconventional acylating agents. *Chem. Commun*. **2017**, *53*, 10212–10215.
(S15) Tang, Z.; Yao, Z.; Yu, Y.; Huang, J.; Ma, X.; Zhao, X.; Chang, Z.; Zhao, D. Photoredox-Catalyzed [3 + 2] Annulation of Aromatic Amides with Olefins via Iminium Intermediates. *Angew. Chem., Int. Ed.* **2024**, *63*, e202412152.
(S16) Tolba, A. H.; Krupicka, M.; Chudoba, J.; Cibulka, R. Amide Bond Formation via Aerobic Photooxidative Coupling of Aldehydes with Amines Catalyzed by a Riboflavin Derivative. *Org. Lett.* **2021**, *23*, 6825−6830.
(S17) Maji, S.; Roy, M.; Shaikh, K.; Adhikari, D. Organophotocatalytic dehydrogenative preparation of amides directly from alcohol. *Green Chem*. **2023**, *25*, 8019–8025.
(S18) Chniti, S.; Kollár, L.; Bényei, A.; Takács, A. Highly Selective Synthesis of 6-Glyoxylamidoquinoline Derivatives via Palladium-Catalyzed Aminocarbonylation. *Molecules* **2022**, *27*, 4.
(S19) Kumar, R.; Meher, R. K., Karmakar, H.; Panda, T. K. Hydrosilylation of nitriles and tertiary amides using a zinc precursor. *Org. Biomol. Chem*. **2024**, *22*, 3053–3058.
(S20) Vyas, V.; Kumar V.; Indra, A. The low loading of metal in metal–organic framework-derived NiNx@NC promotes amide formation through C–N coupling. *Chem. Commun*. **2024**, *60*, 2544–2547.
(S21) Jia, J. W., Kato, T.; Maruoka, K. *p*-Methoxybenzyl-Radical-Promoted Chemoselective Protection of *sec*-Alkylamides. *J. Org. Chem.* **2023**, *88*, 2575–2582.
(S22) Chaitanya, M.; Yadagiri, D.; Anbarasan, P. Rhodium catalyzed cyanation of chelation assisted C−H bonds. *Org. Lett*. **2013**,*15*, 4960−4963.

(S23) Čarný, T.; Kisszékelyi, P.; Markovič, M.; Gracza, T.; Koóš, P.; Šebesta, R. Mechanochemical Pd-Catalyzed Amino-and Oxycarbonylations using $FeBr_2(CO)_4$ as a CO Source. *Org. Lett.* **2023**, 25, 8617–8621.

3. NMR spectra

^{1}H NMR (300 MHz, Chloroform-d)

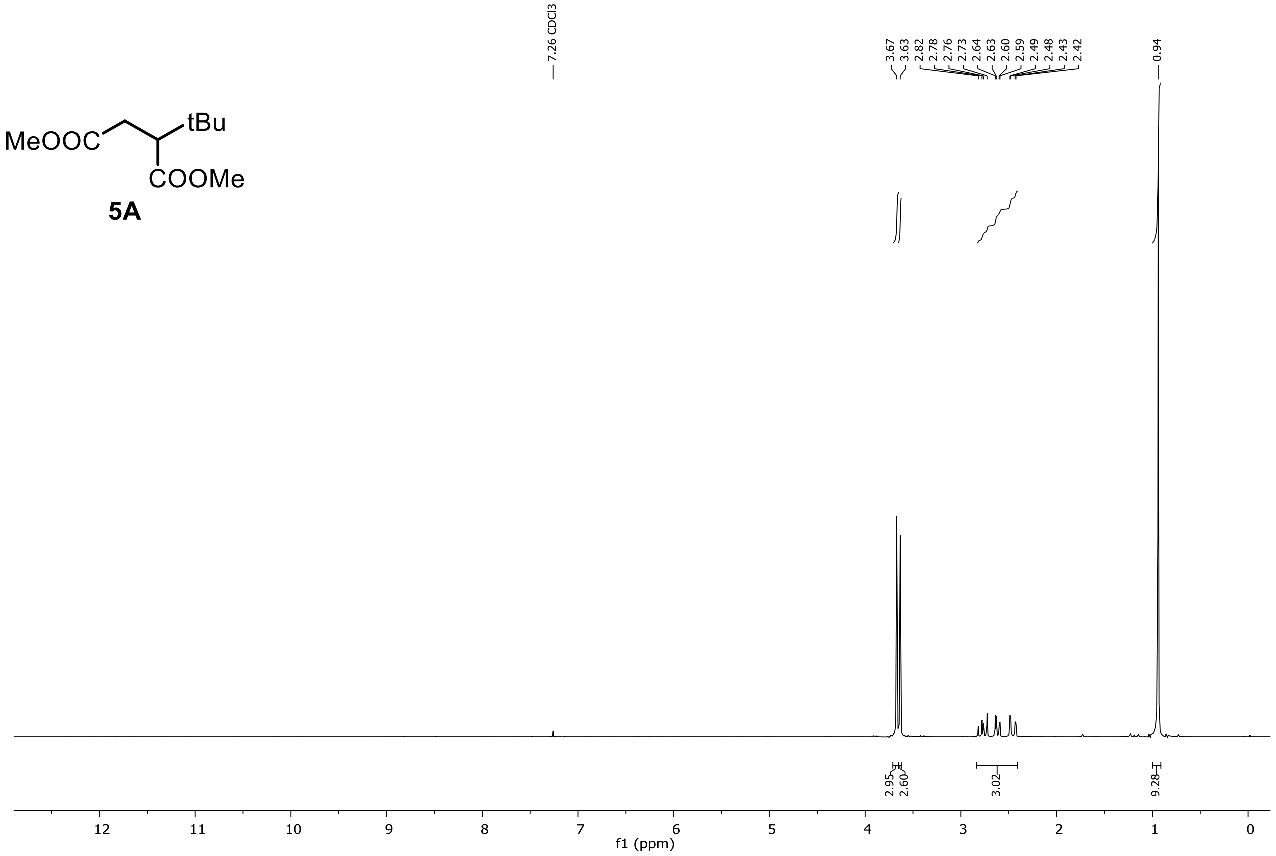

^{13}C NMR (75 MHz, Chloroform-d)

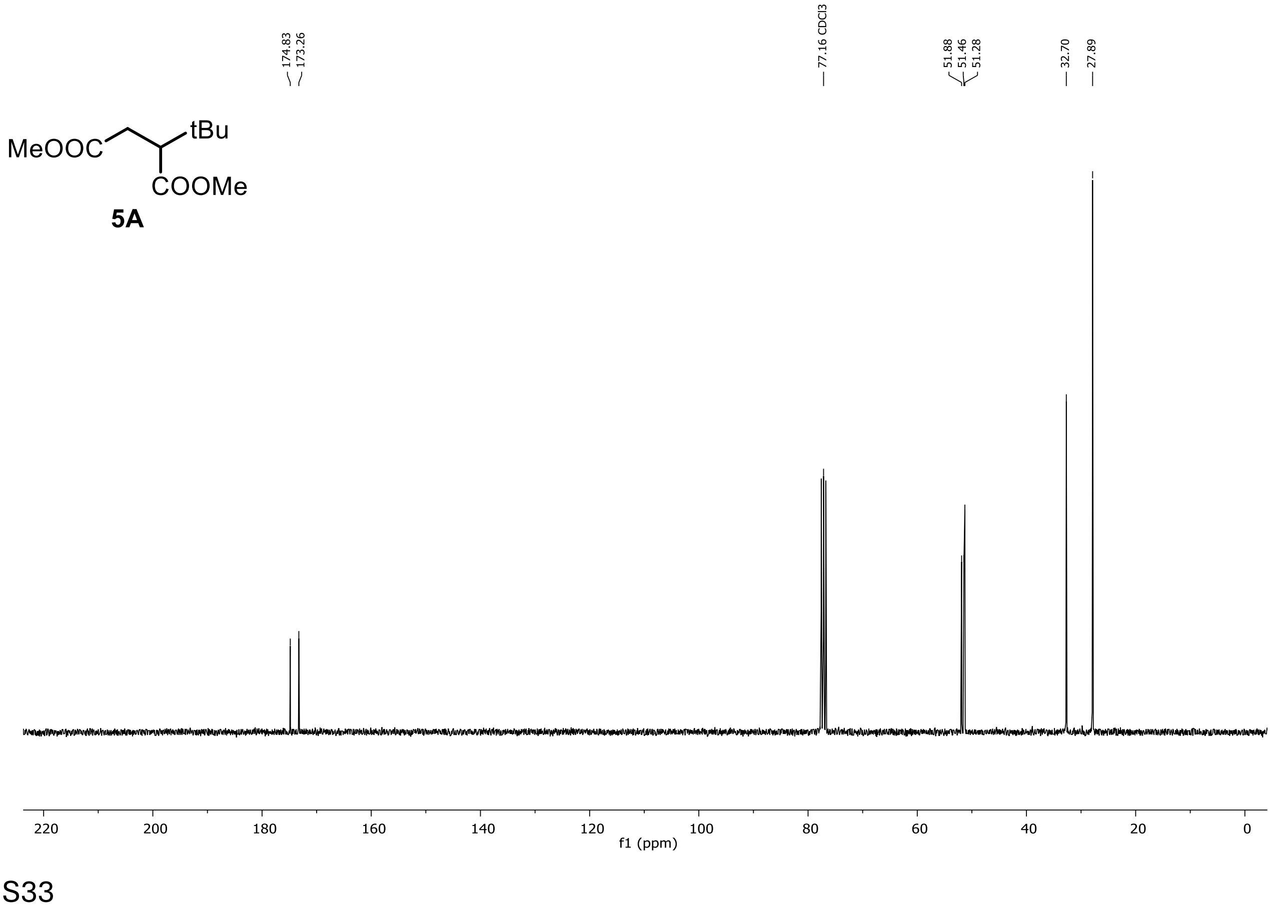

^{1}H NMR (300 MHz, Chloroform-d)

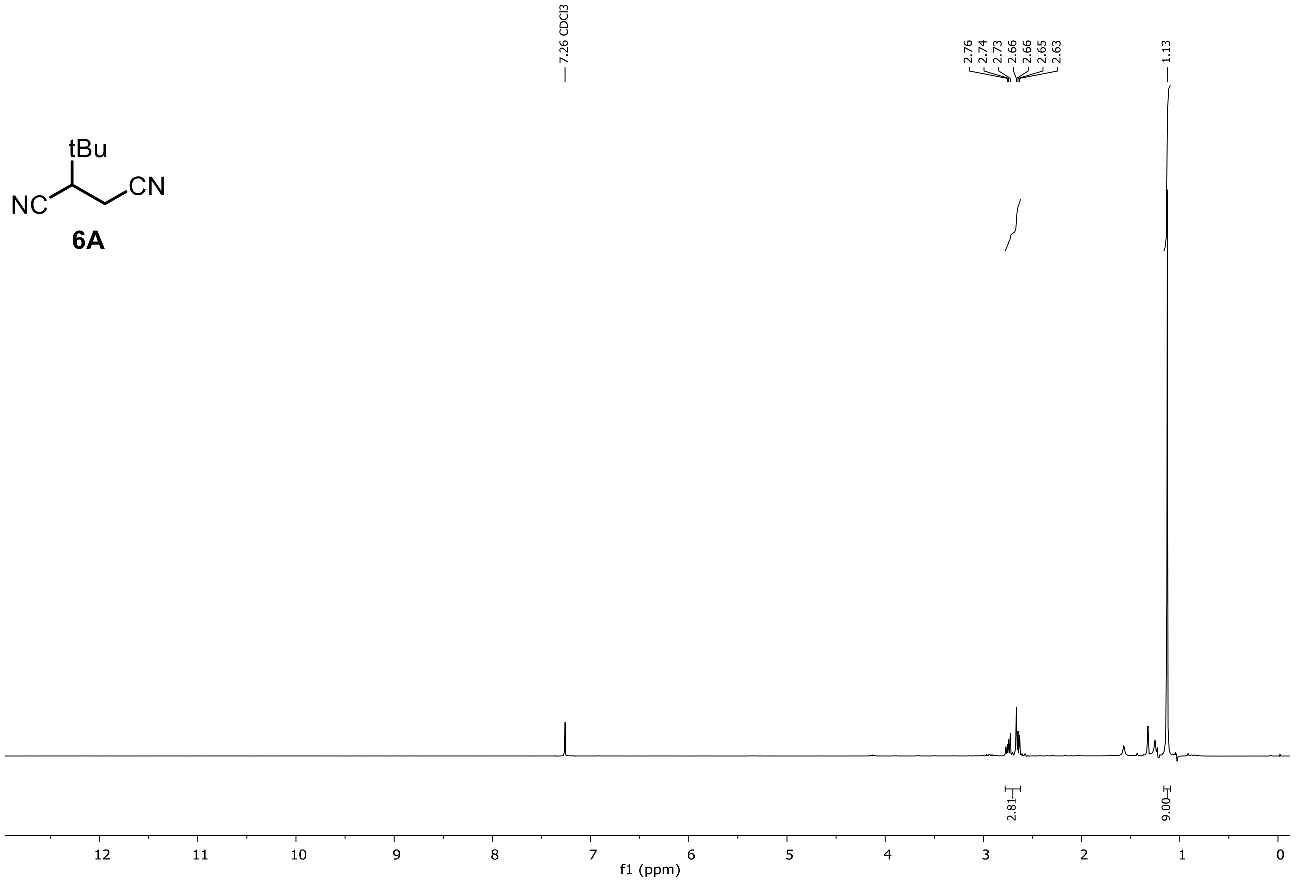

^{13}C NMR (75 MHz, Chloroform-d)

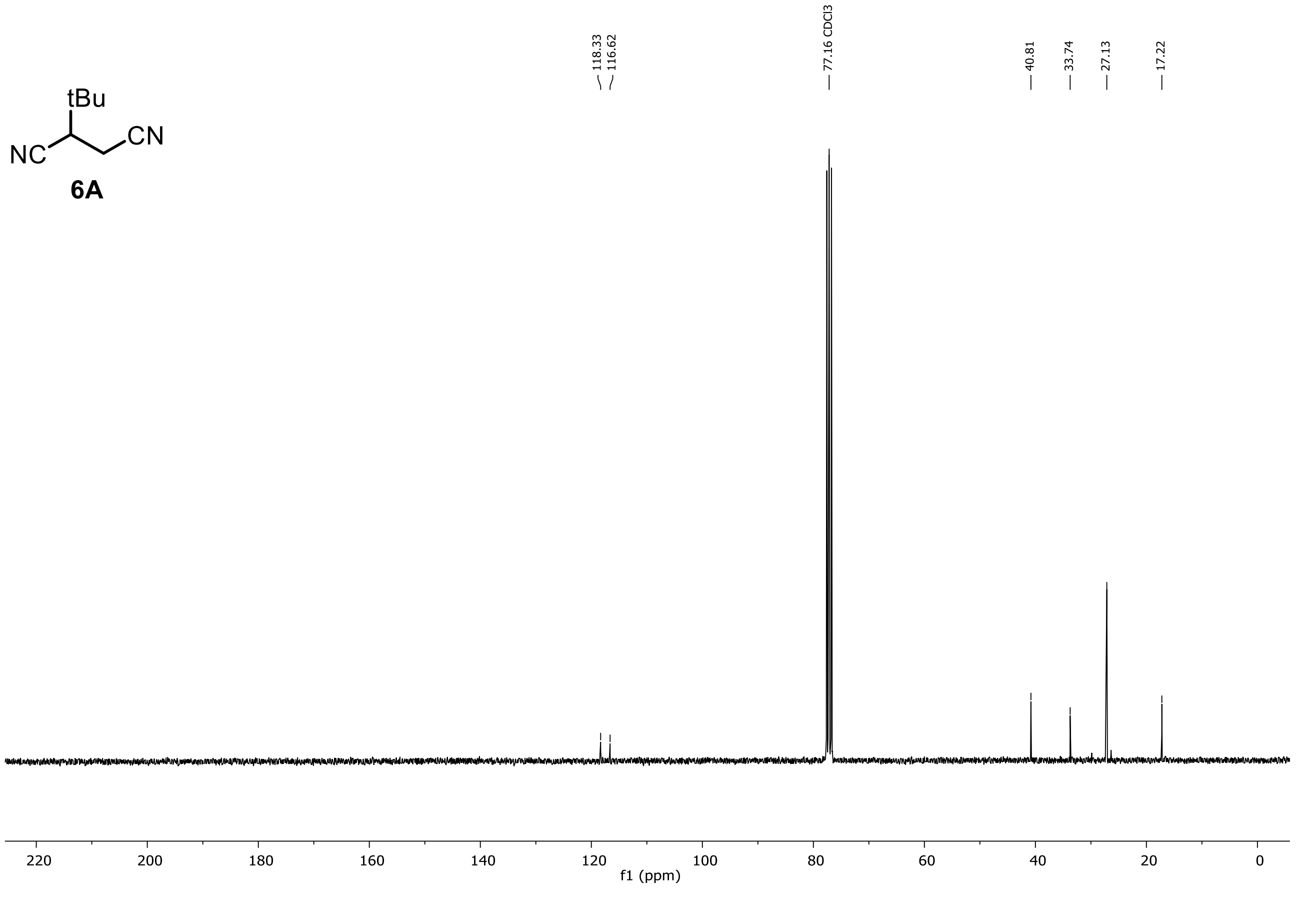

^{1}H NMR (300 MHz, Chloroform-d)

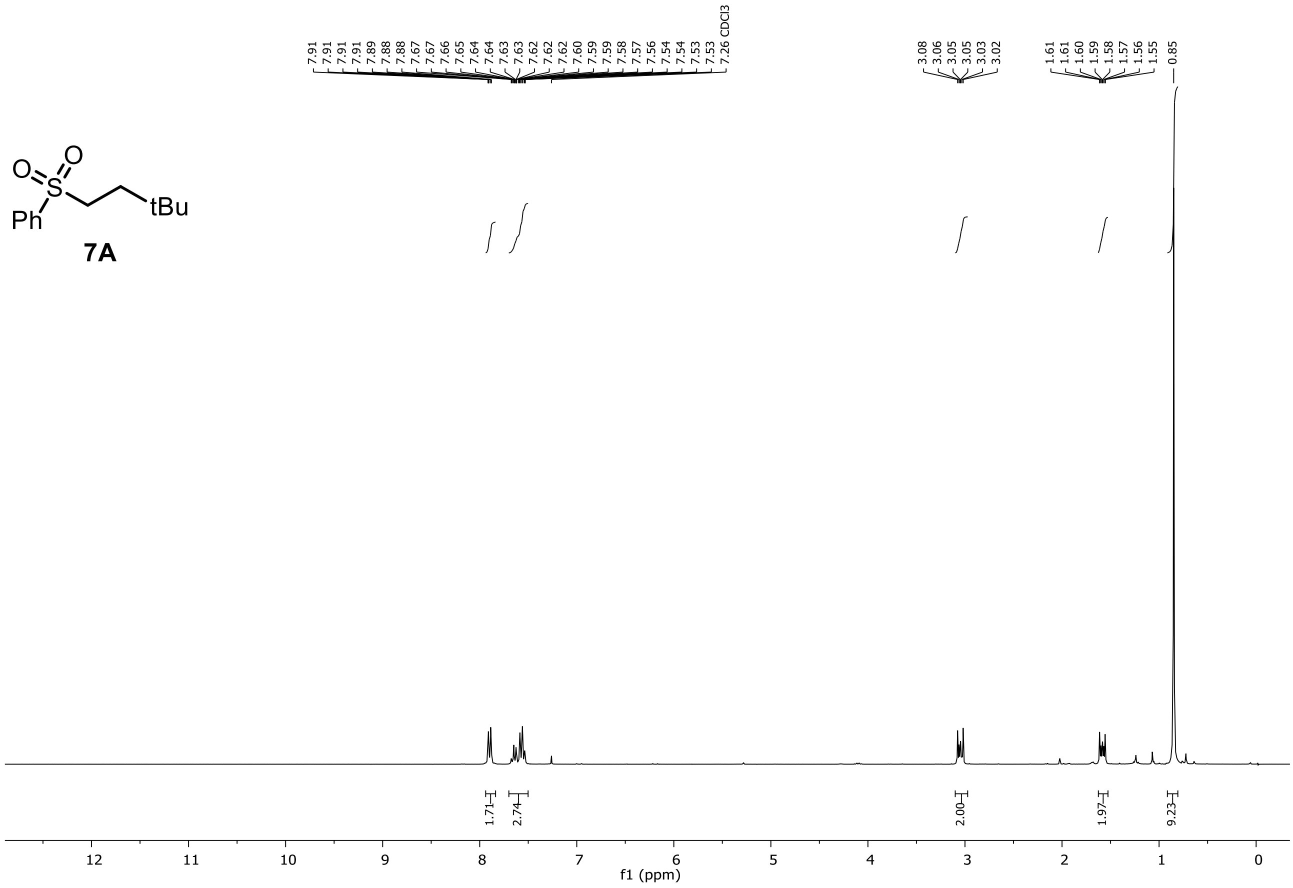

^{13}C NMR (75 MHz, Chloroform-d)

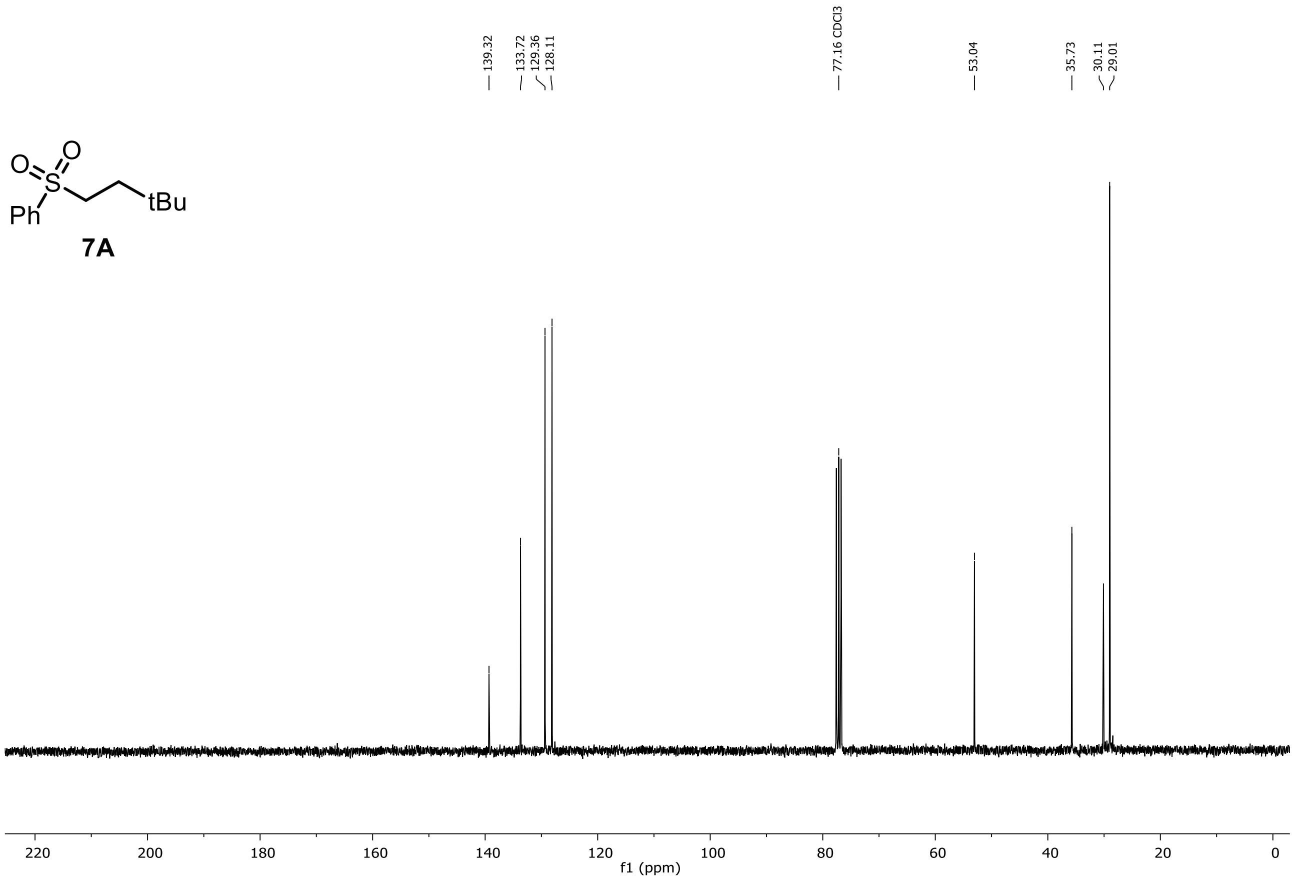

^{1}H NMR (300 MHz, Chloroform-d)

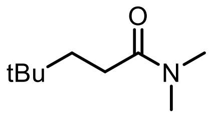

8A

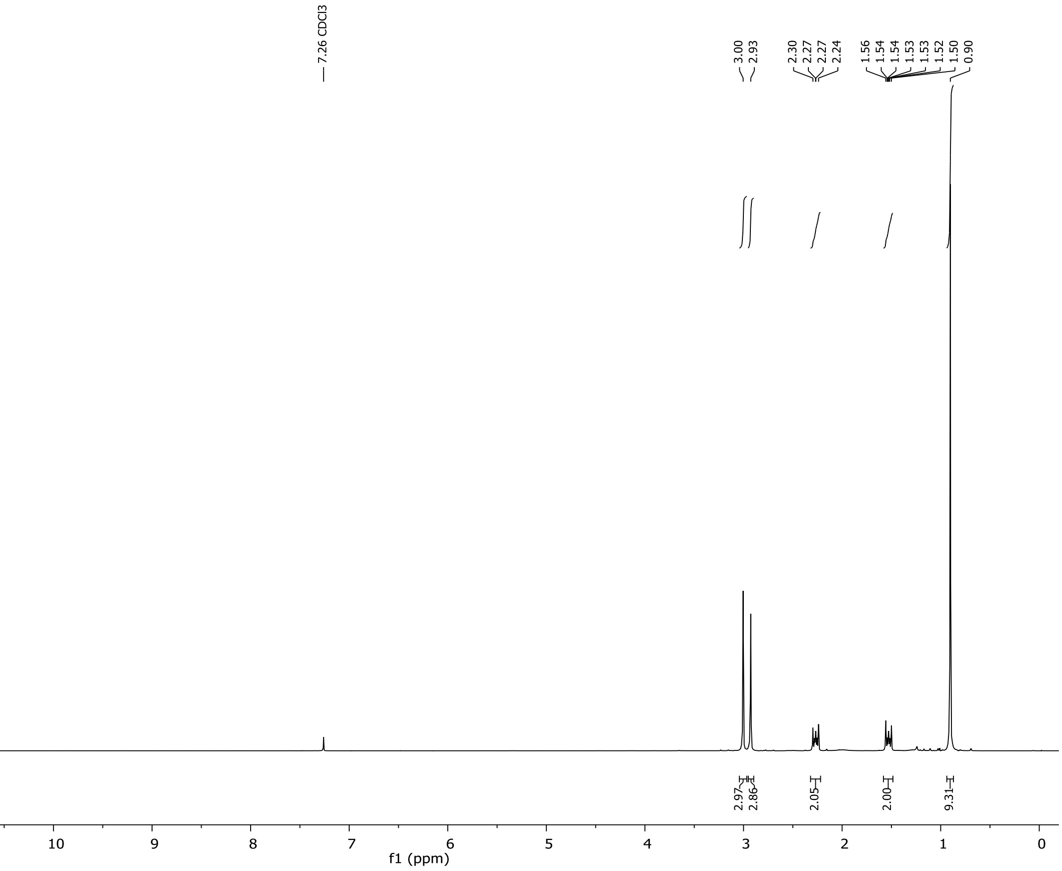

^{13}C NMR (75 MHz, Chloroform-d)

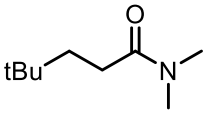

8A

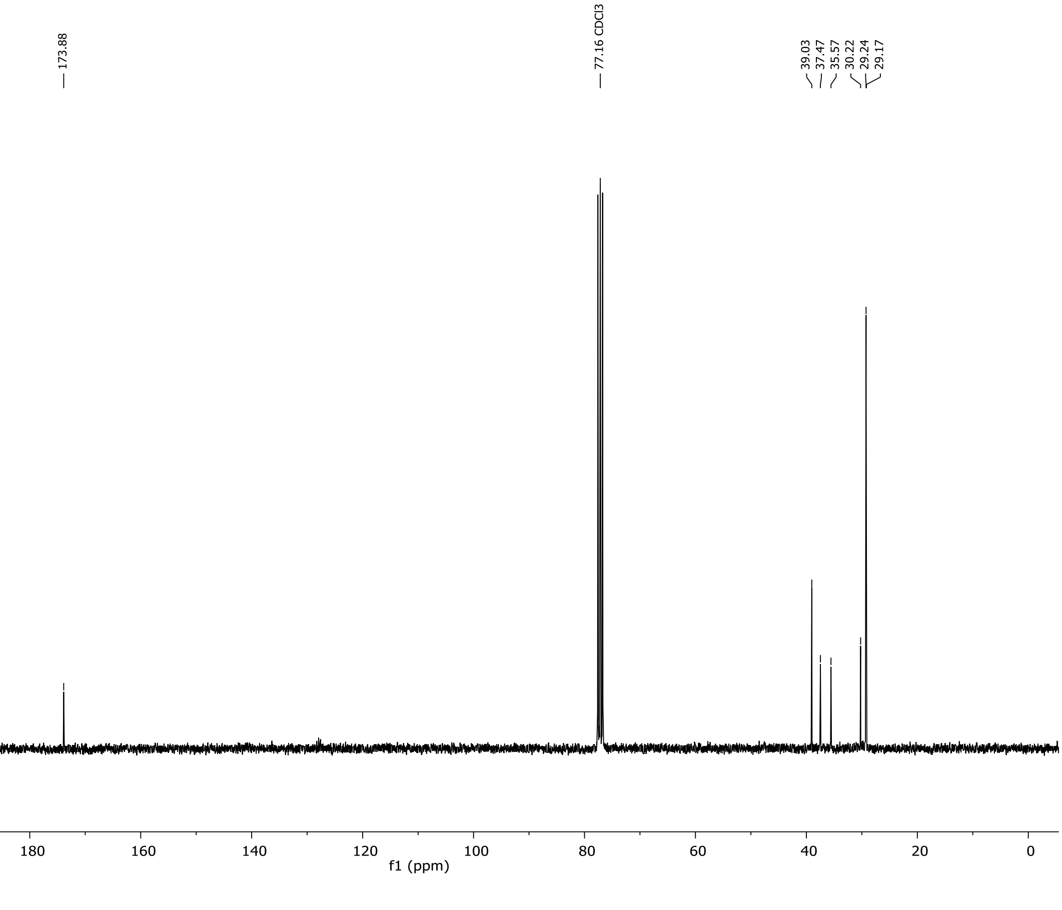

^{1}H NMR (300 MHz, Chloroform-d)

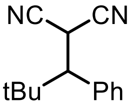

9A

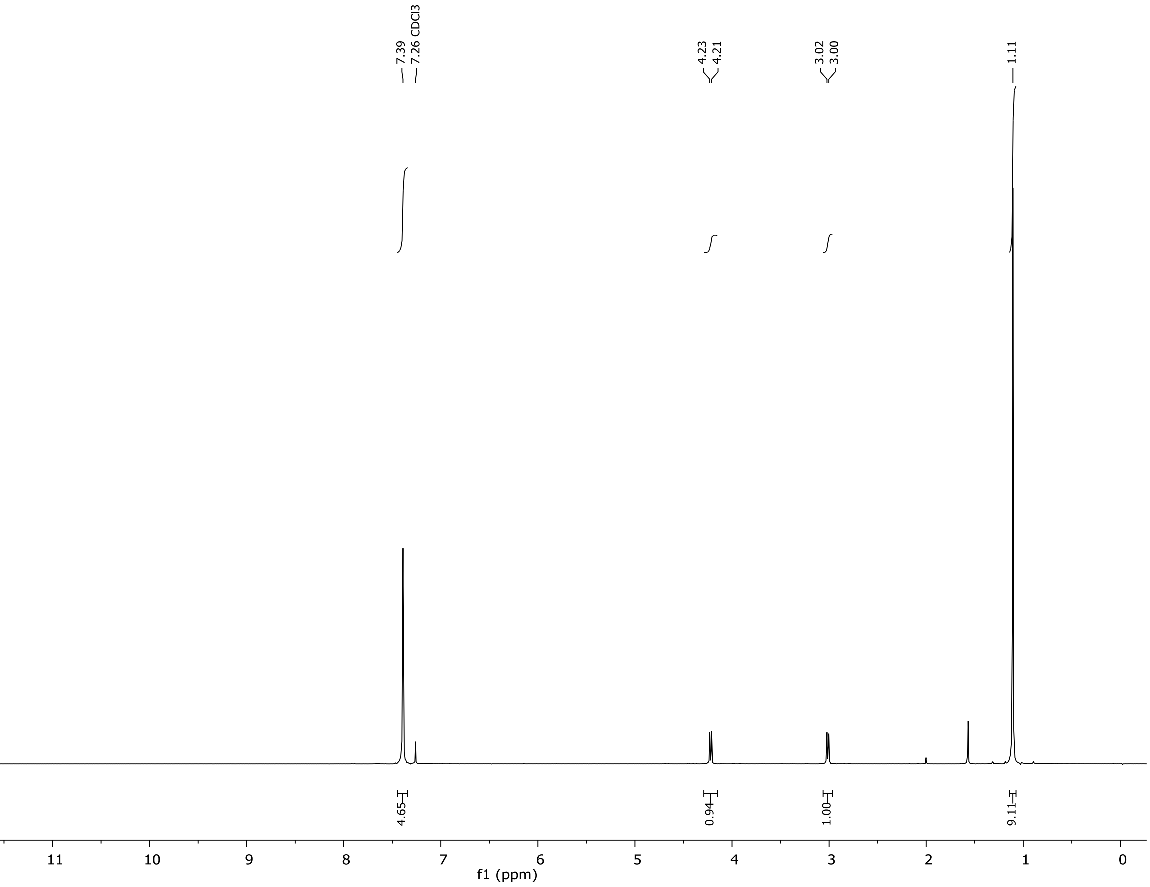

^{13}C NMR (75 MHz, Chloroform-d)

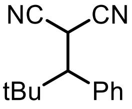

9A

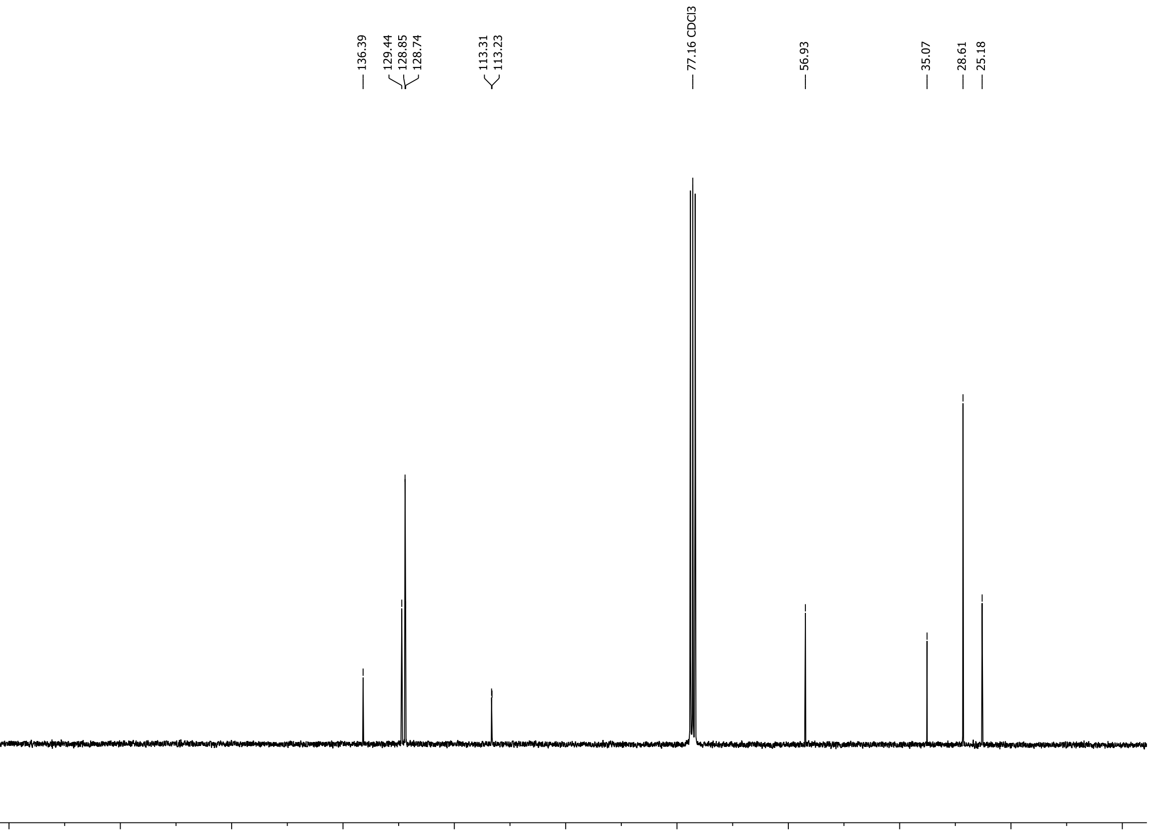

^{1}H NMR (300 MHz, Chloroform-d)

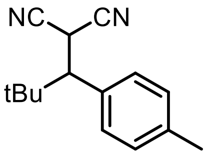

10A

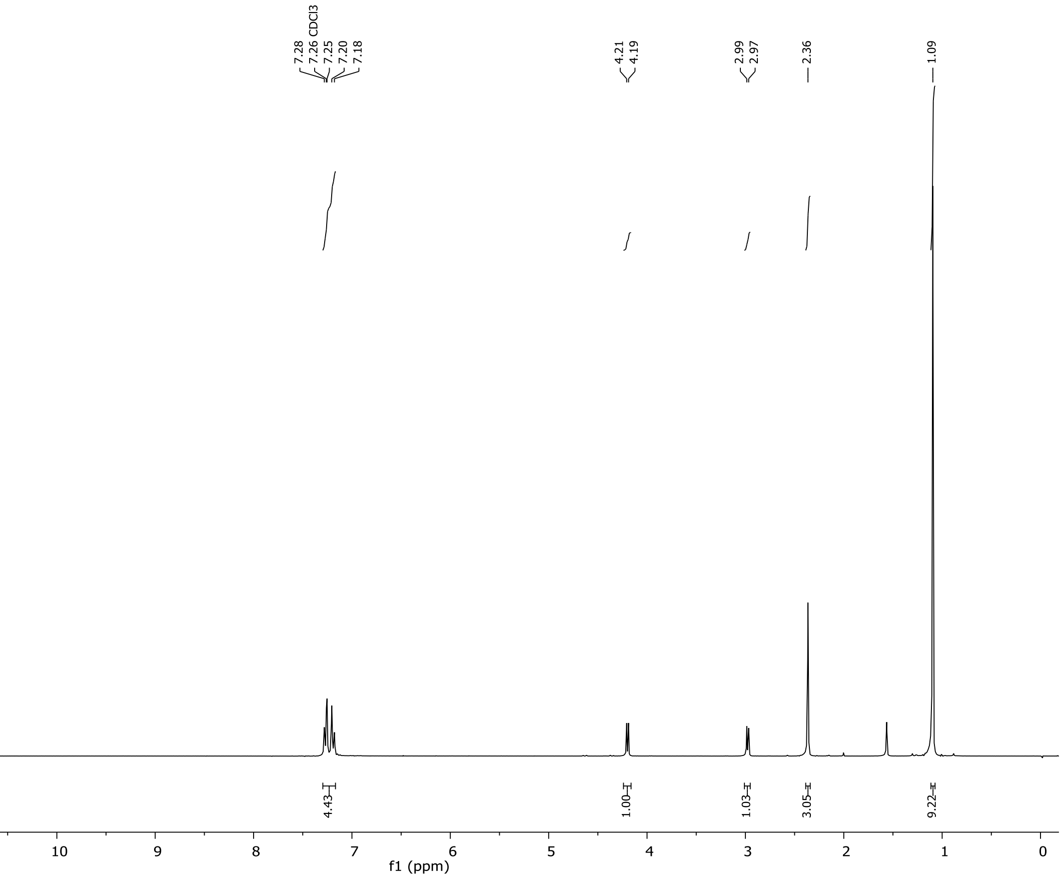

^{13}C NMR (75 MHz, Chloroform-d)

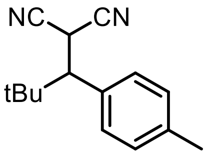

10A

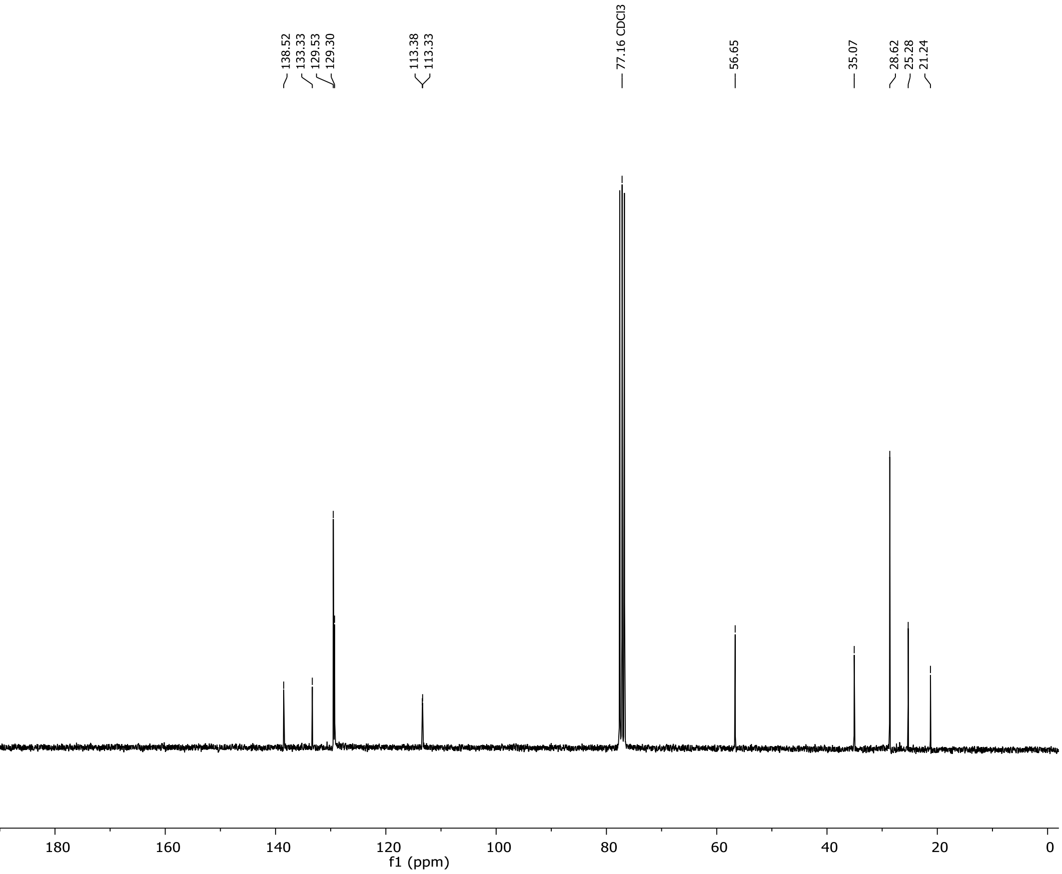

^{1}H NMR (300 MHz, Chloroform-d)

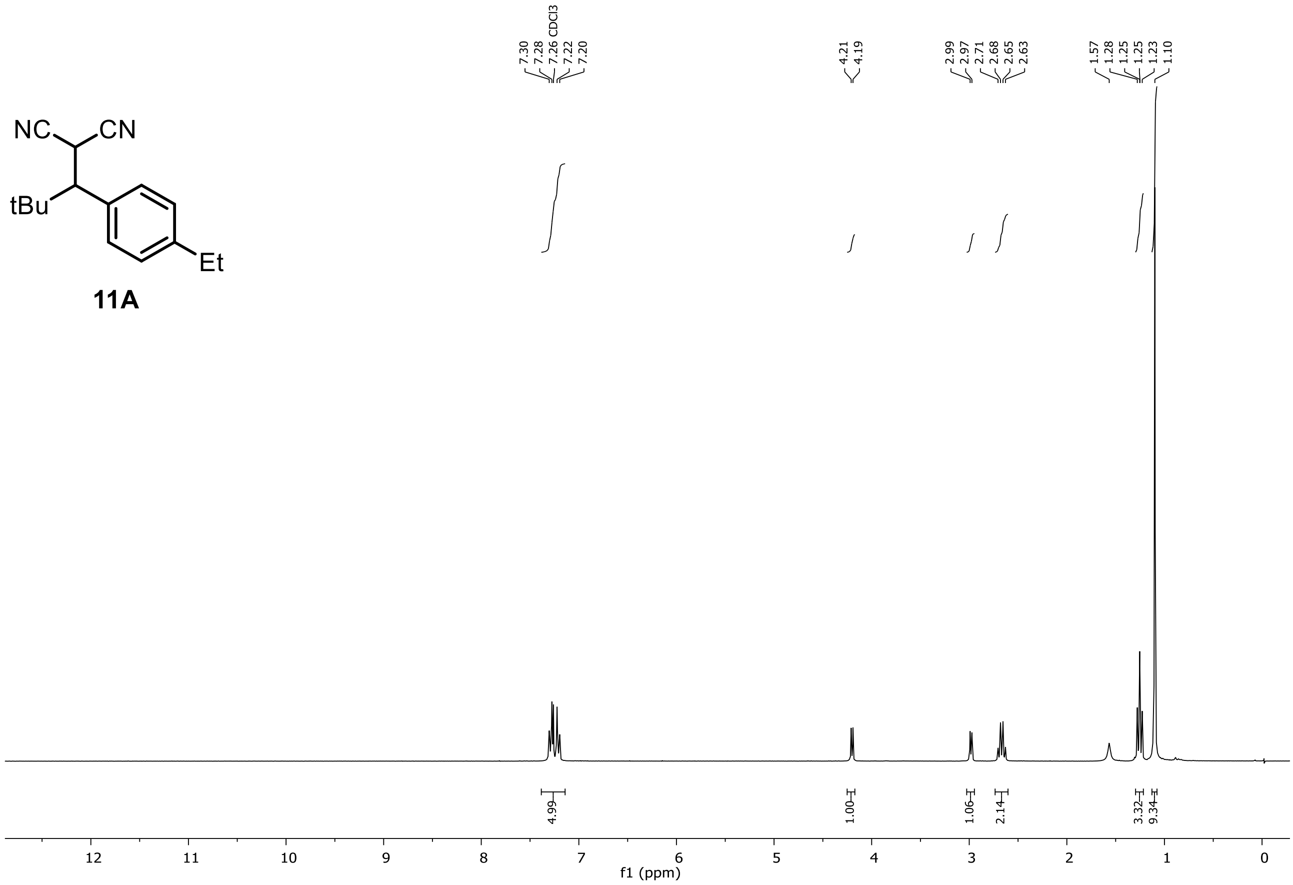

^{13}C NMR (75 MHz, Chloroform-d)

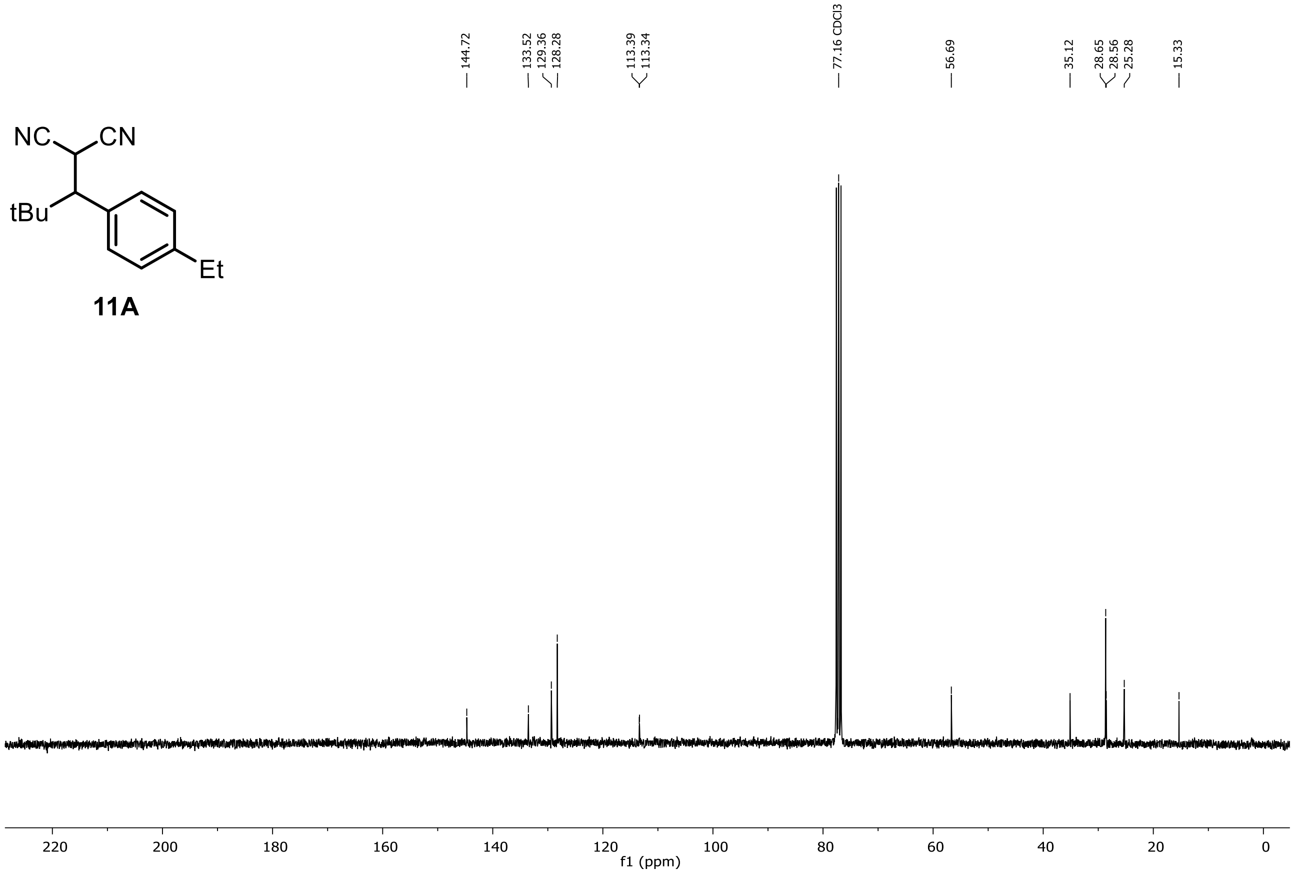

^{1}H NMR (300 MHz, Chloroform-d)

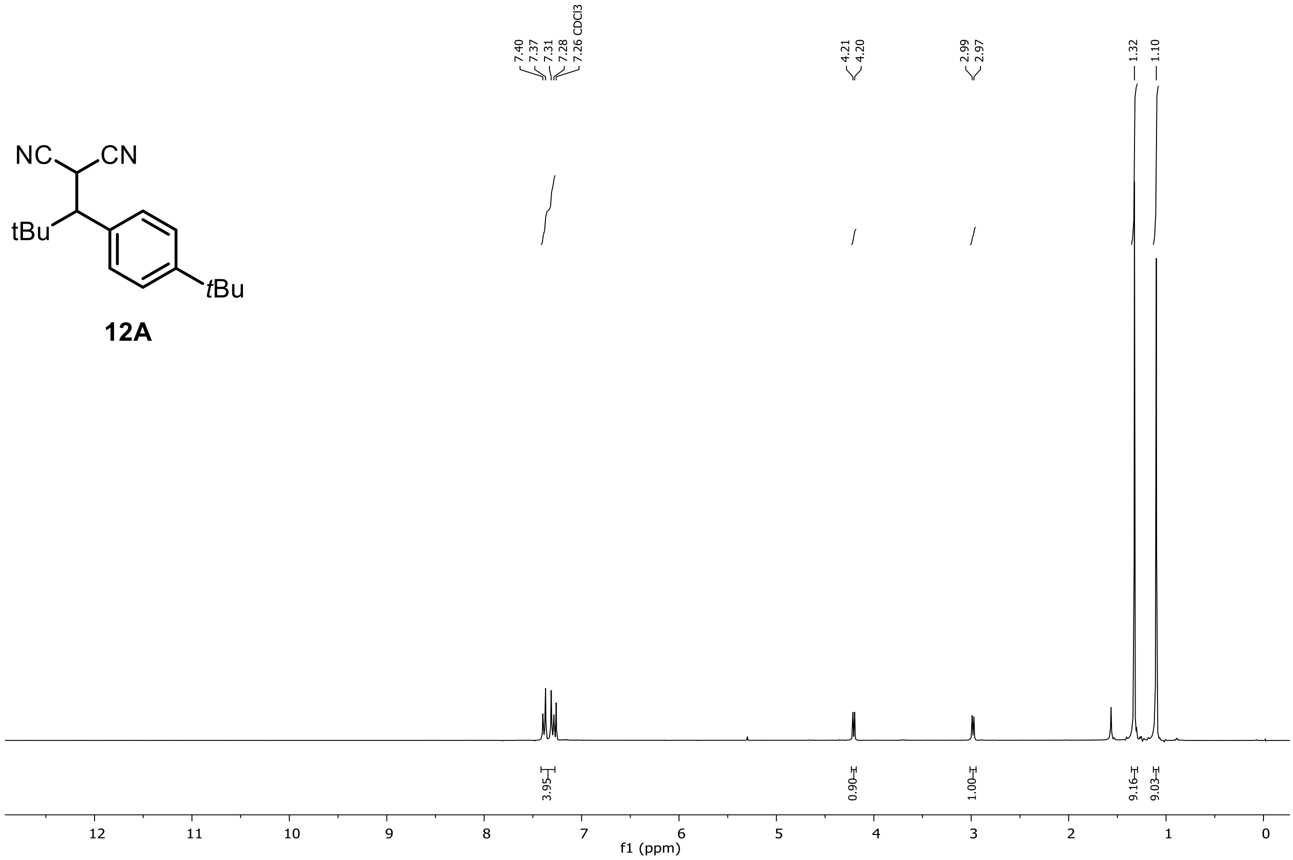

^{13}C NMR (75 MHz, Chloroform-d)

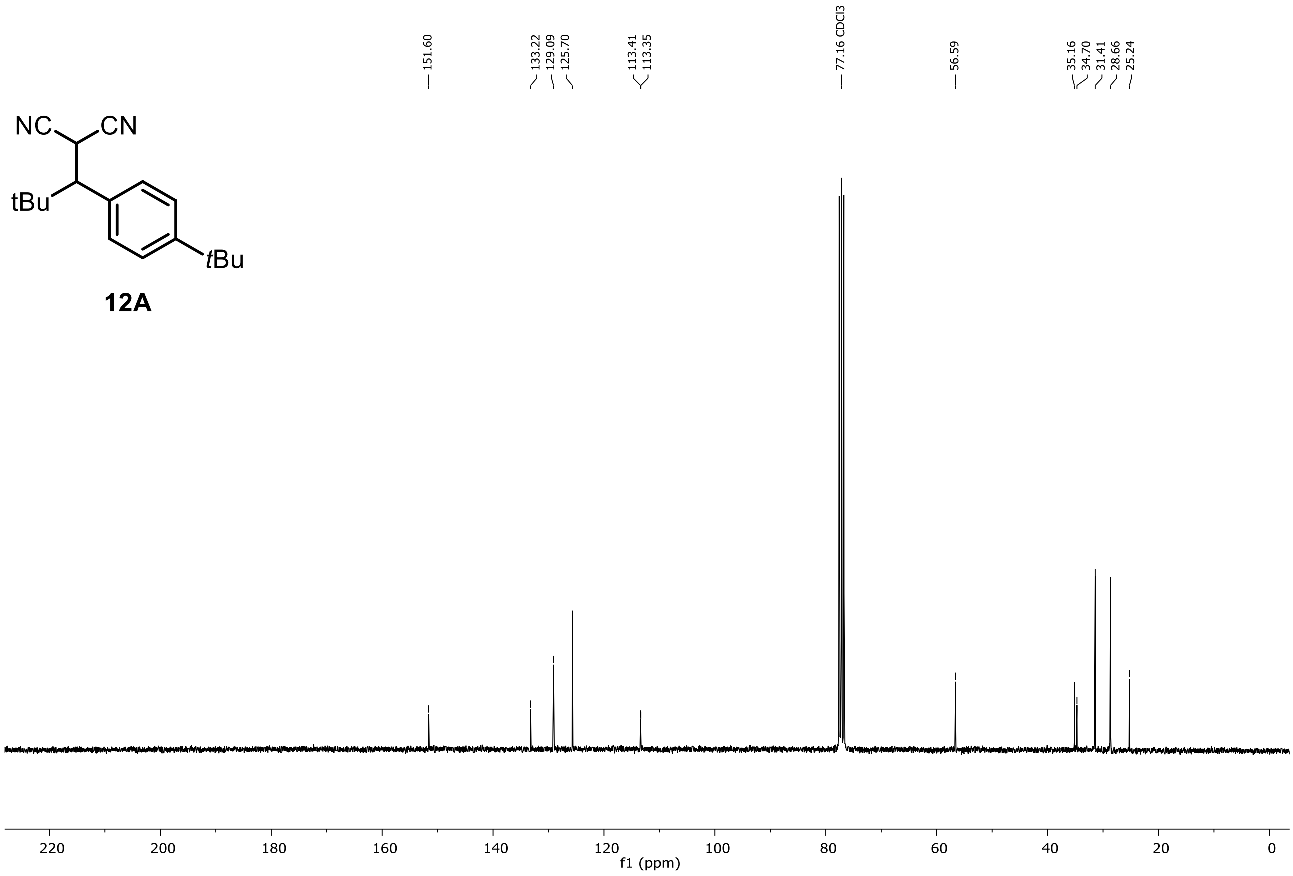

^{1}H NMR (300 MHz, Chloroform-d)

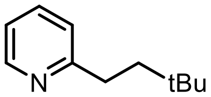

13A

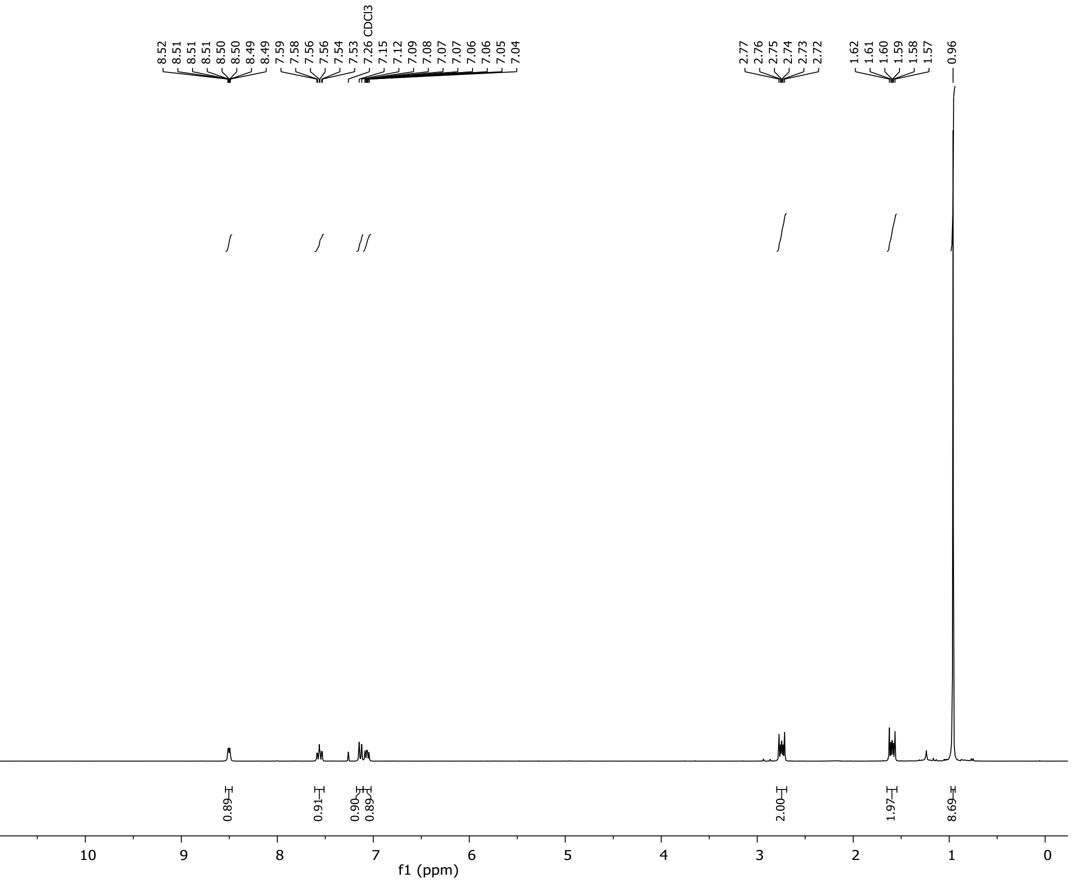

^{13}C NMR (75 MHz, Chloroform-d)

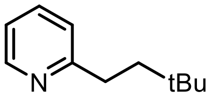

13A

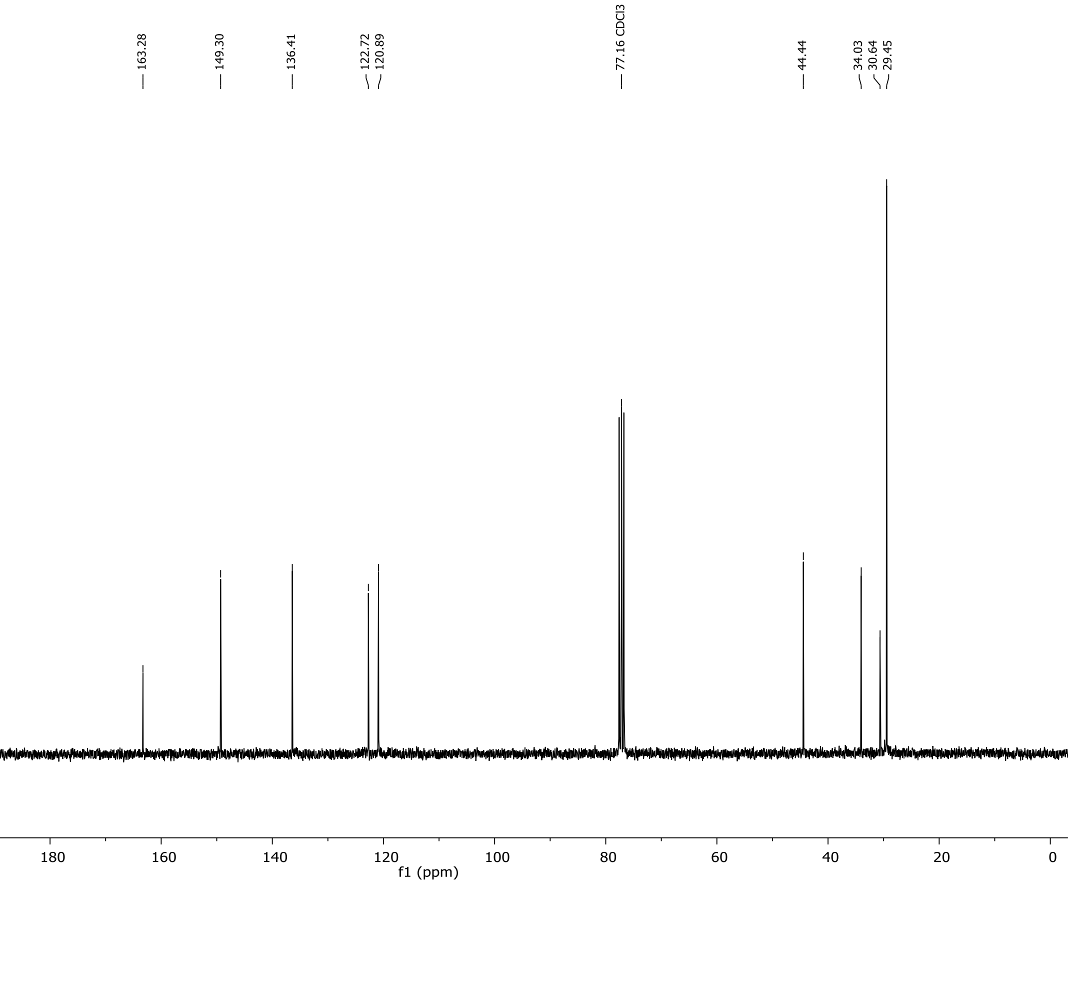

^{1}H NMR (300 MHz, Chloroform-d)

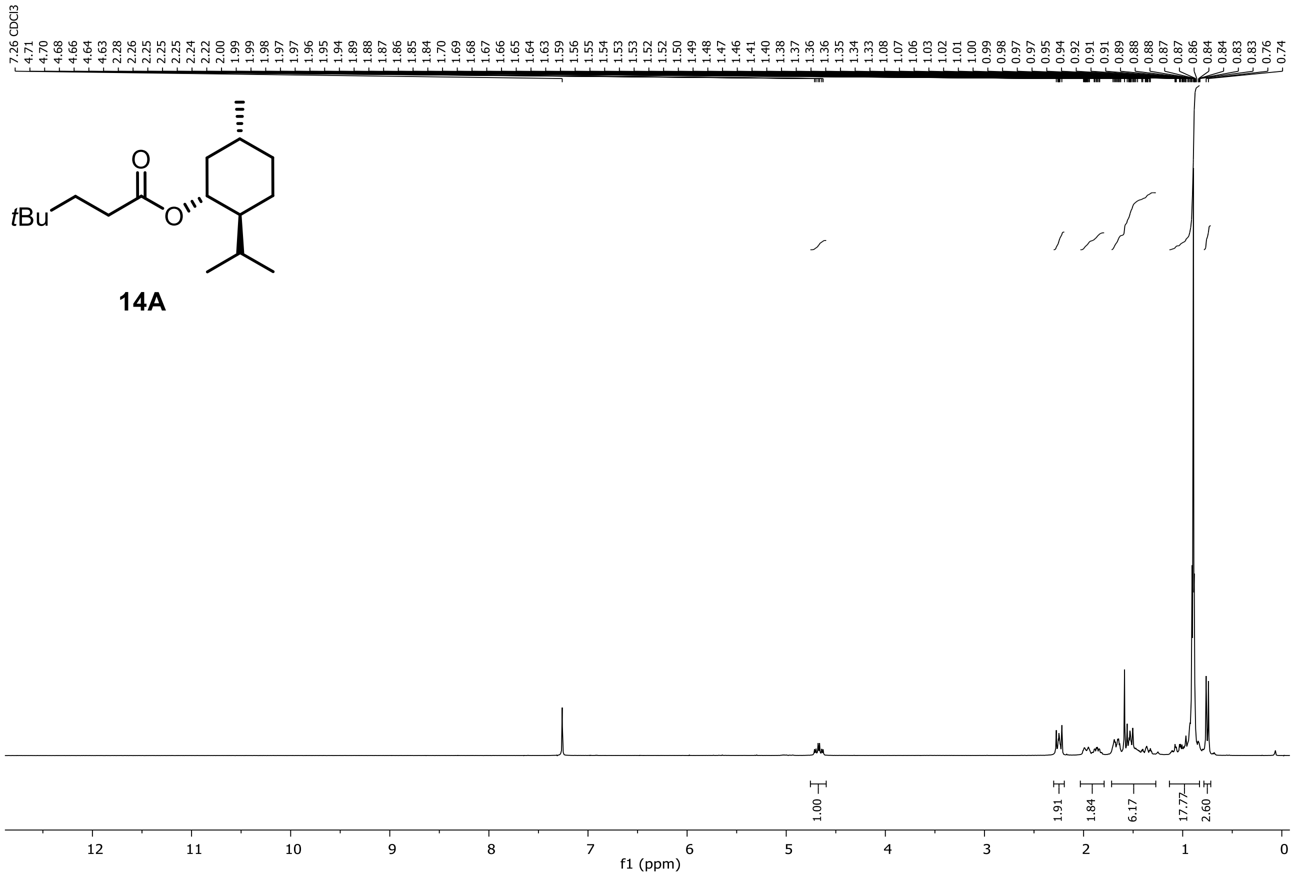

^{13}C NMR (75 MHz, Chloroform-d)

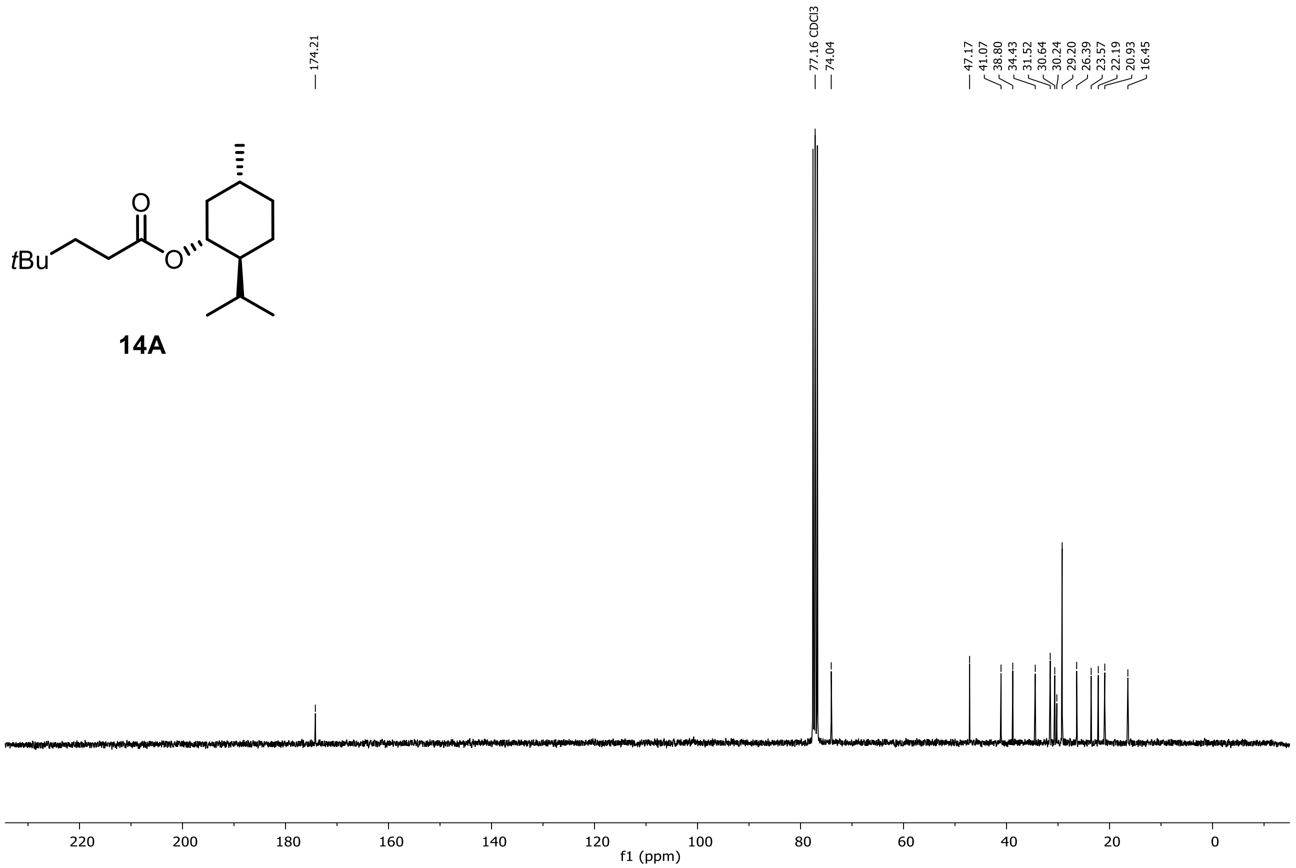

^{1}H NMR (300 MHz, Chloroform-d)

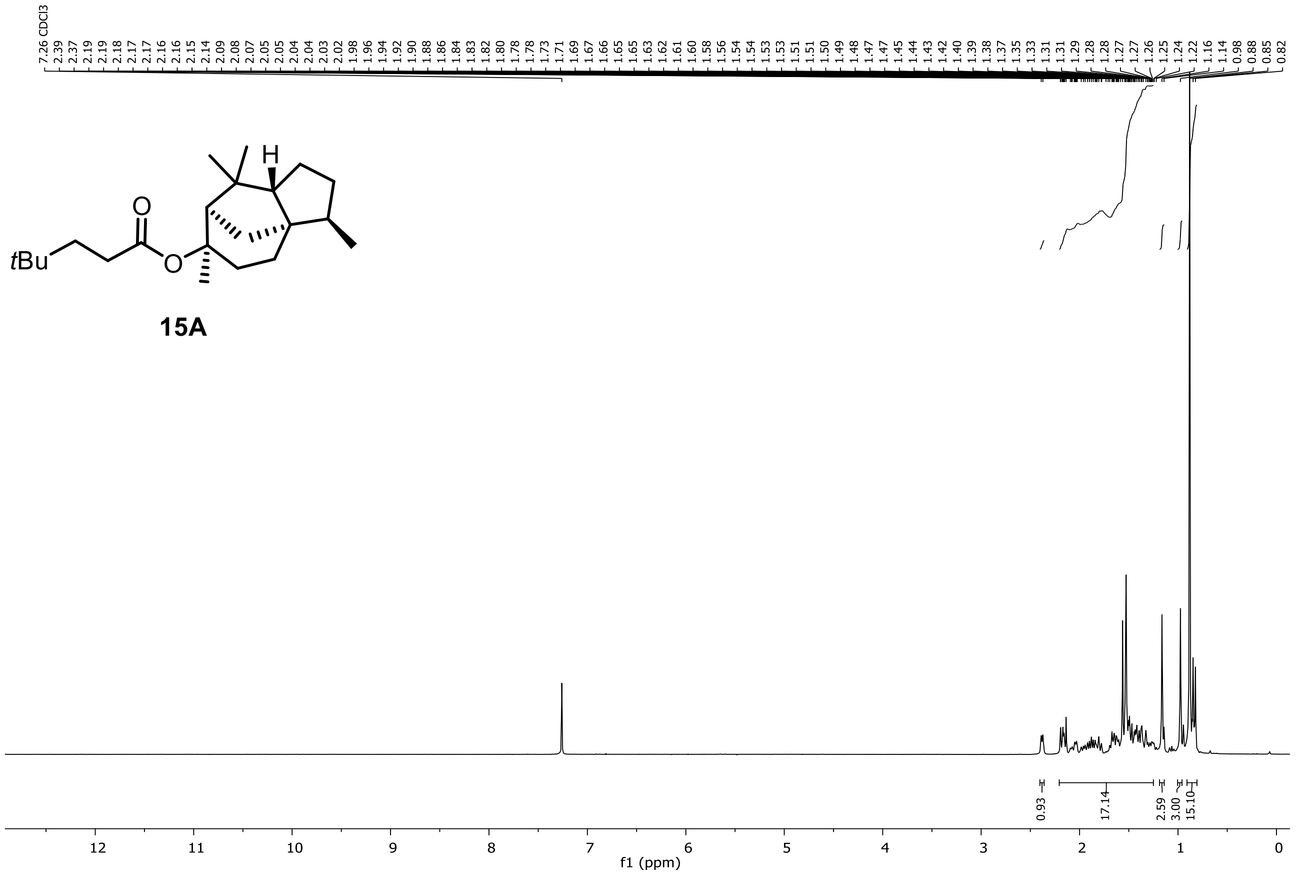

^{13}C NMR (75 MHz, Chloroform-d)

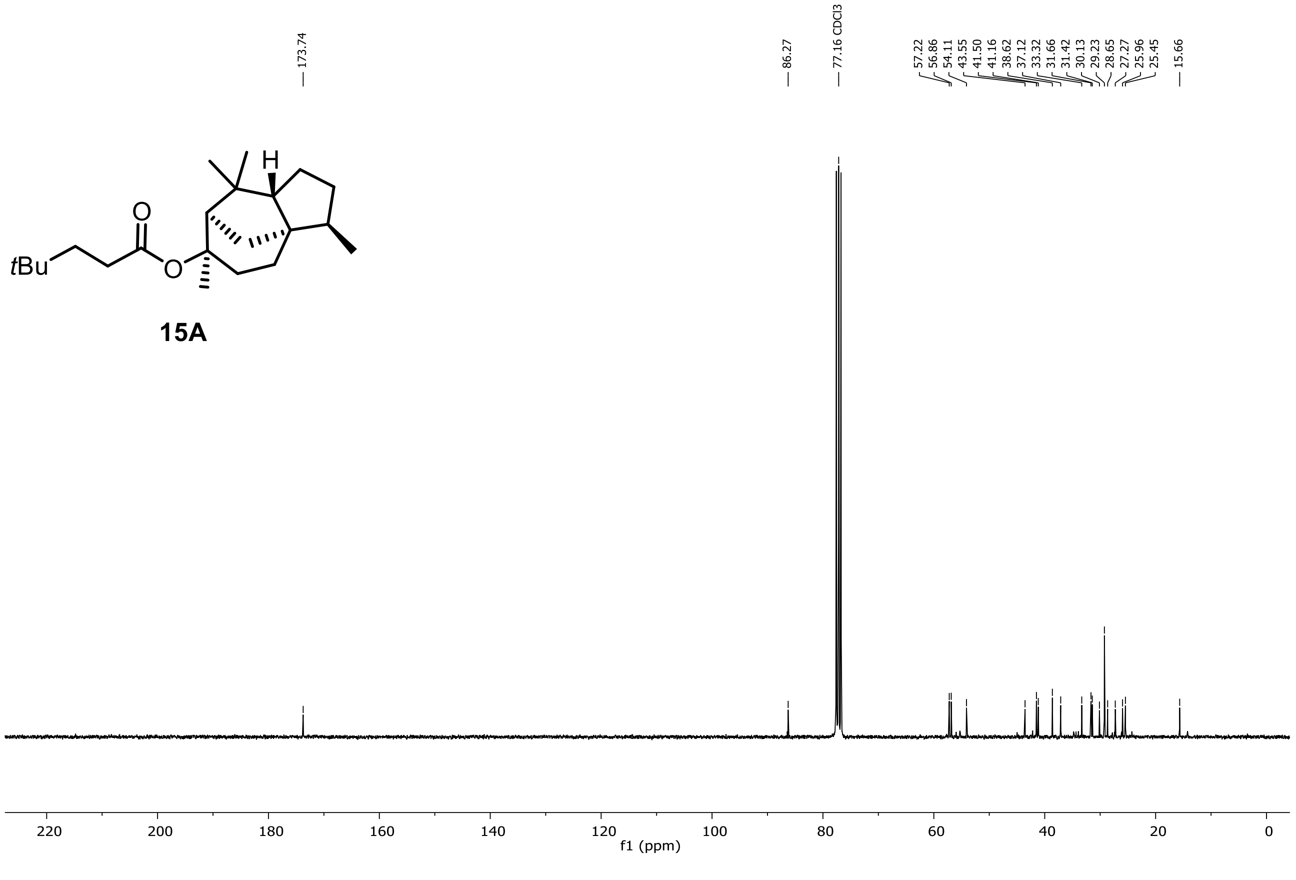

^{1}H NMR (300 MHz, Chloroform-d)

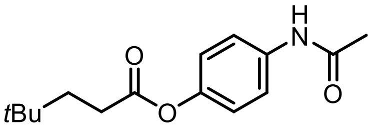

16A

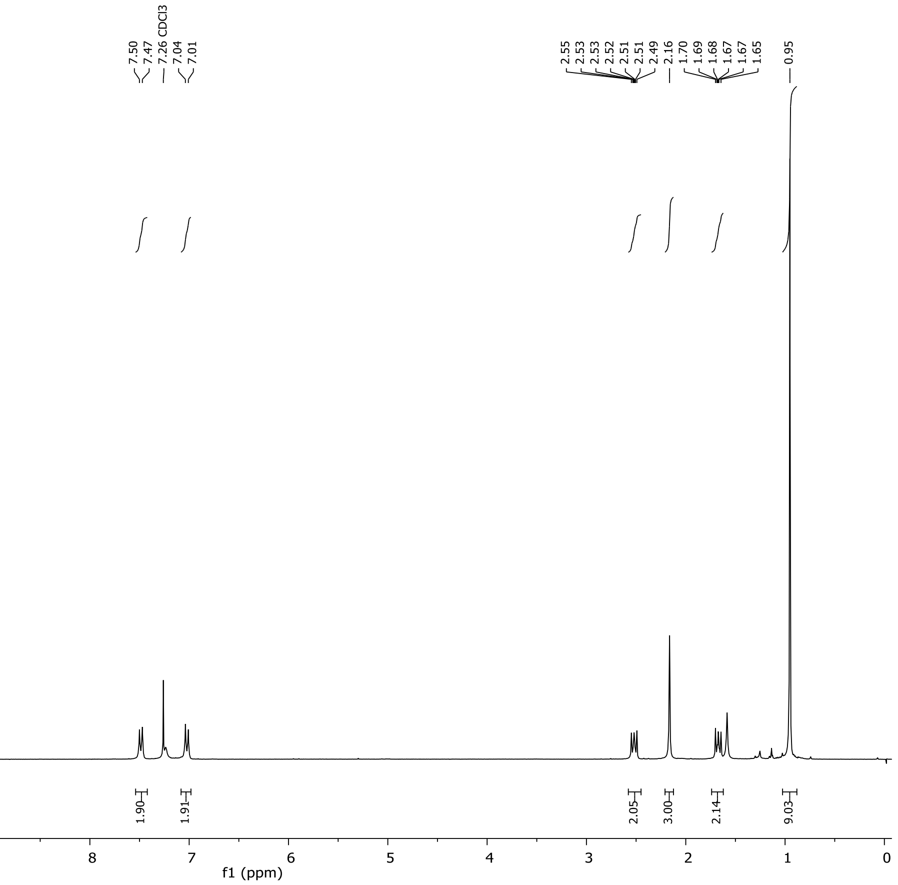

^{13}C NMR (75 MHz, Chloroform-d)

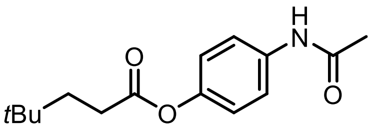

16A

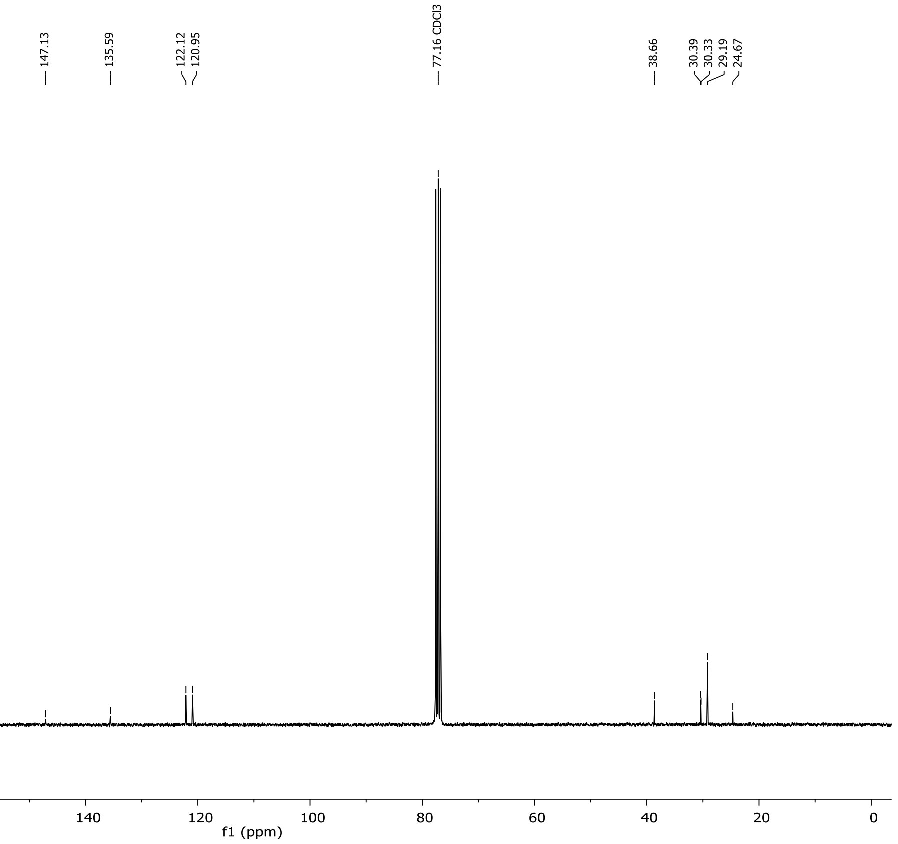

^{1}H NMR (300 MHz, Chloroform-d)

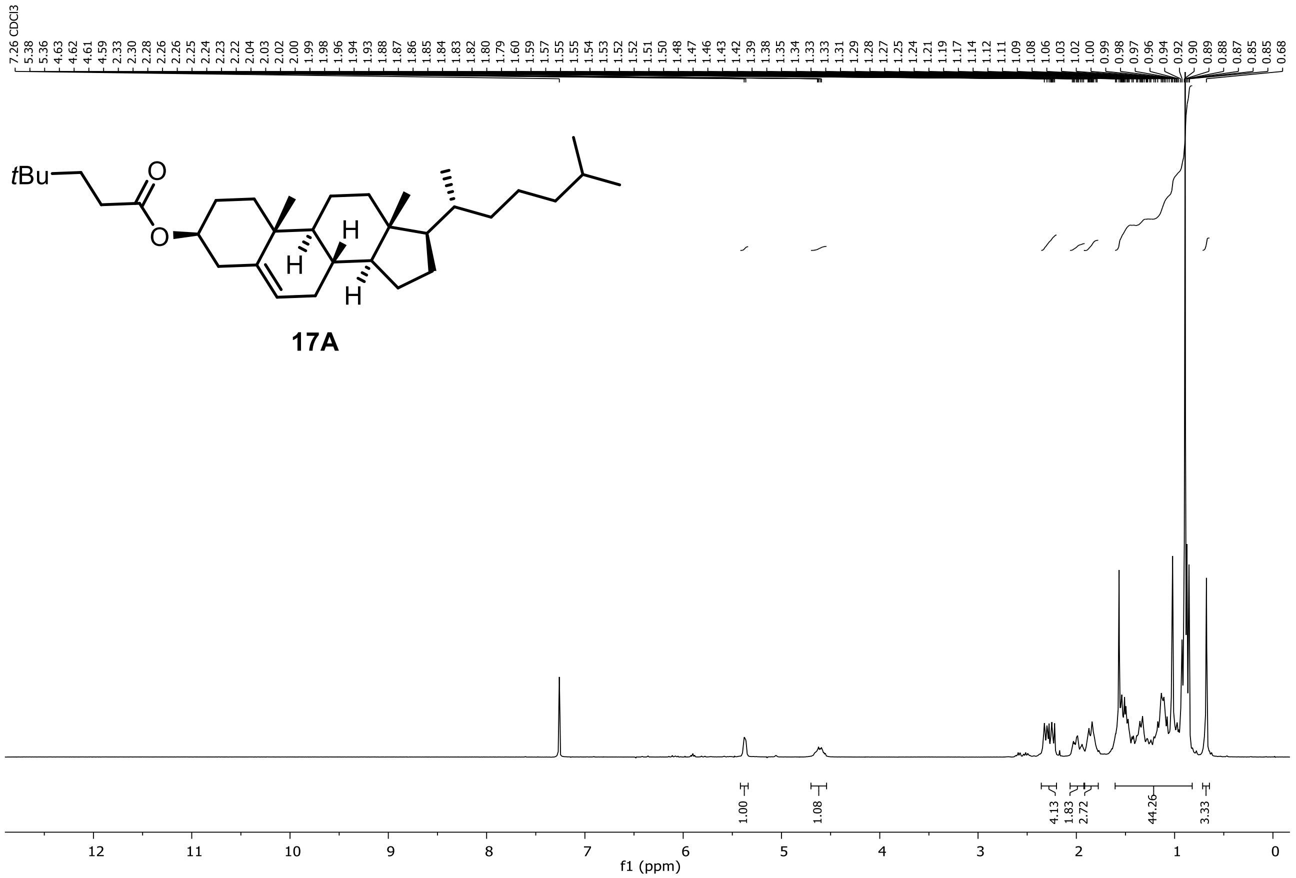

^{13}C NMR (75 MHz, Chloroform-d)

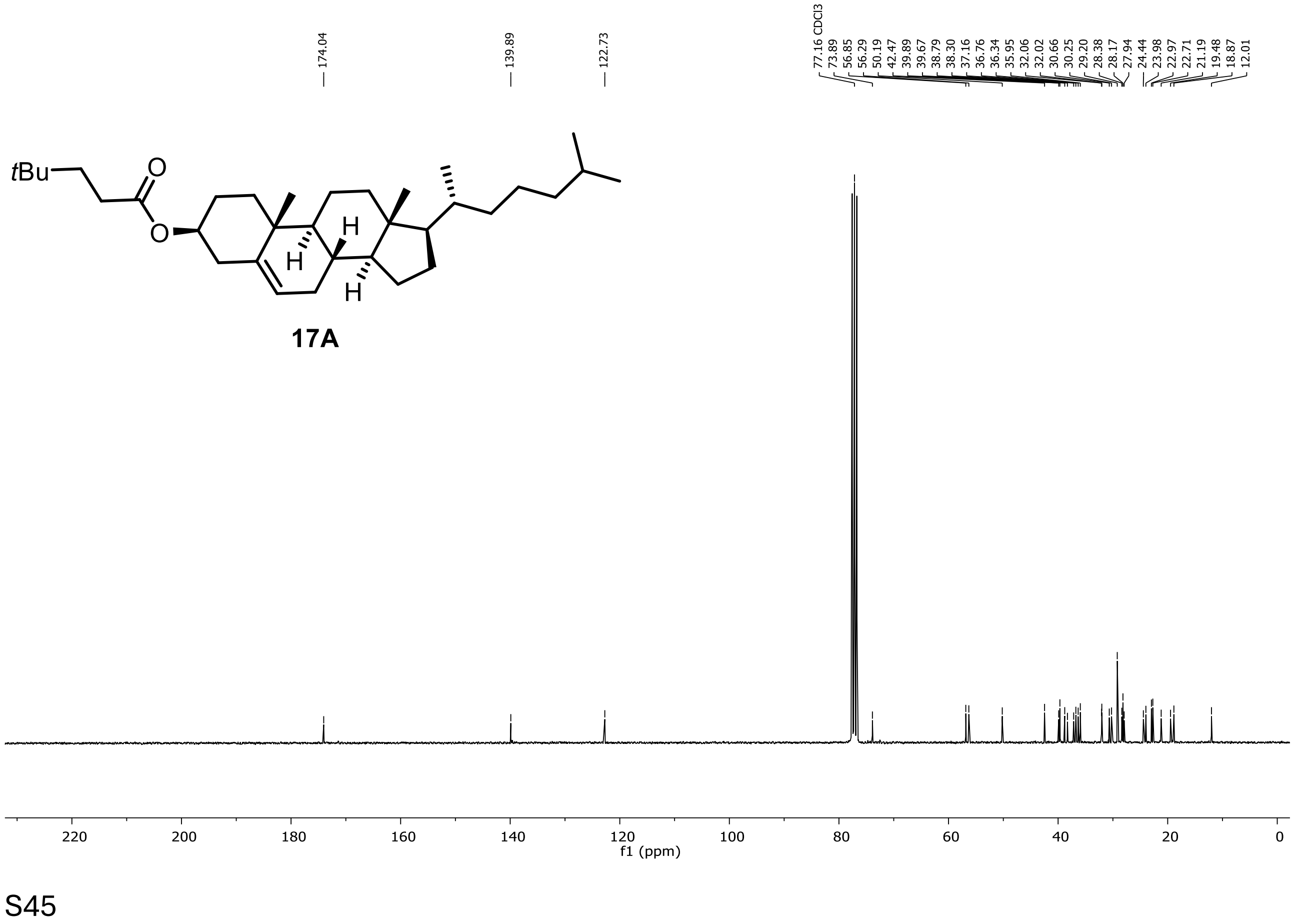

^{1}H NMR (300 MHz, Chloroform-d)

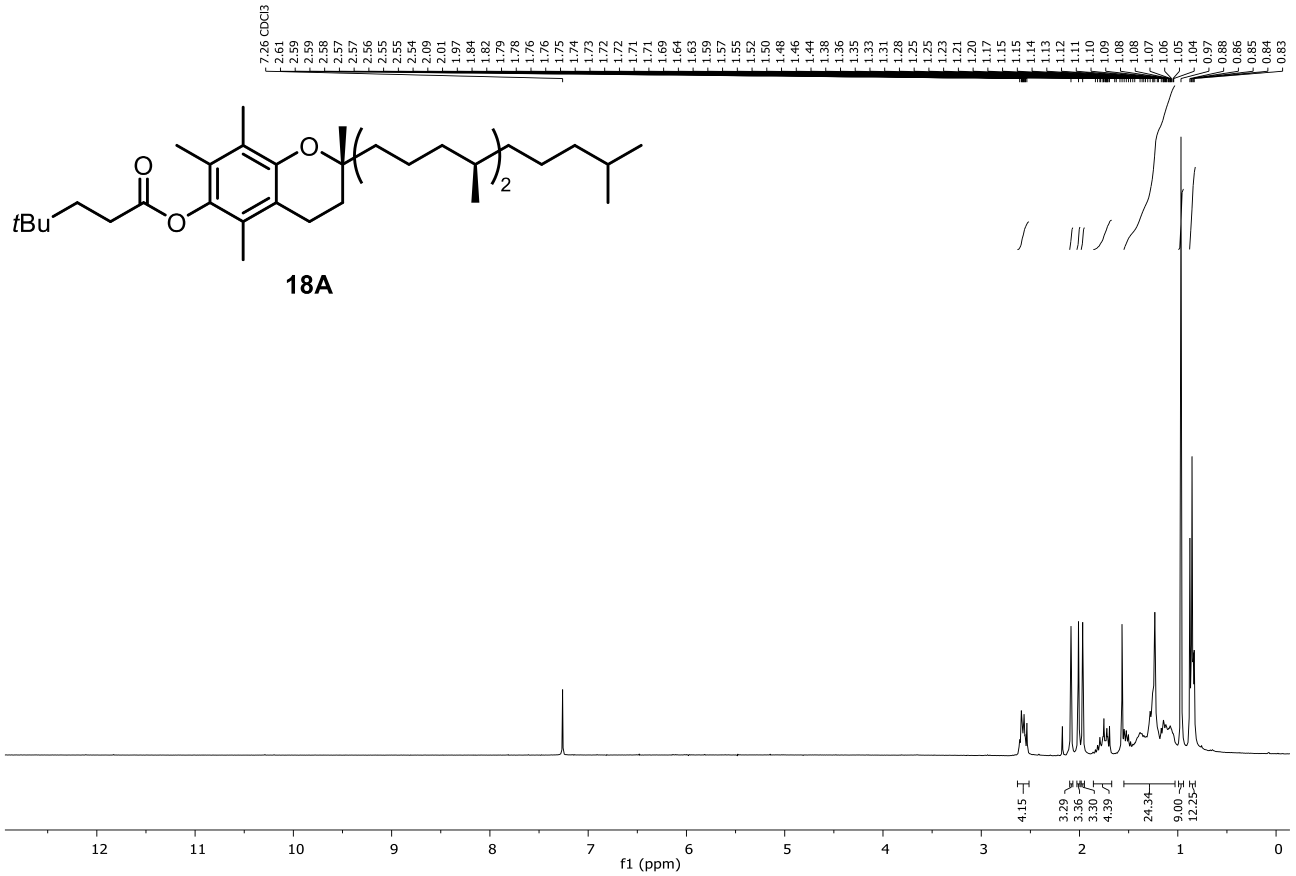

^{13}C NMR (75 MHz, Chloroform-d)

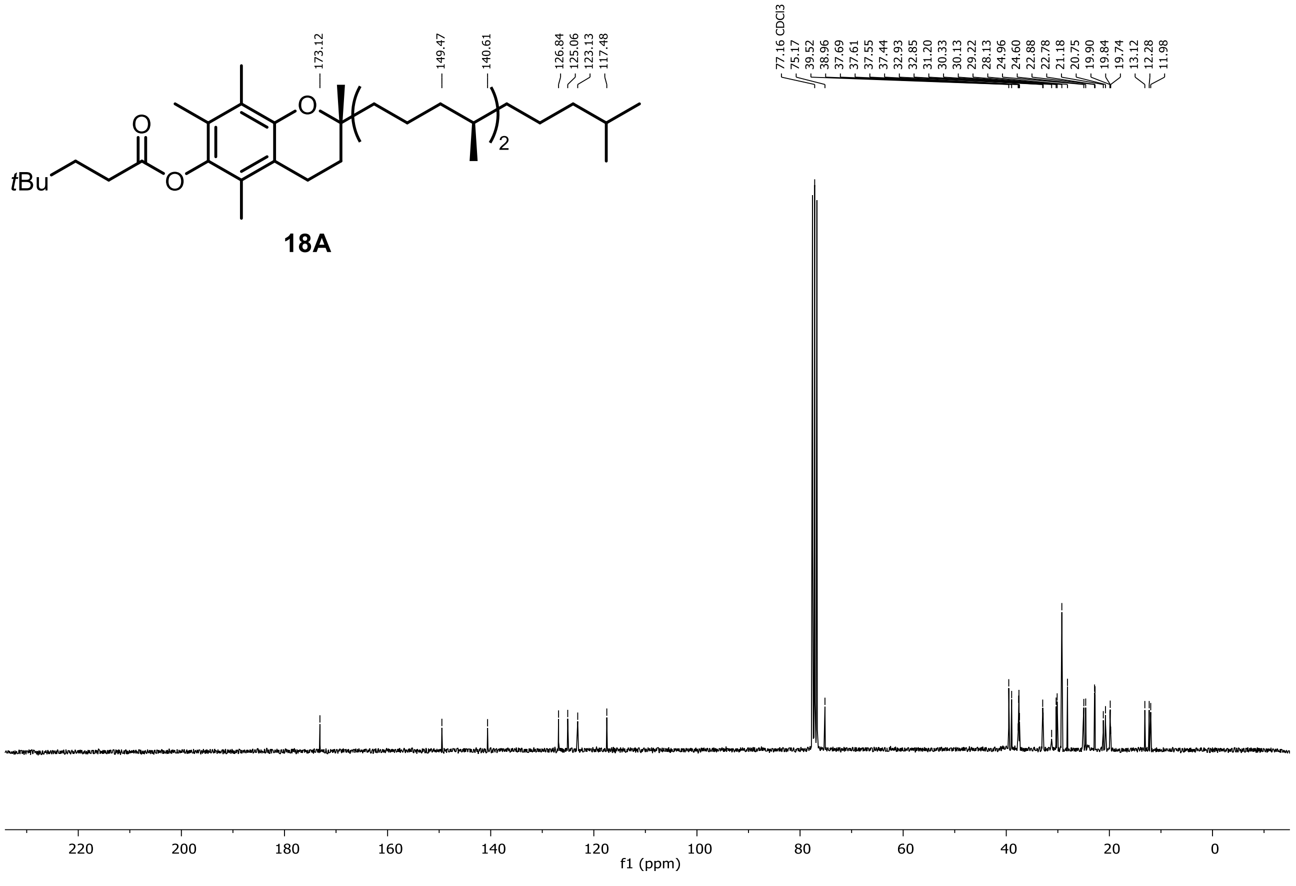

^{1}H NMR (300 MHz, Chloroform-d)

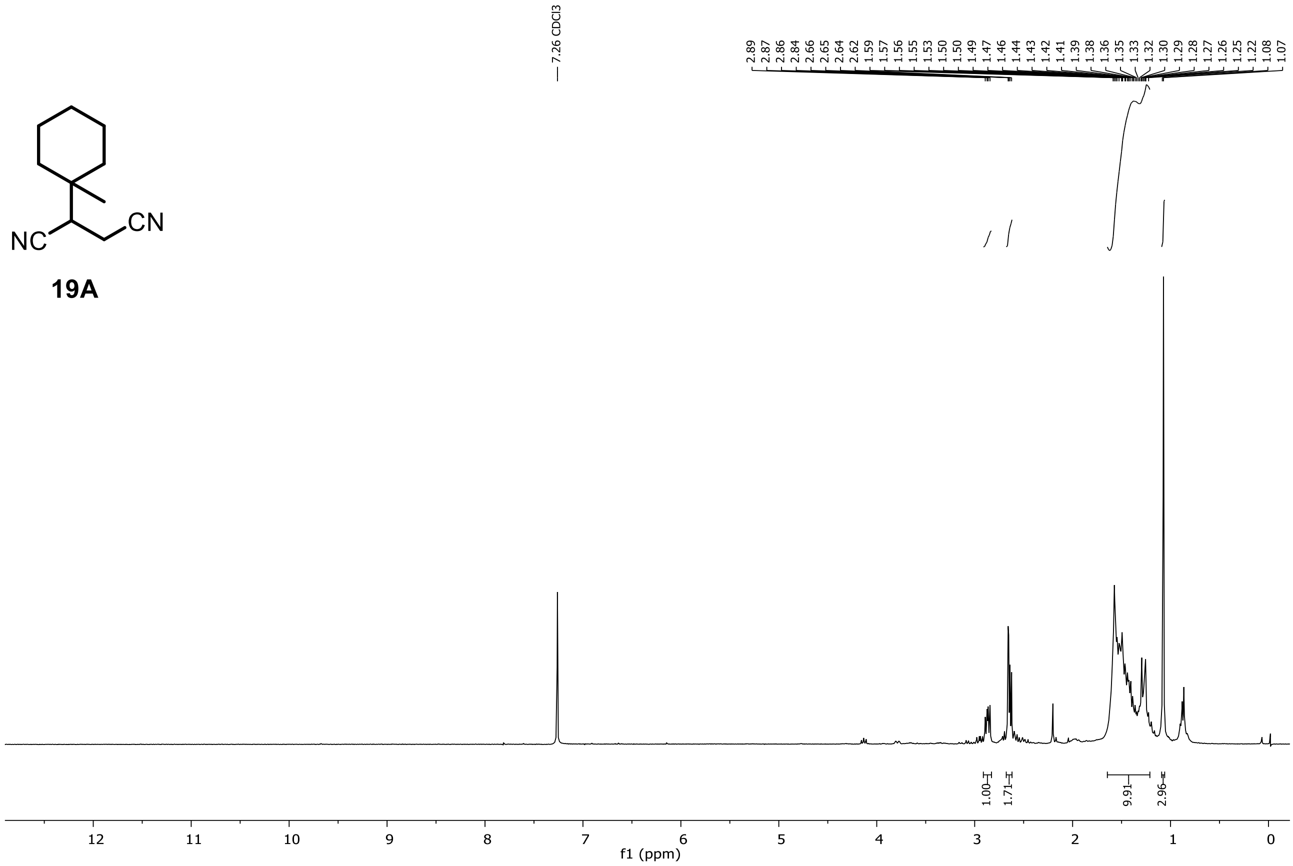

^{13}C NMR (75 MHz, Chloroform-d)

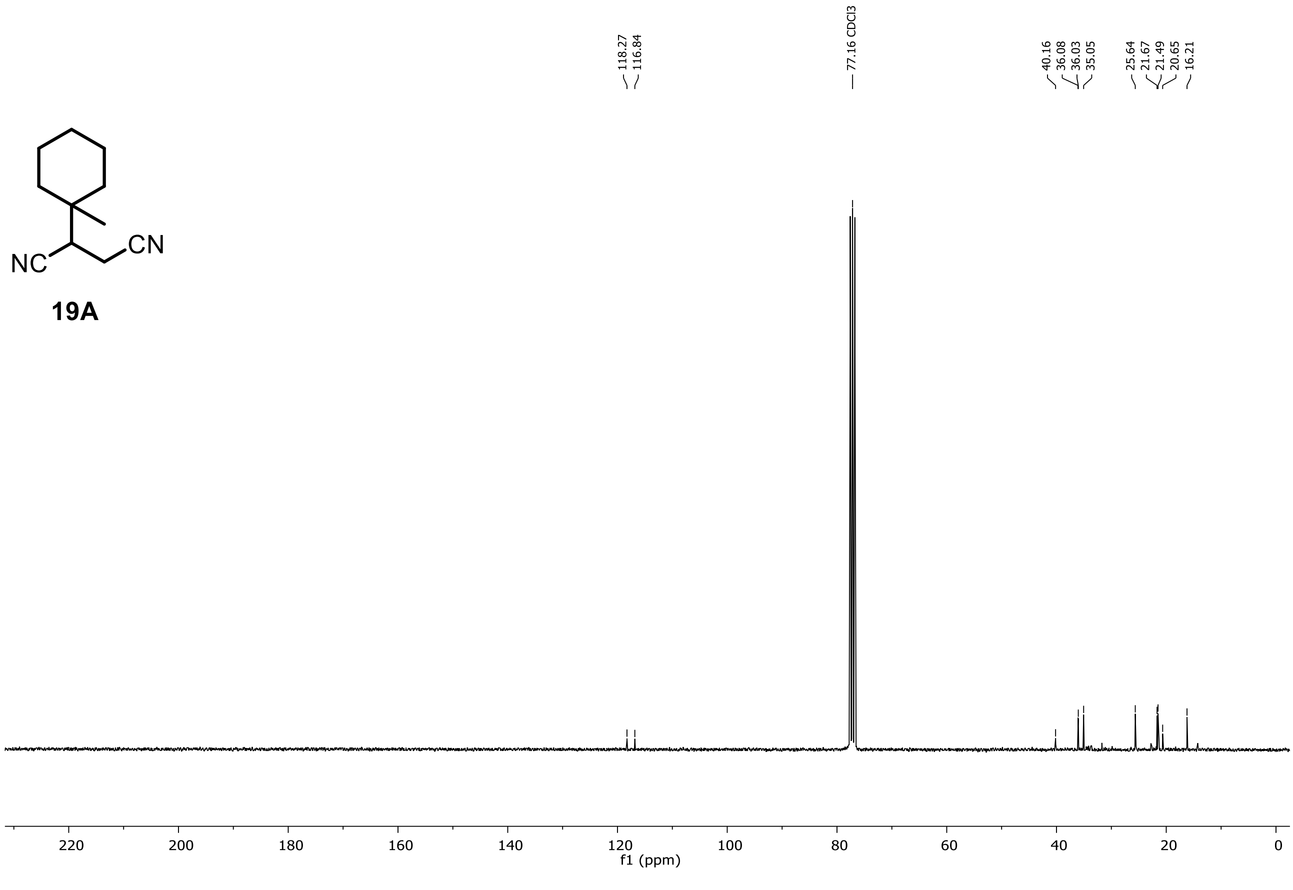

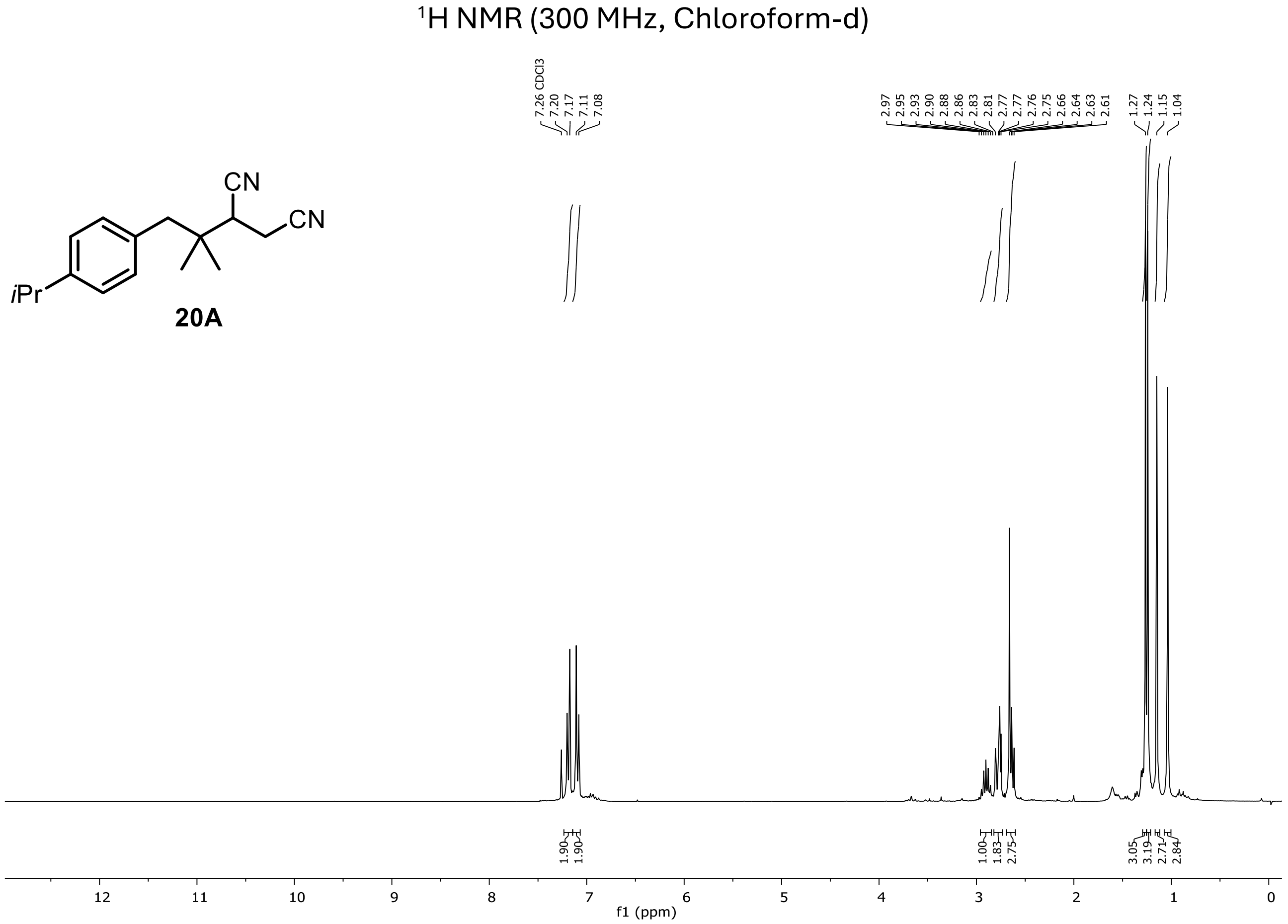

¹H NMR (300 MHz, Chloroform-d)
CN
CN
iPr
20A
7.26 CDCl3
7.20
7.17
7.11
7.08
2.97
2.95
2.93
2.90
2.88
2.86
2.83
2.81
2.77
2.77
2.76
2.75
2.66
2.64
2.63
2.61
1.27
1.24
1.15
1.04
1.90
1.90
1.00
1.83
2.75
3.05
3.19
2.71
2.84
12
11
10
9
8
7
6
5
4
3
2
1
0
f1 (ppm)

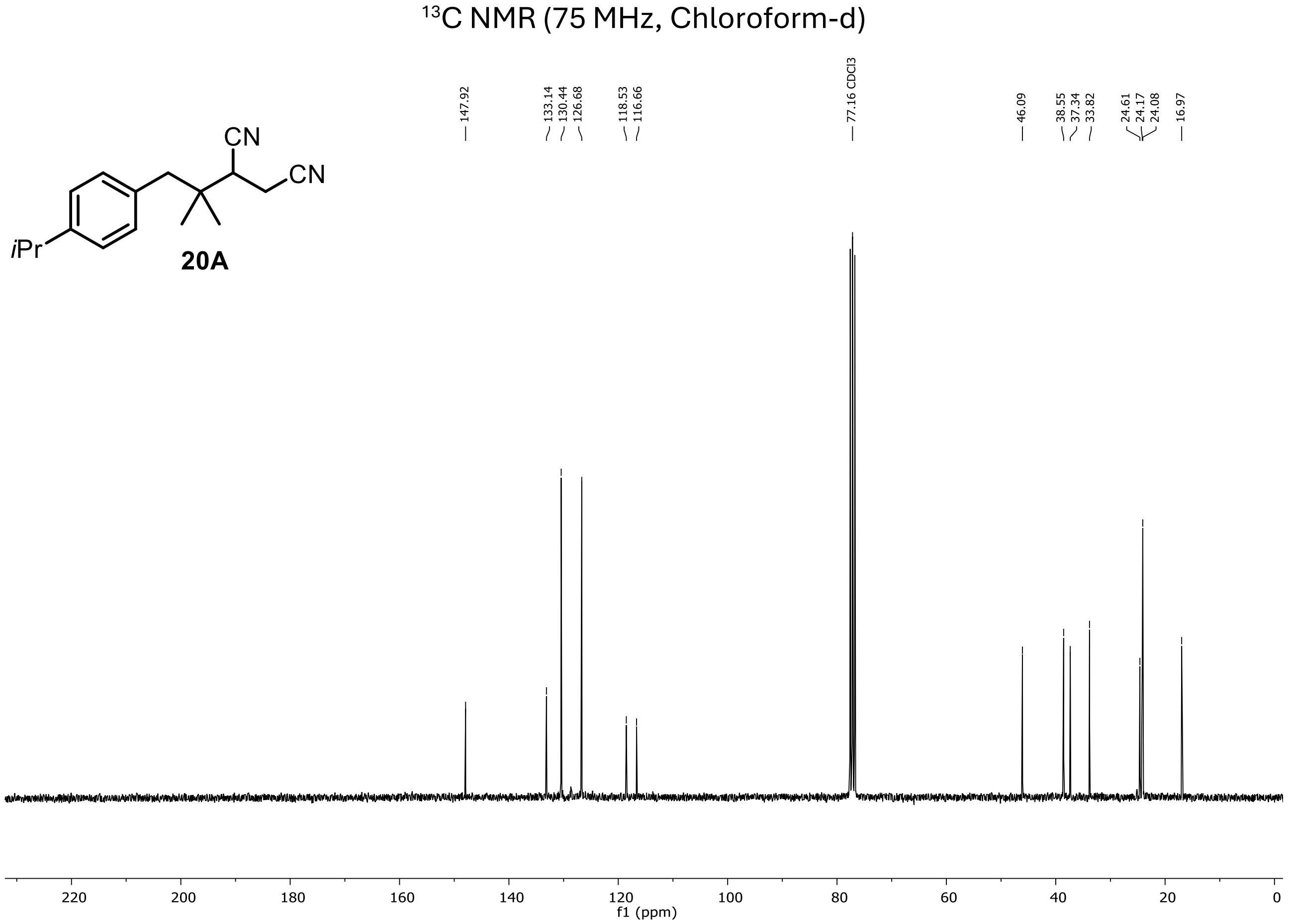

¹³C NMR (75 MHz, Chloroform-d)
CN
CN
iPr
20A
147.92
133.14
130.44
126.68
118.53
116.66
77.16 CDCl3
46.09
38.55
37.34
33.82
24.61
24.17
24.08
16.97
220
200
180
160
140
120
100
80
60
40
20
0
f1 (ppm)

^{1}H NMR (300 MHz, Chloroform-d)

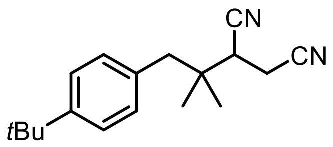

21A

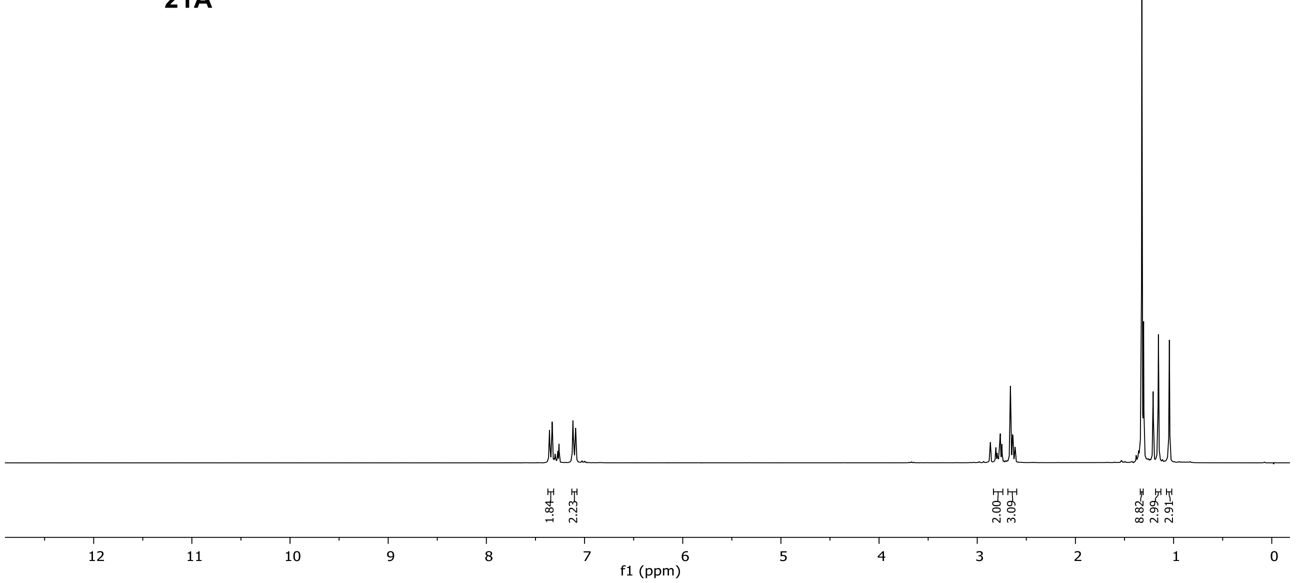

^{13}C NMR (75 MHz, Chloroform-d)

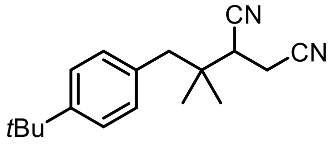

21A

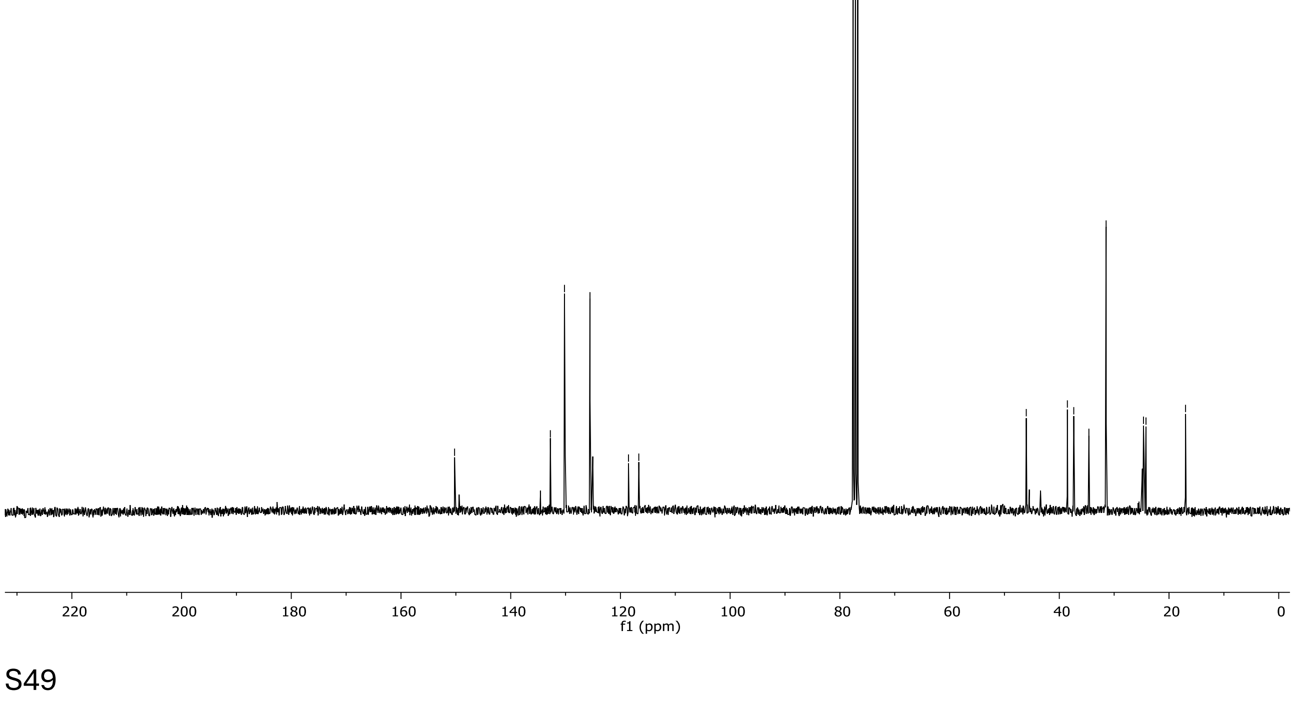

^{1}H NMR (300 MHz, Chloroform-d)

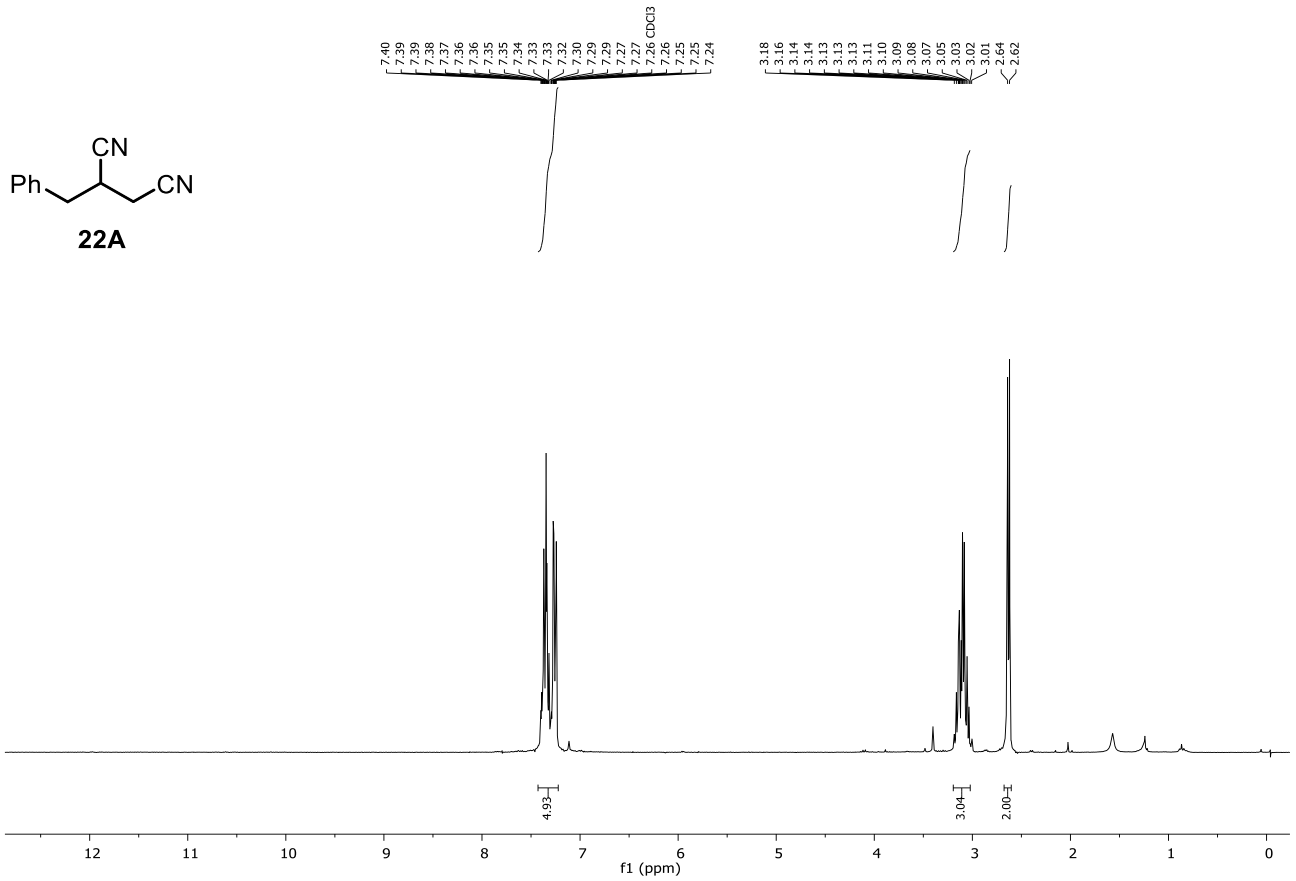

^{13}C NMR (75 MHz, Chloroform-d)

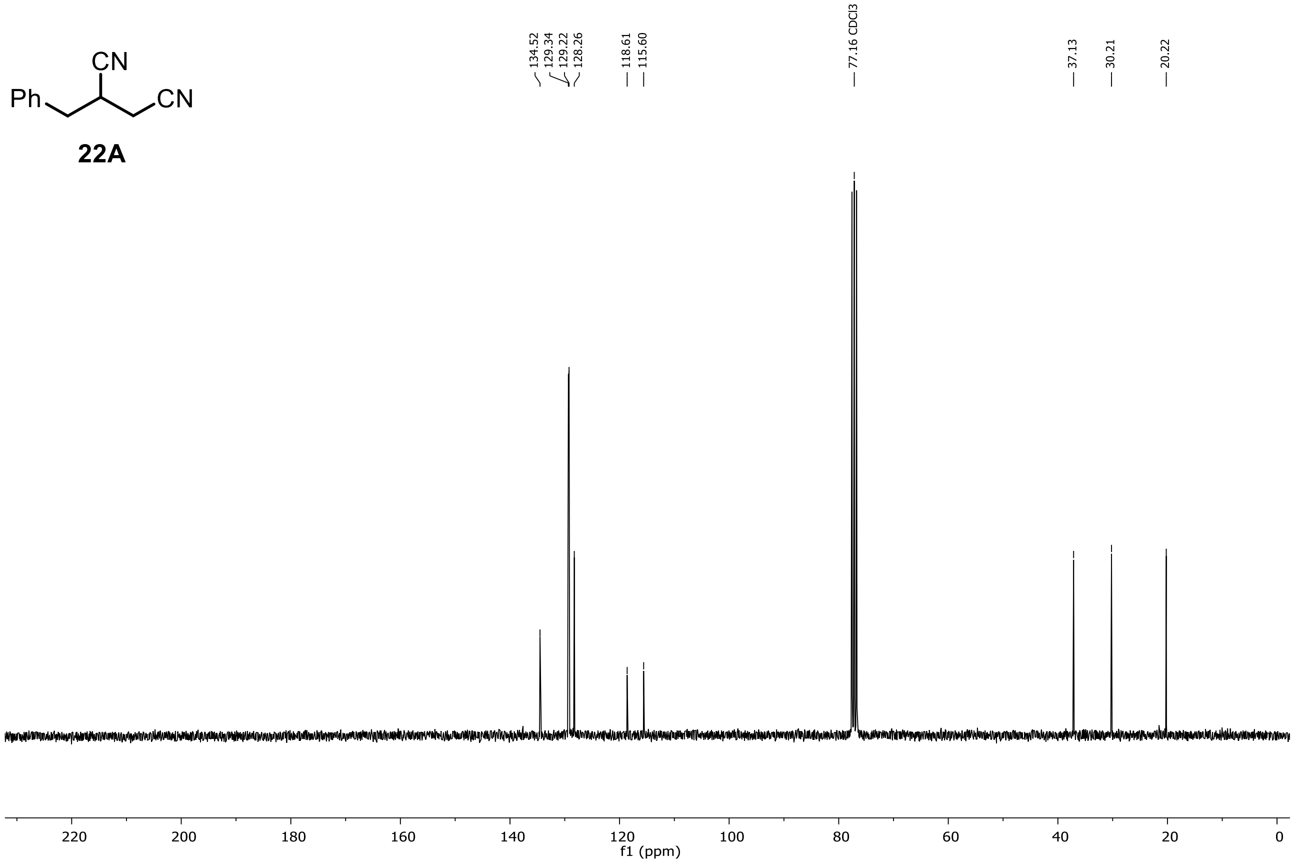

^{1}H NMR (300 MHz, Chloroform-d)

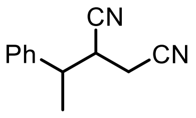

23A

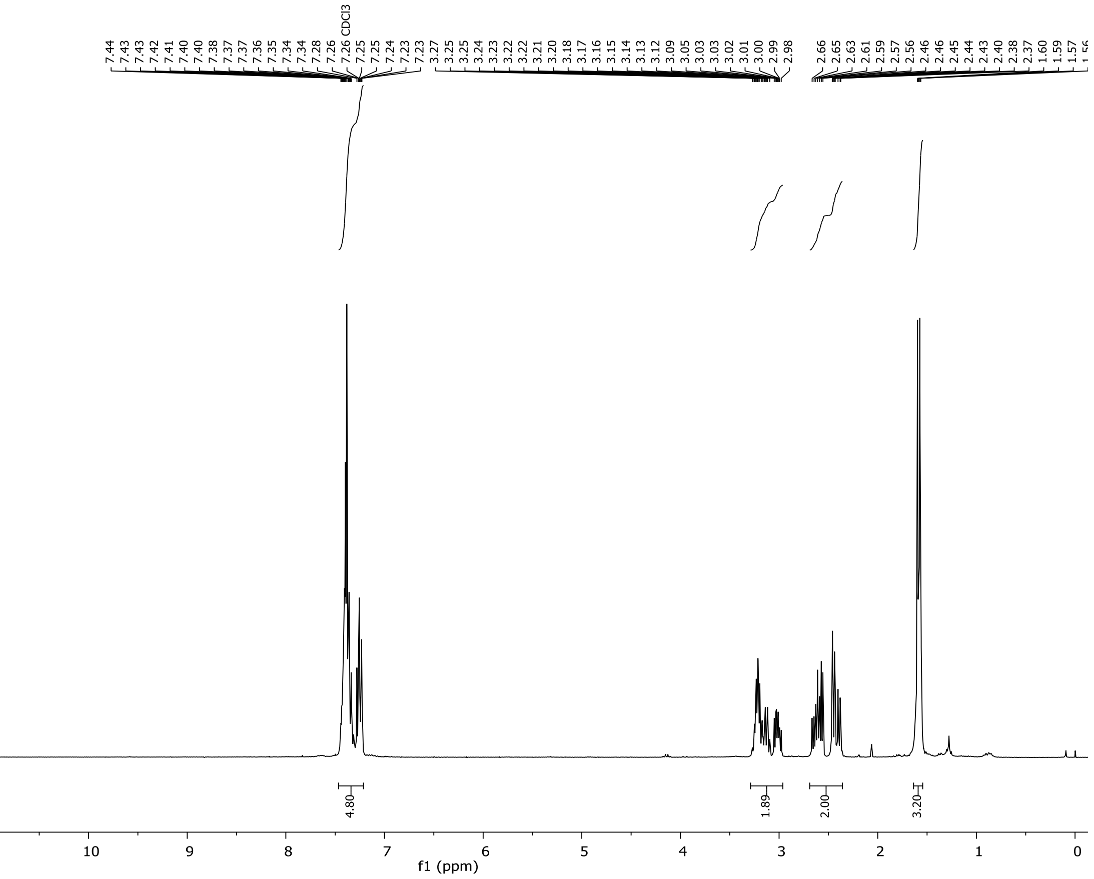

^{13}C NMR (75 MHz, Chloroform-d)

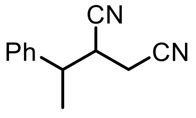

23A

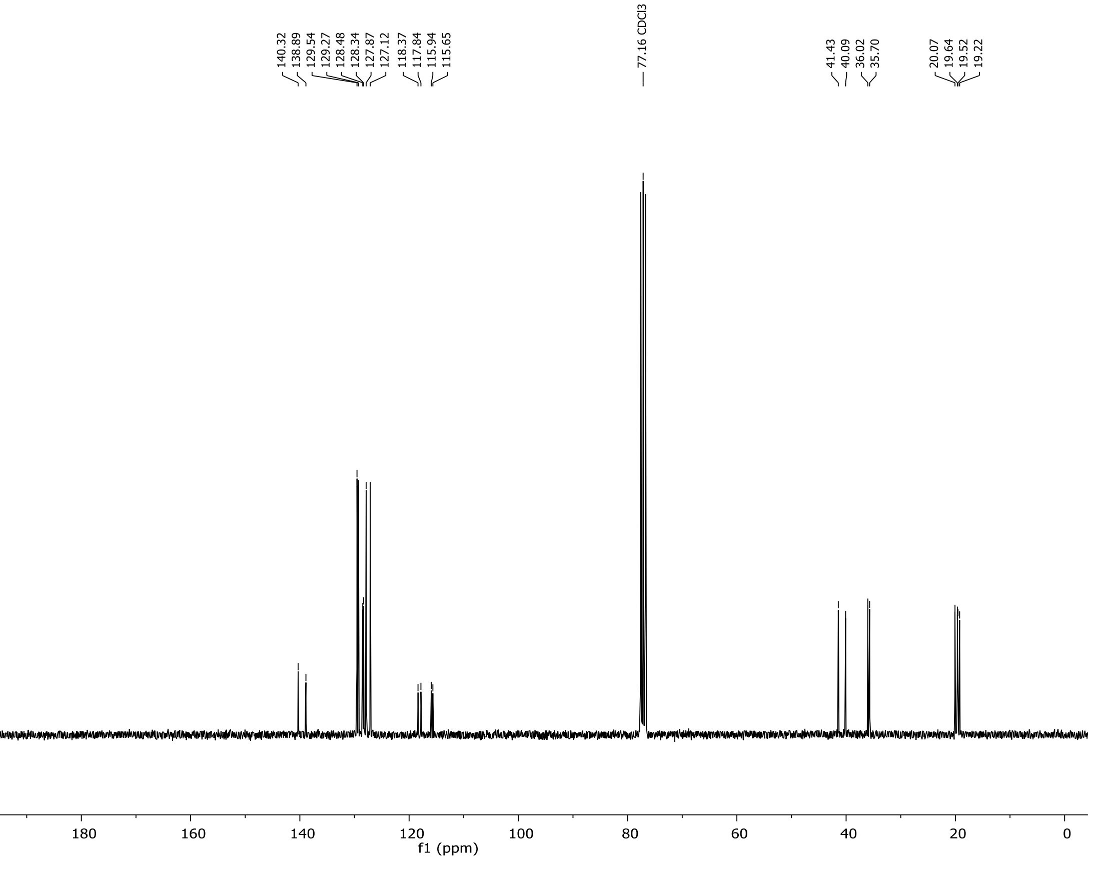

^{1}H NMR (300 MHz, Chloroform-d)

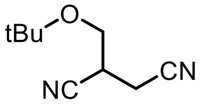

24A

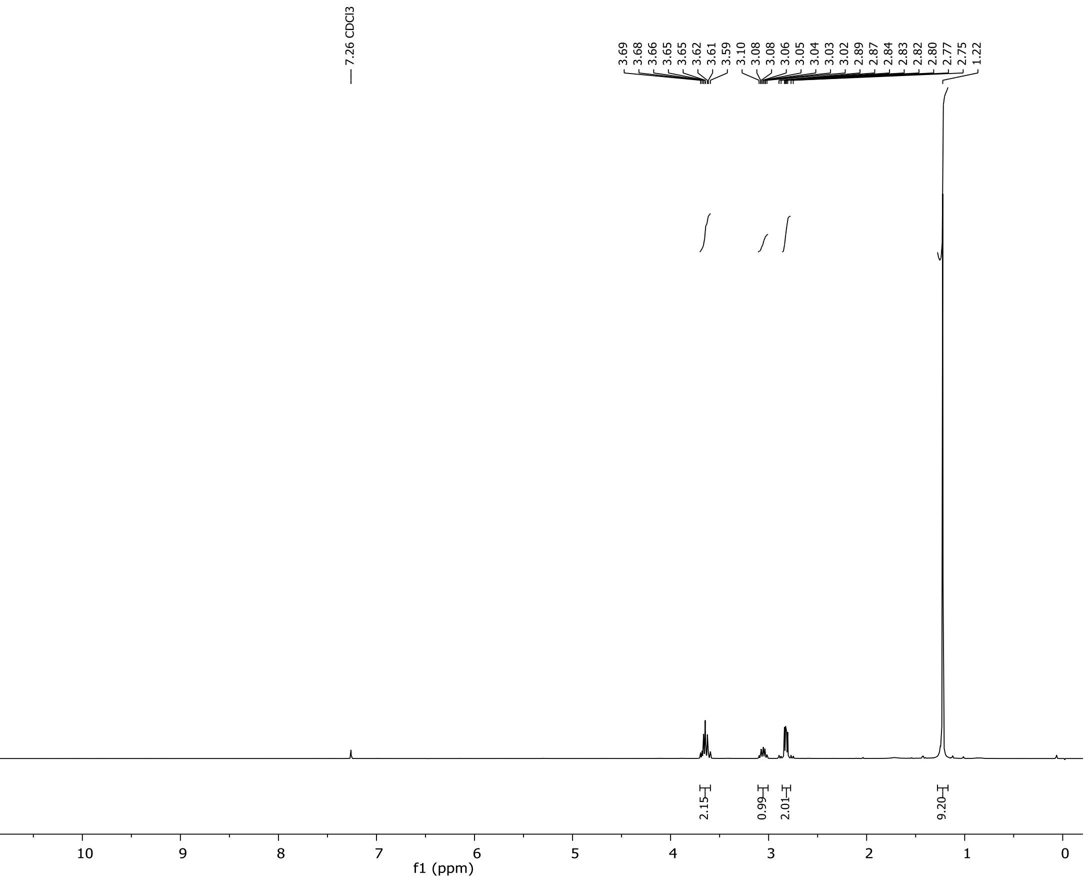

^{13}C NMR (75 MHz, Chloroform-d)

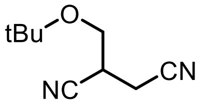

24A

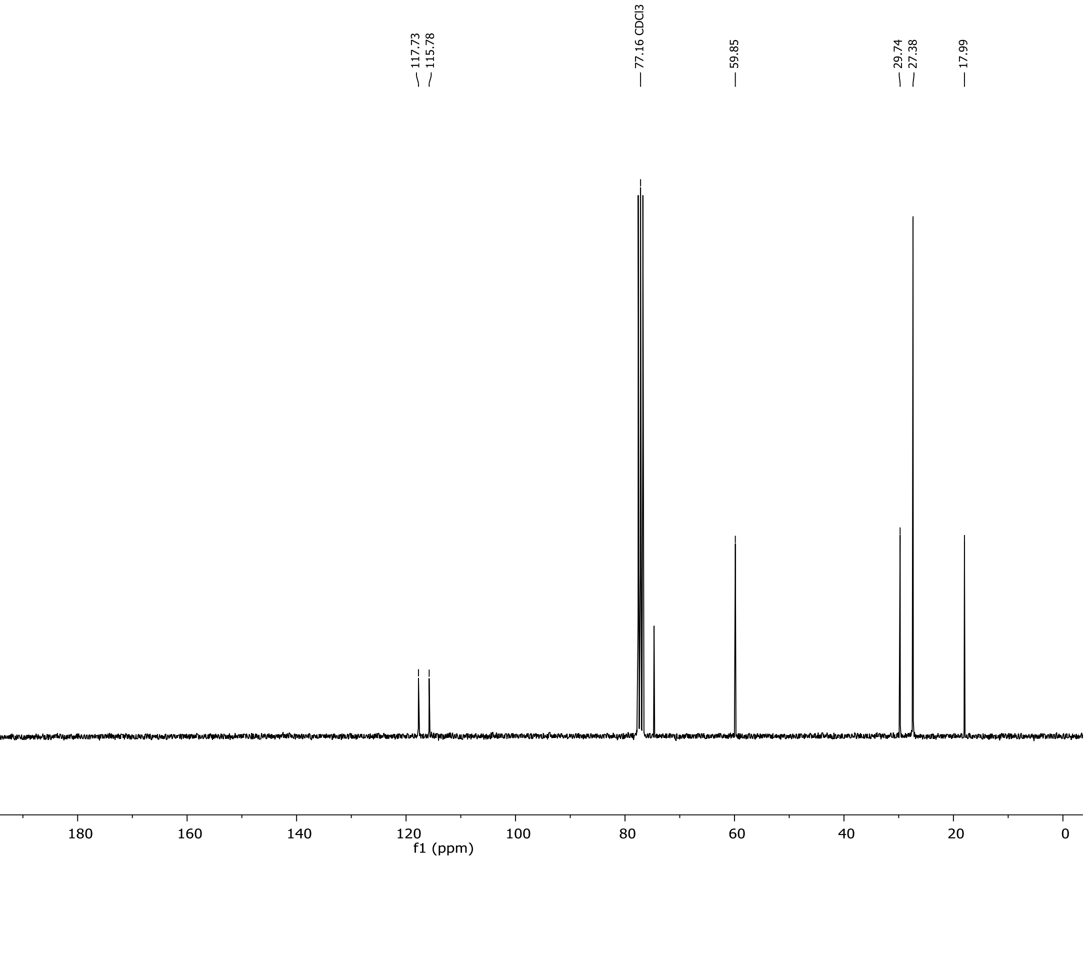

^{1}H NMR (300 MHz, Chloroform-d)

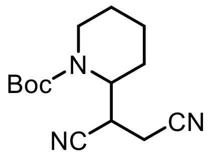

25A

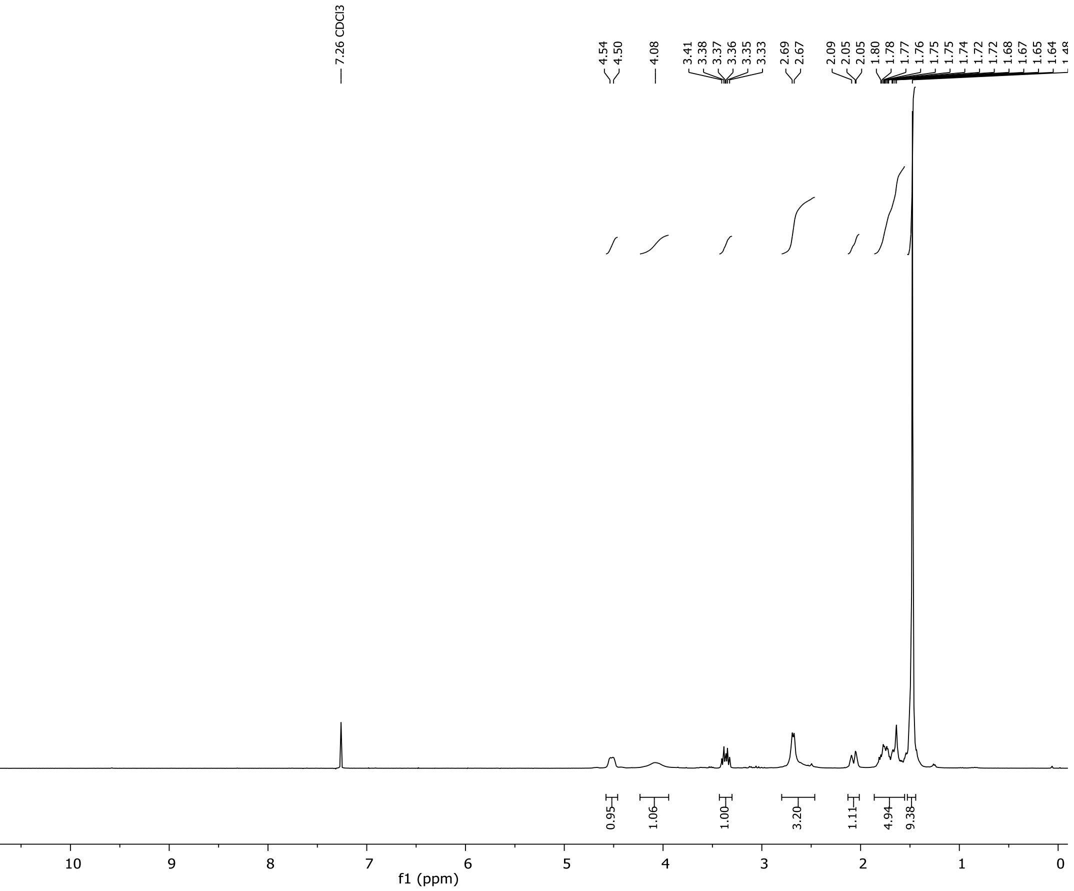

^{13}C NMR (75 MHz, Chloroform-d)

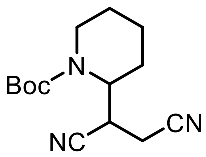

25A

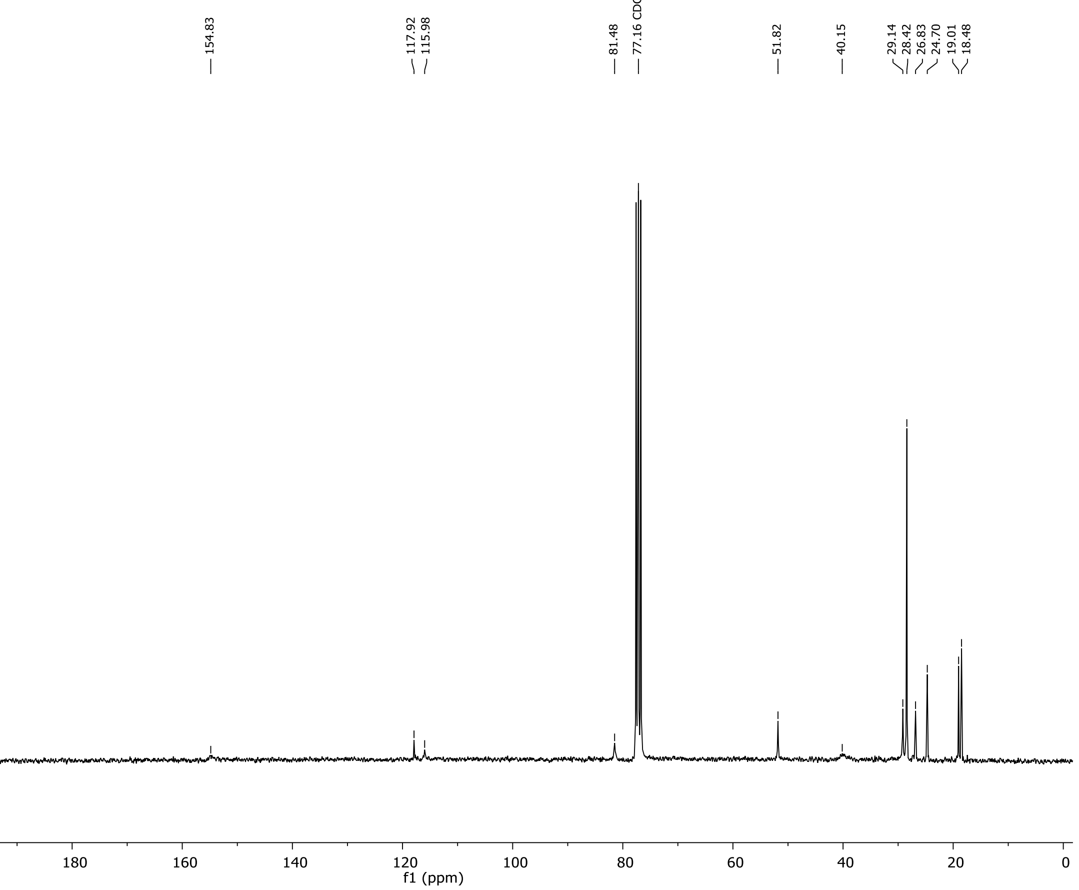

^{1}H NMR (300 MHz, Chloroform-d)

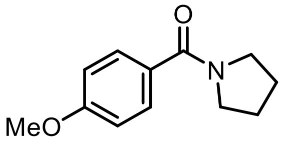

5B

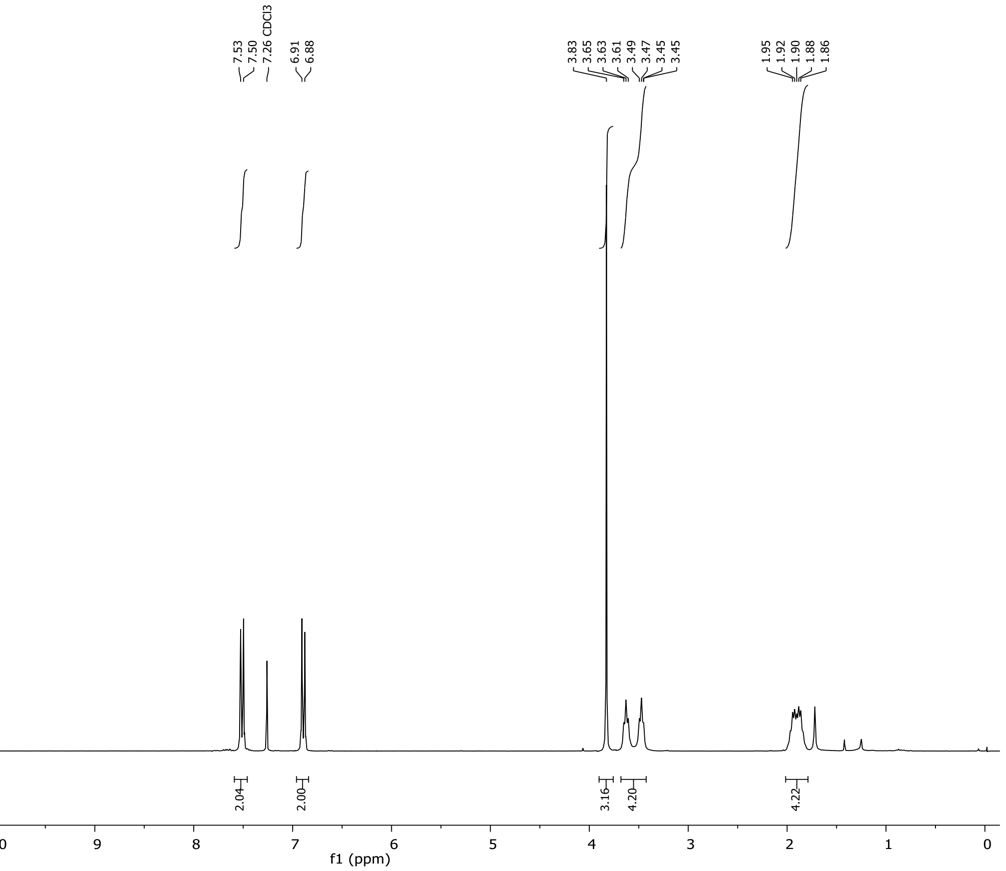

^{13}C NMR (75 MHz, Chloroform-d)

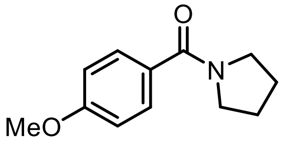

5B

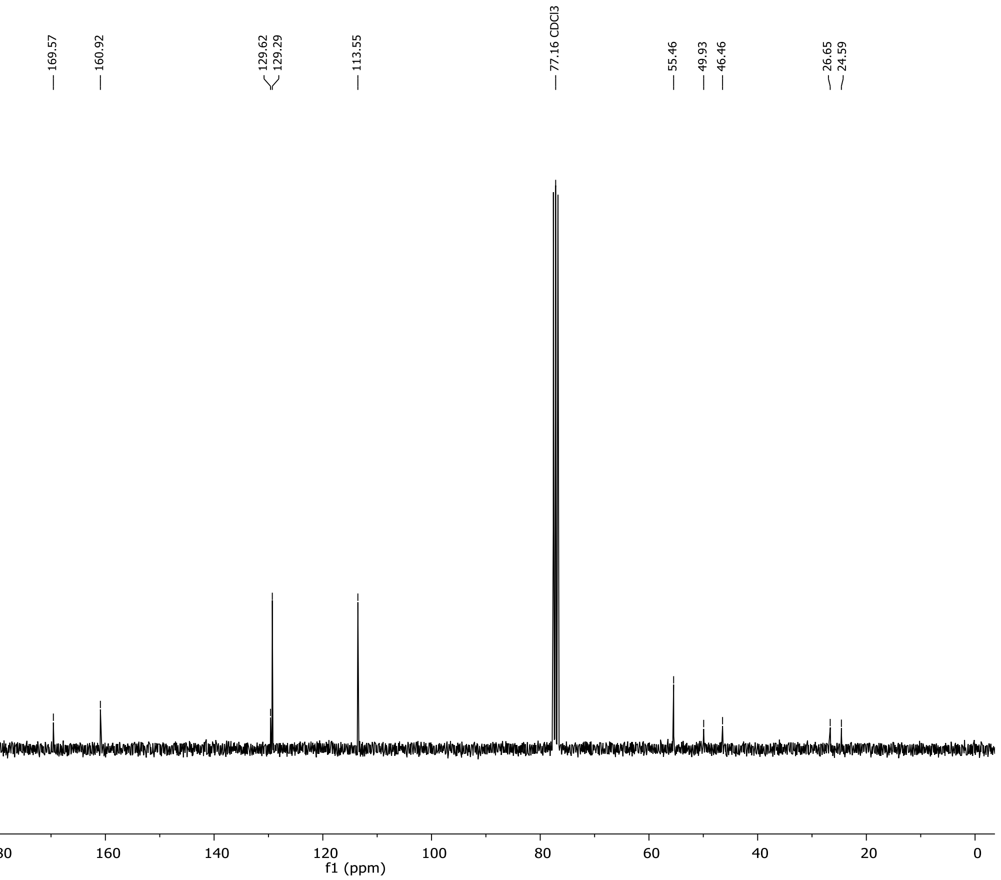

^{1}H NMR (300 MHz, Chloroform-d)

6B

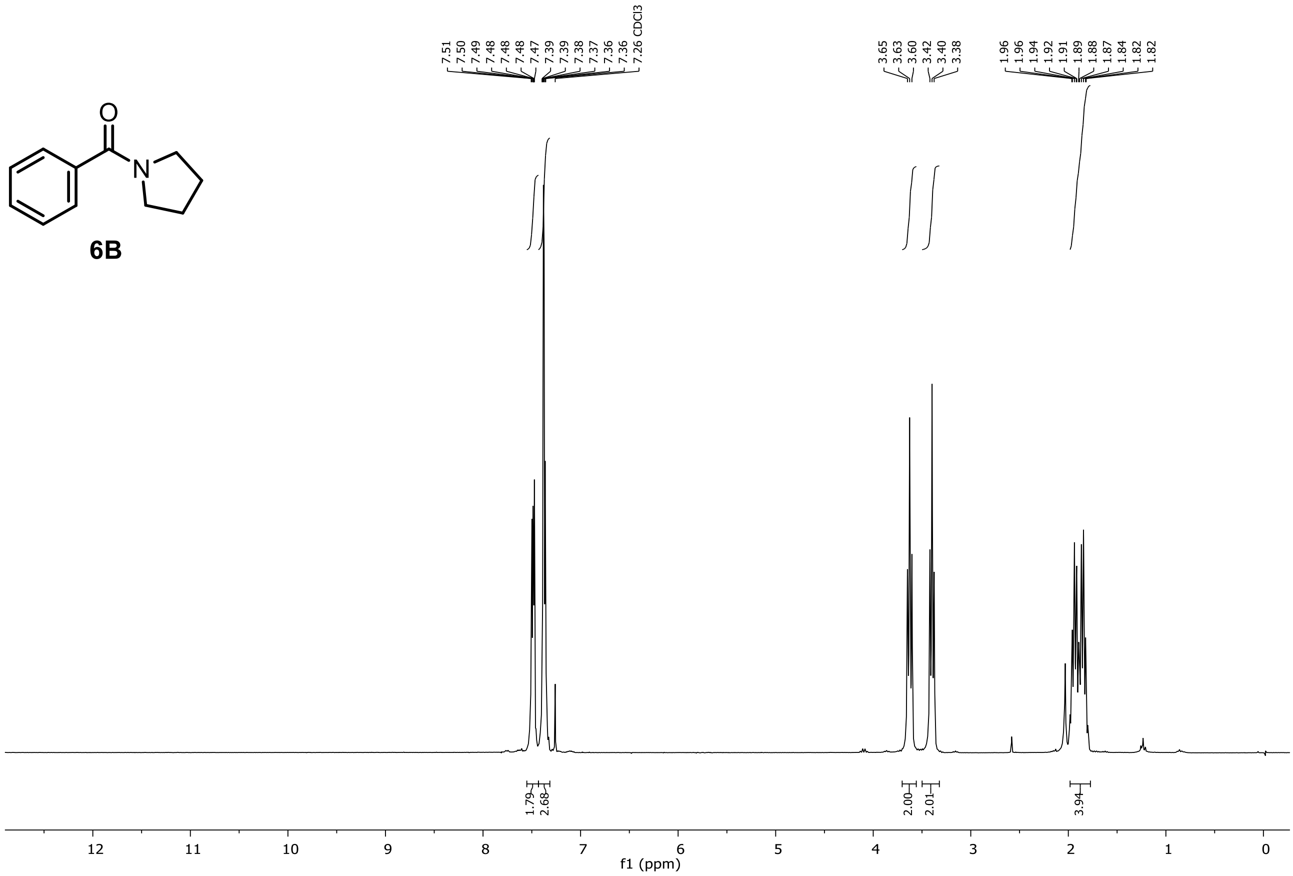

^{13}C NMR (75 MHz, Chloroform-d)

6B

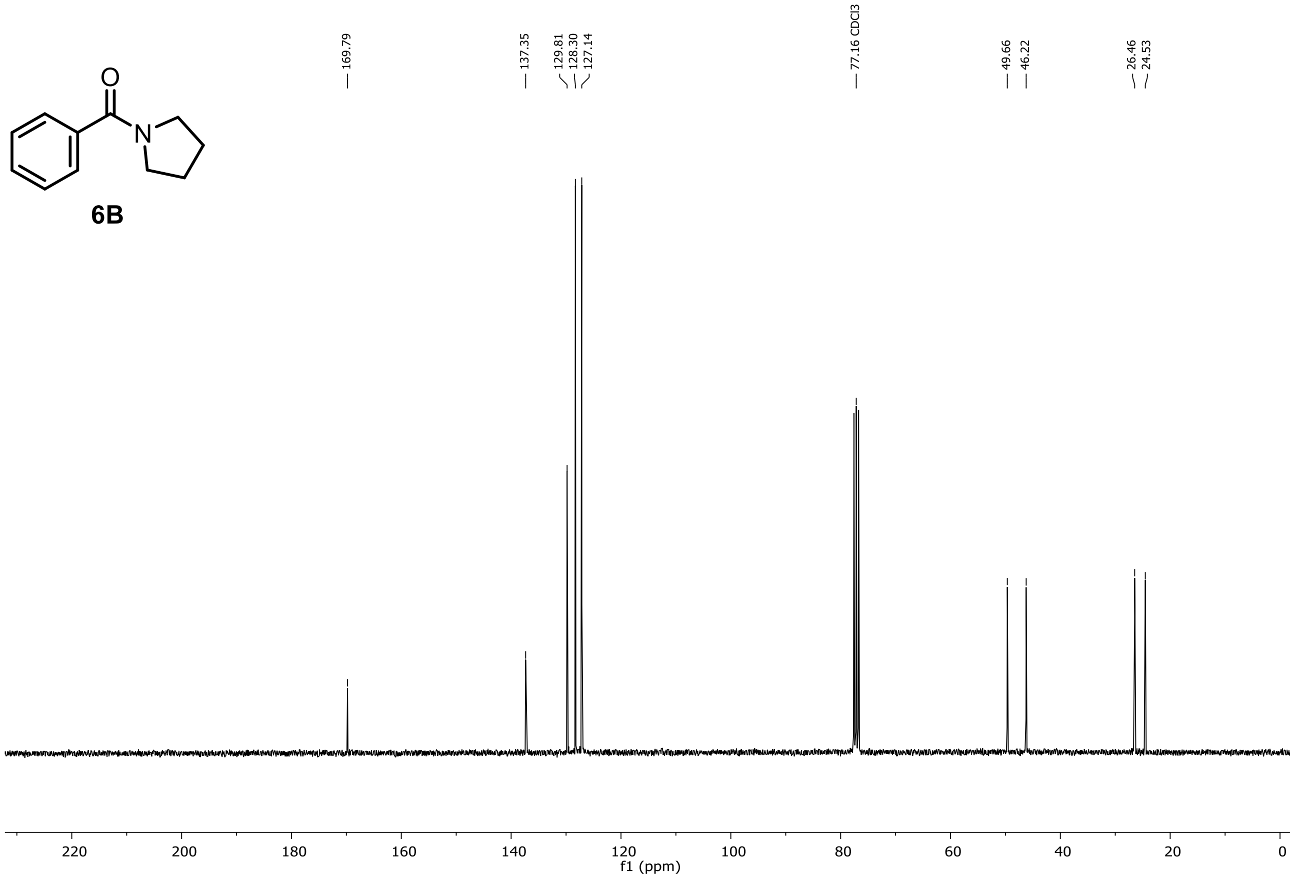

^{1}H NMR (300 MHz, Chloroform-d)

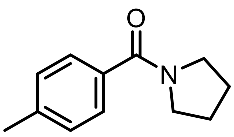

7B

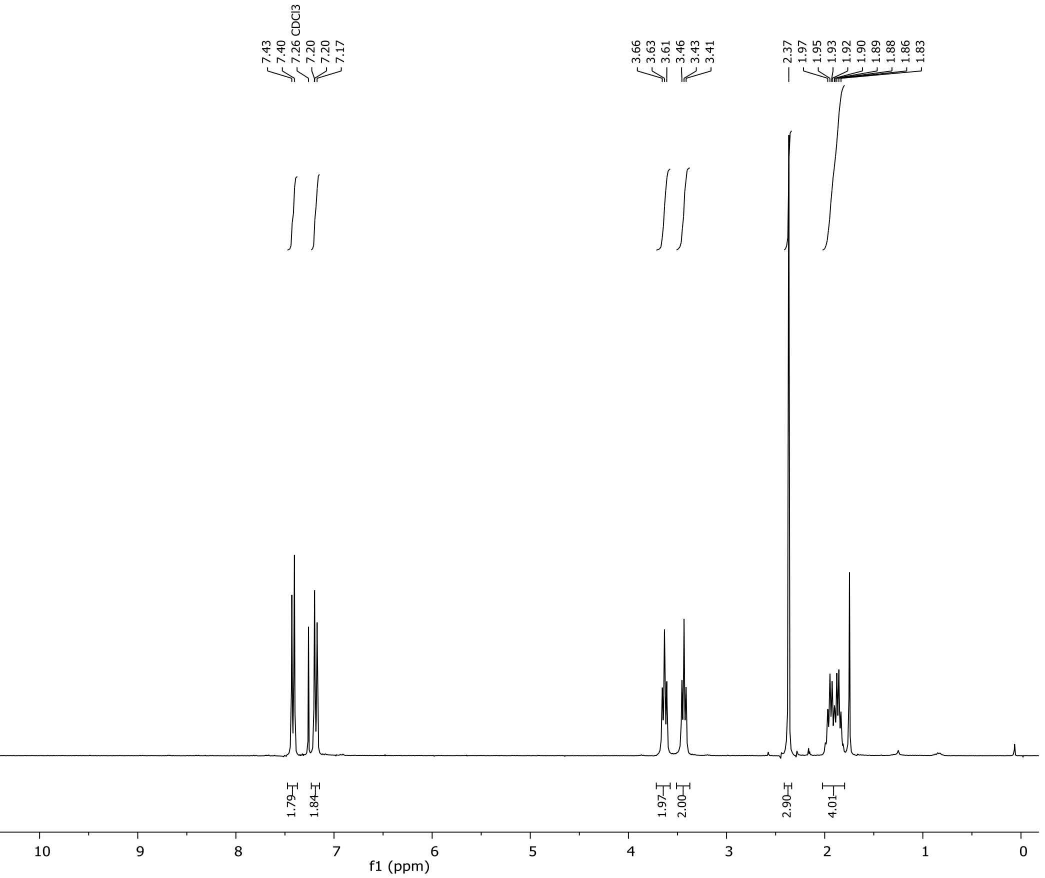

^{13}C NMR (75 MHz, Chloroform-d)

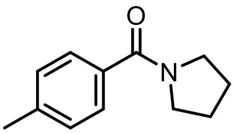

7B

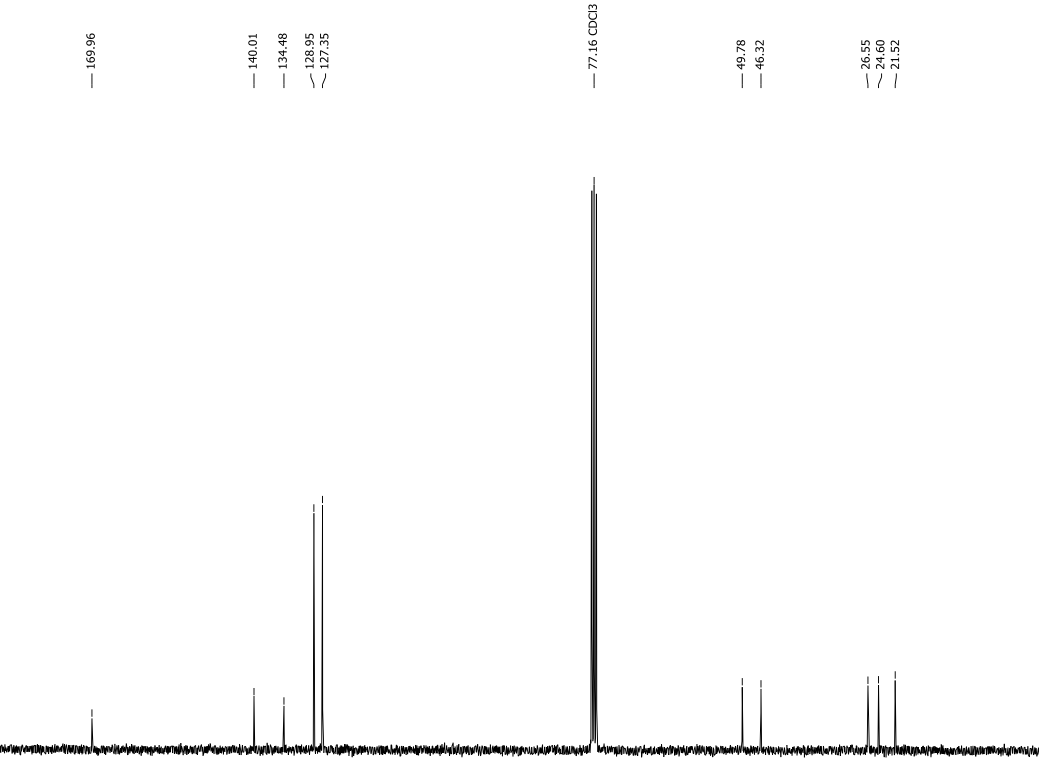

^{1}H NMR (300 MHz, Chloroform-d)

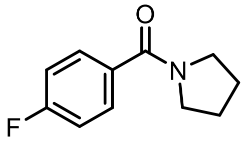

8B

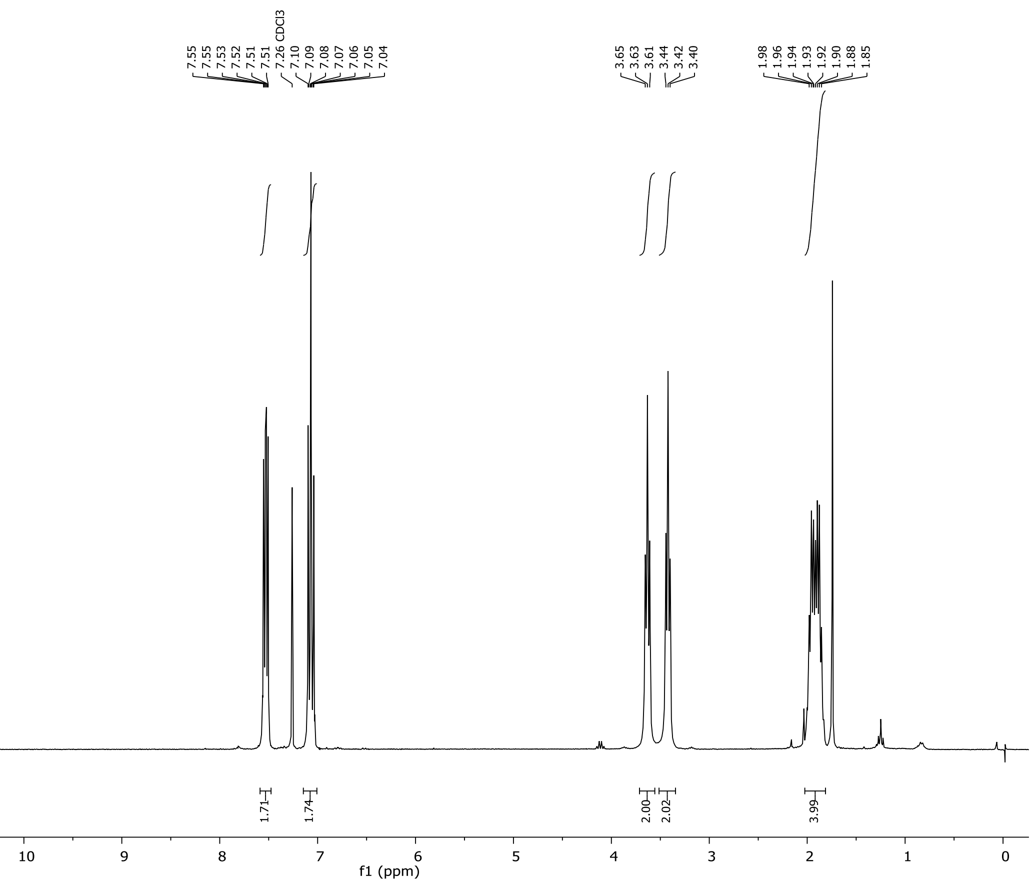

^{13}C NMR (75 MHz, Chloroform-d)

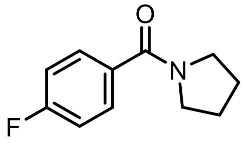

8B

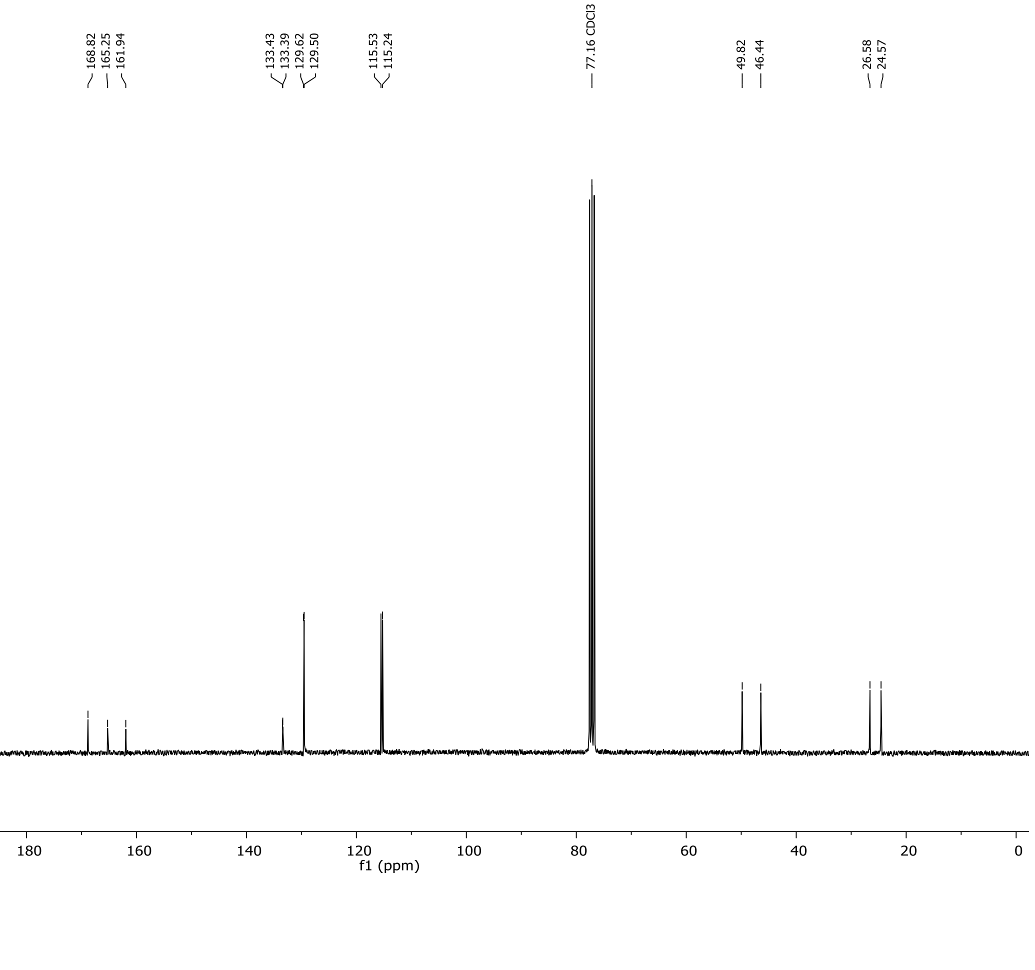

^{1}H NMR (400 MHz, Chloroform-d)

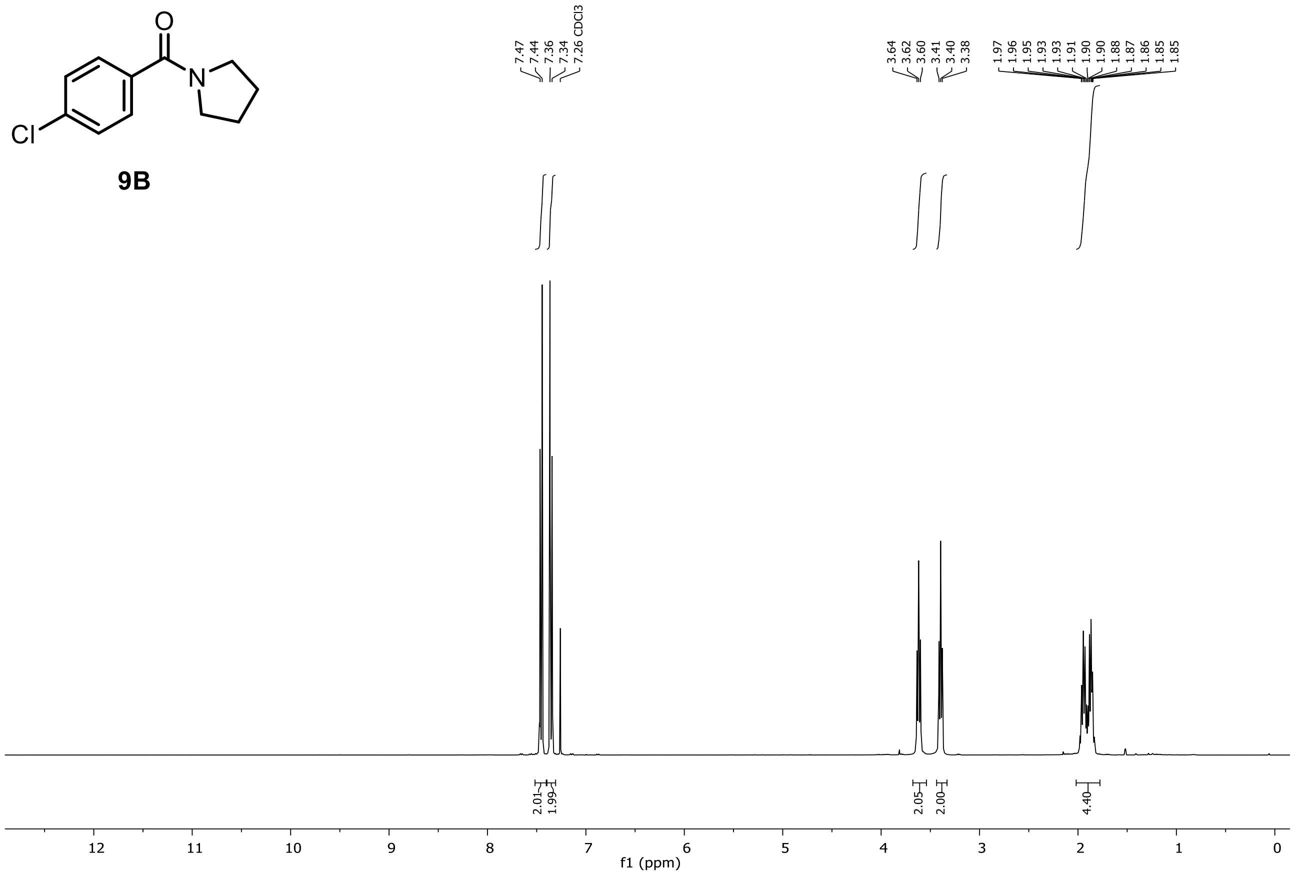

^{13}C NMR (100 MHz, Chloroform-d)

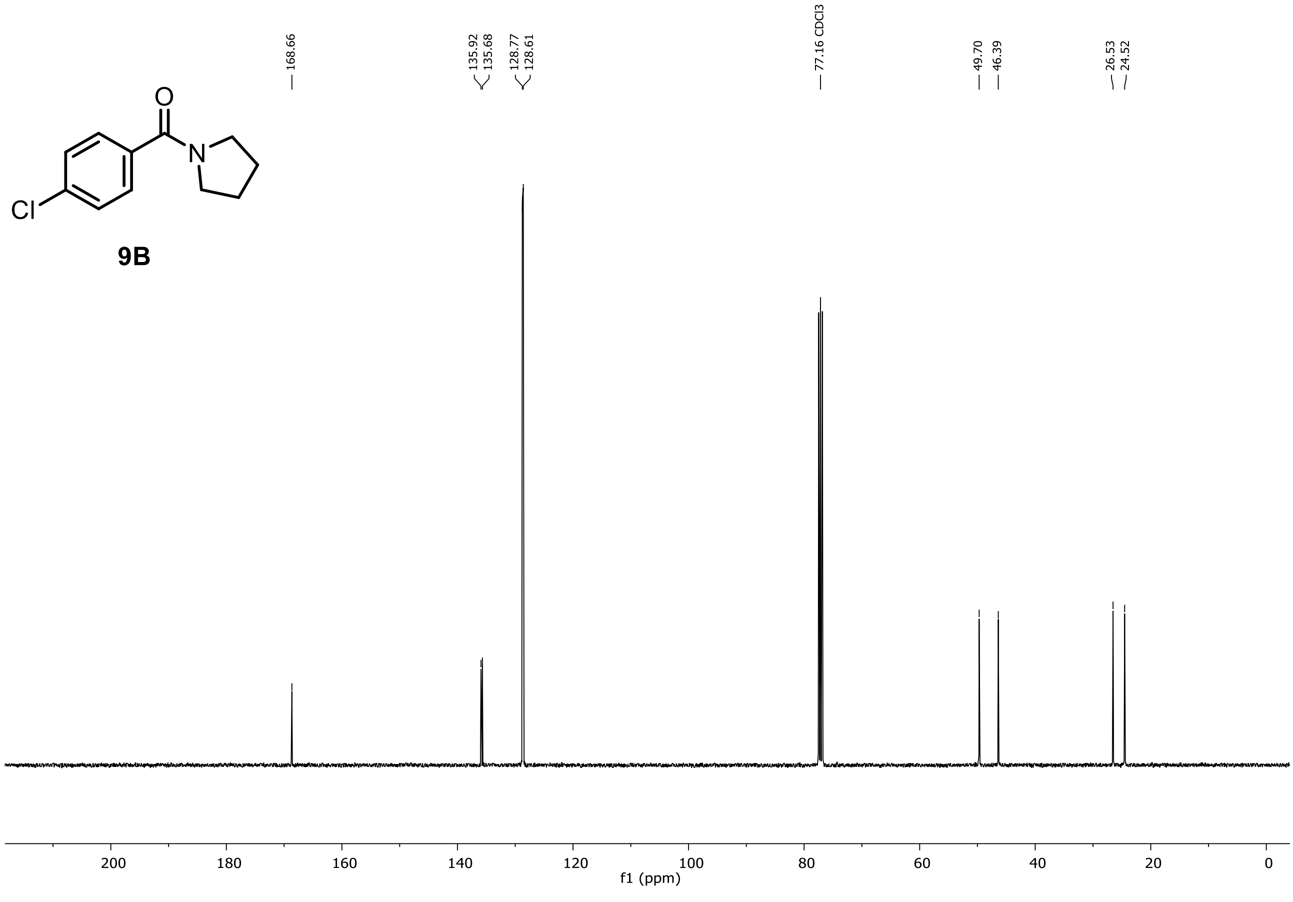

^{1}H NMR (300 MHz, Chloroform-d)

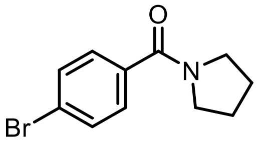

10B

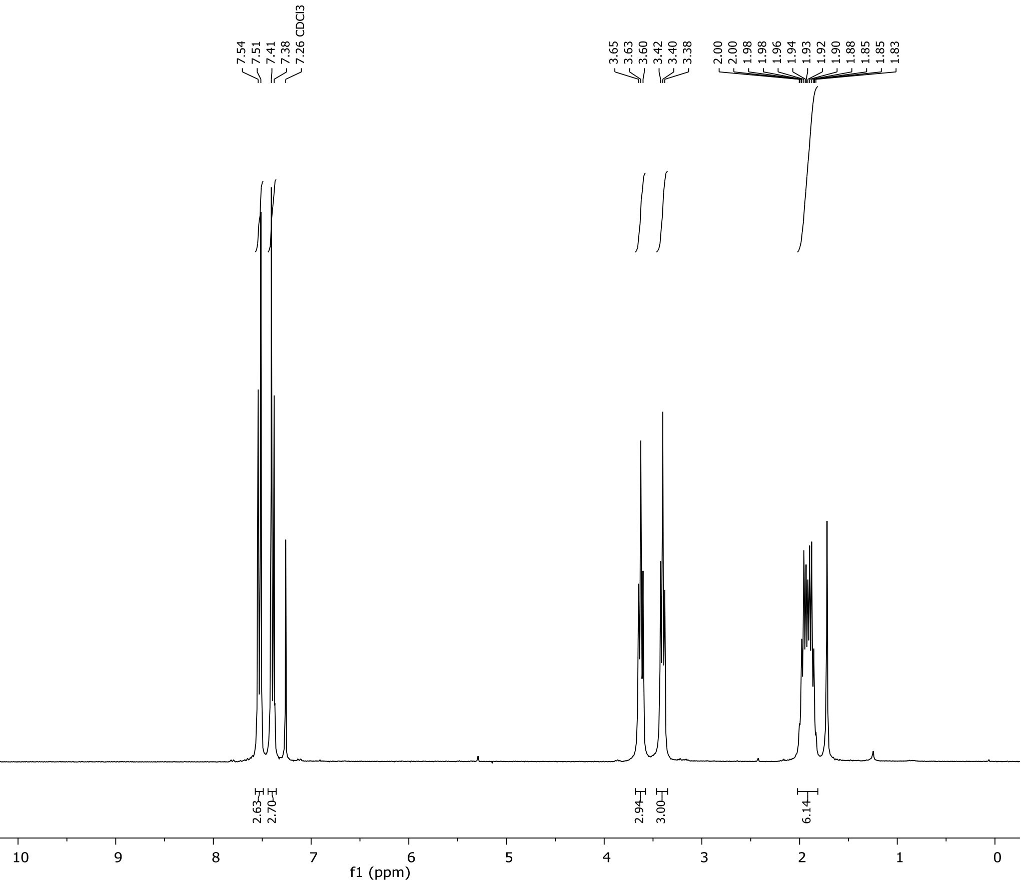

^{13}C NMR (75 MHz, Chloroform-d)

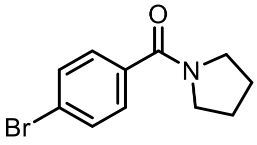

10B

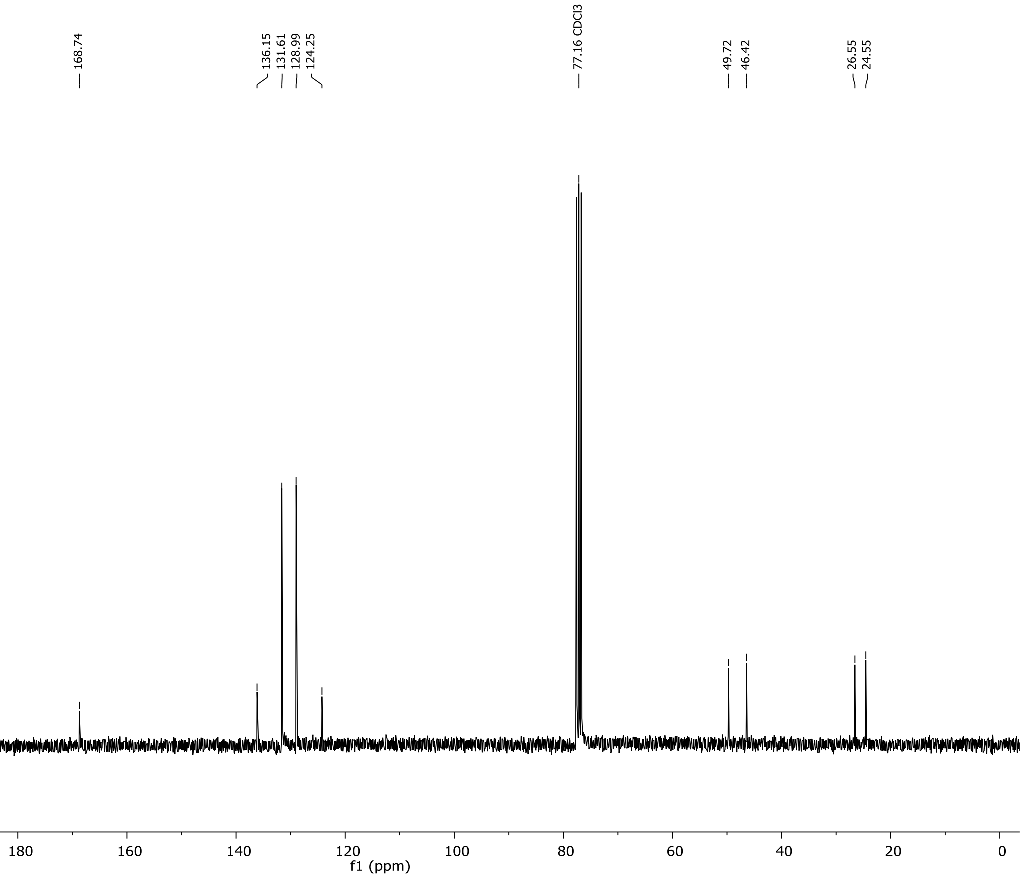

^{1}H NMR (300 MHz, Chloroform-d)

11B

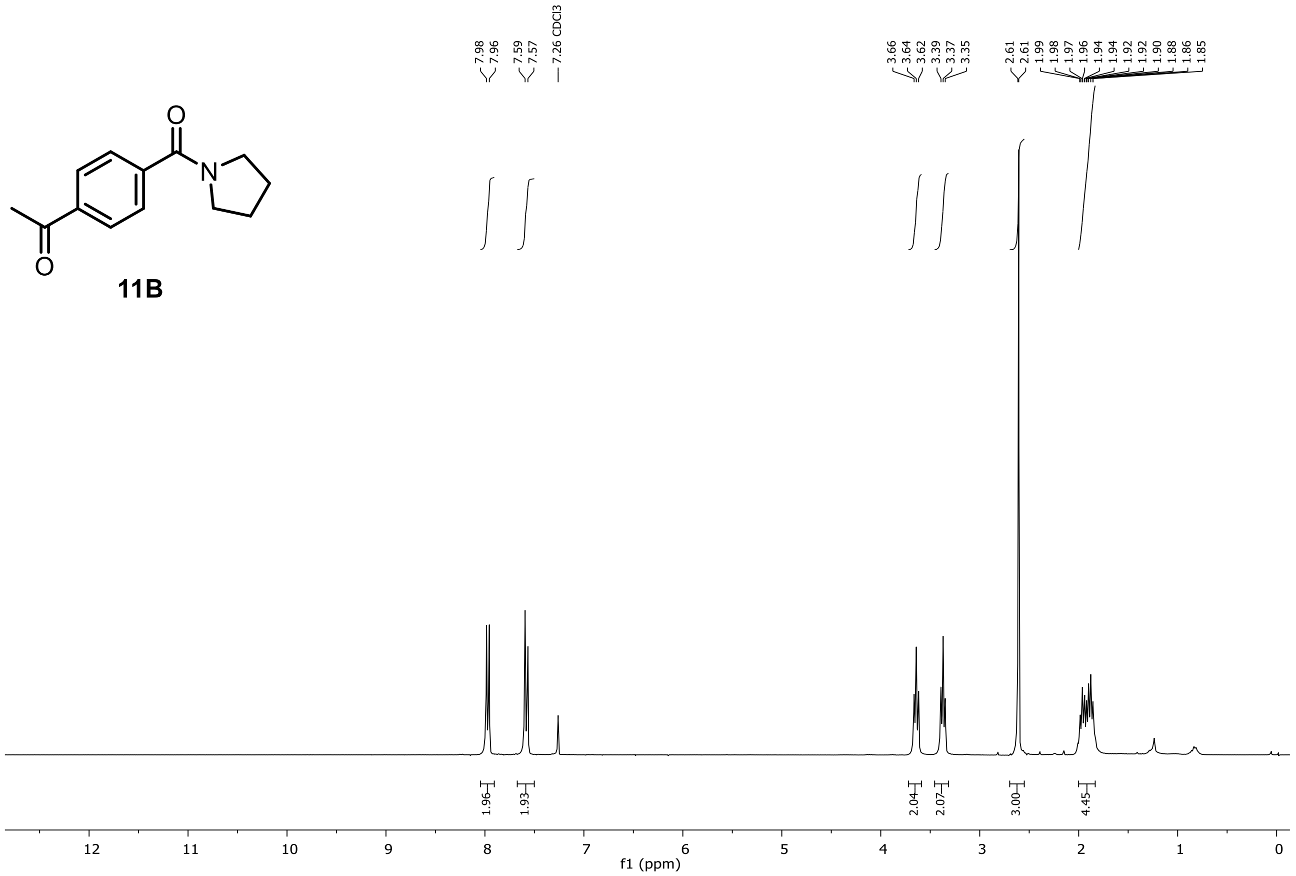

^{13}C NMR (75 MHz, Chloroform-d)

11B

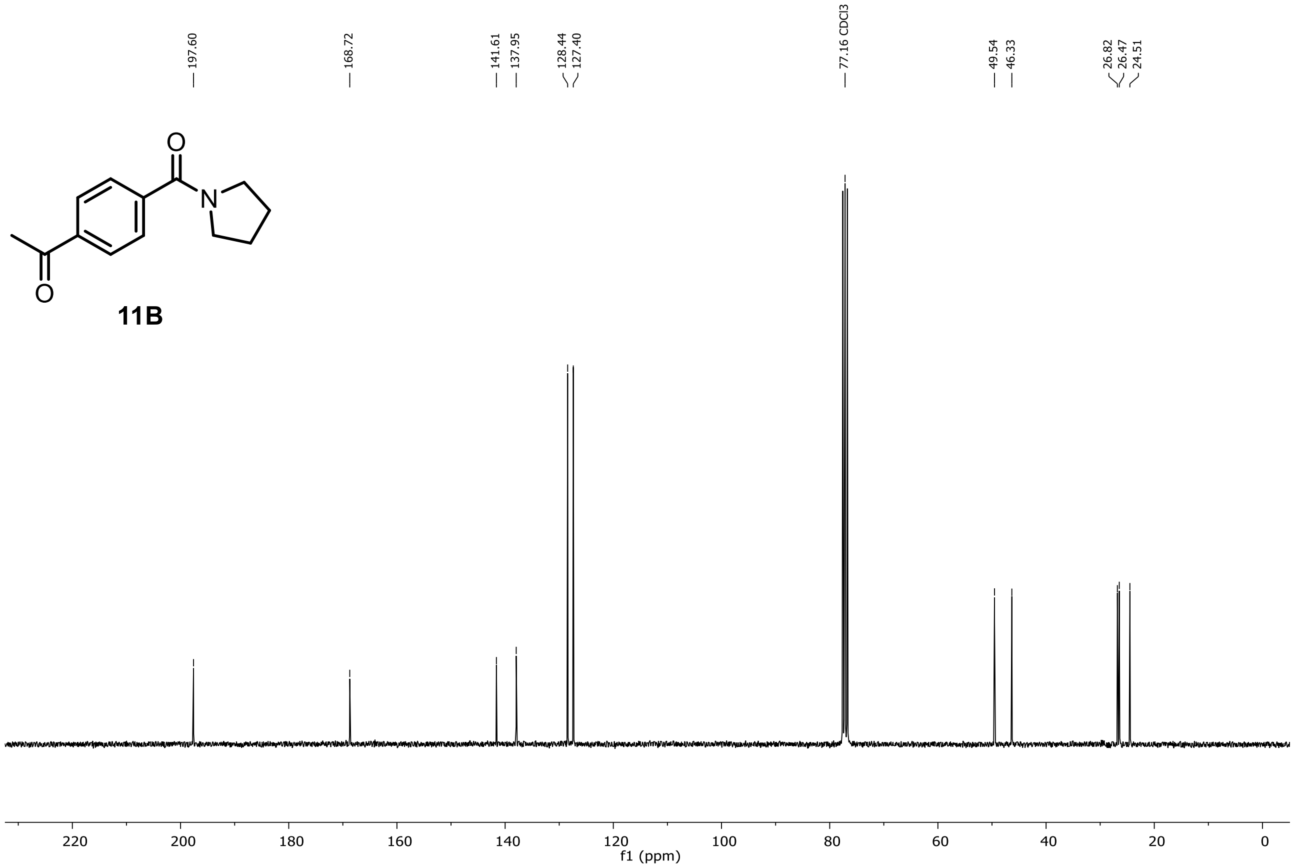

^{1}H NMR (300 MHz, Chloroform-d)

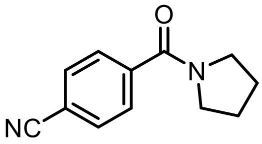

12B

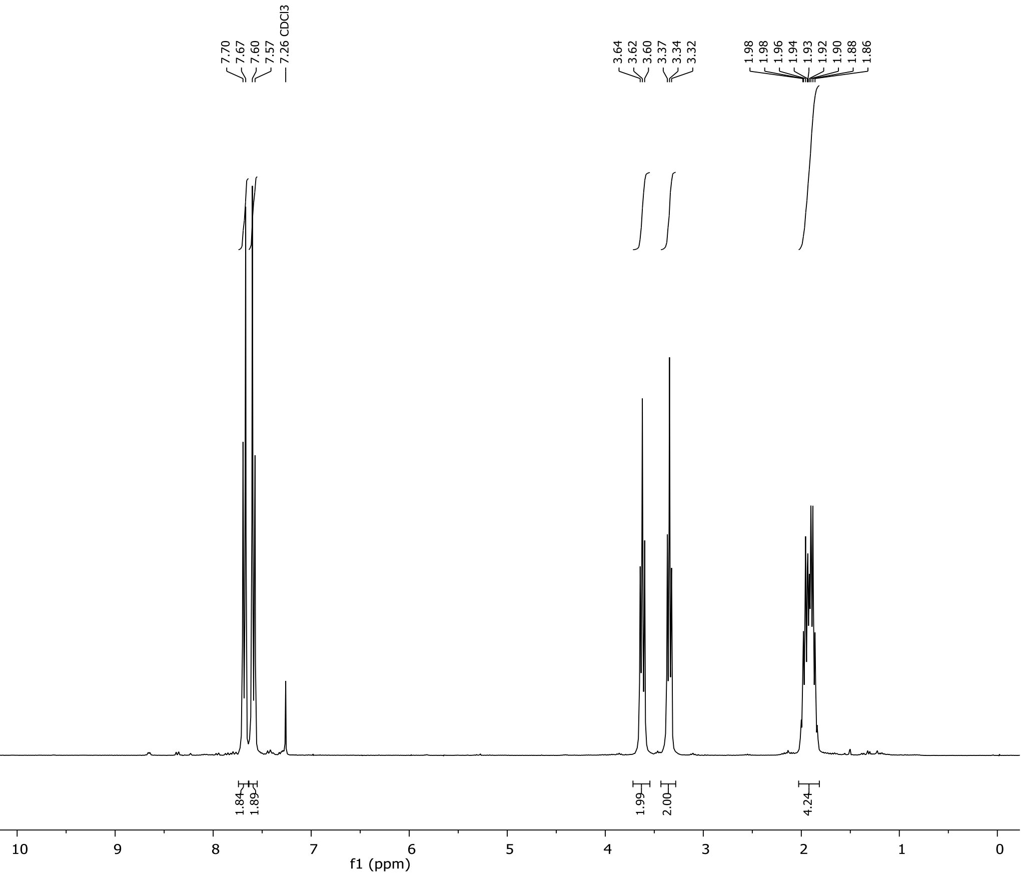

^{13}C NMR (75 MHz, Chloroform-d)

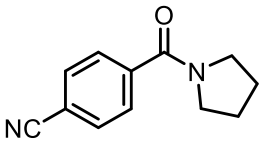

12B

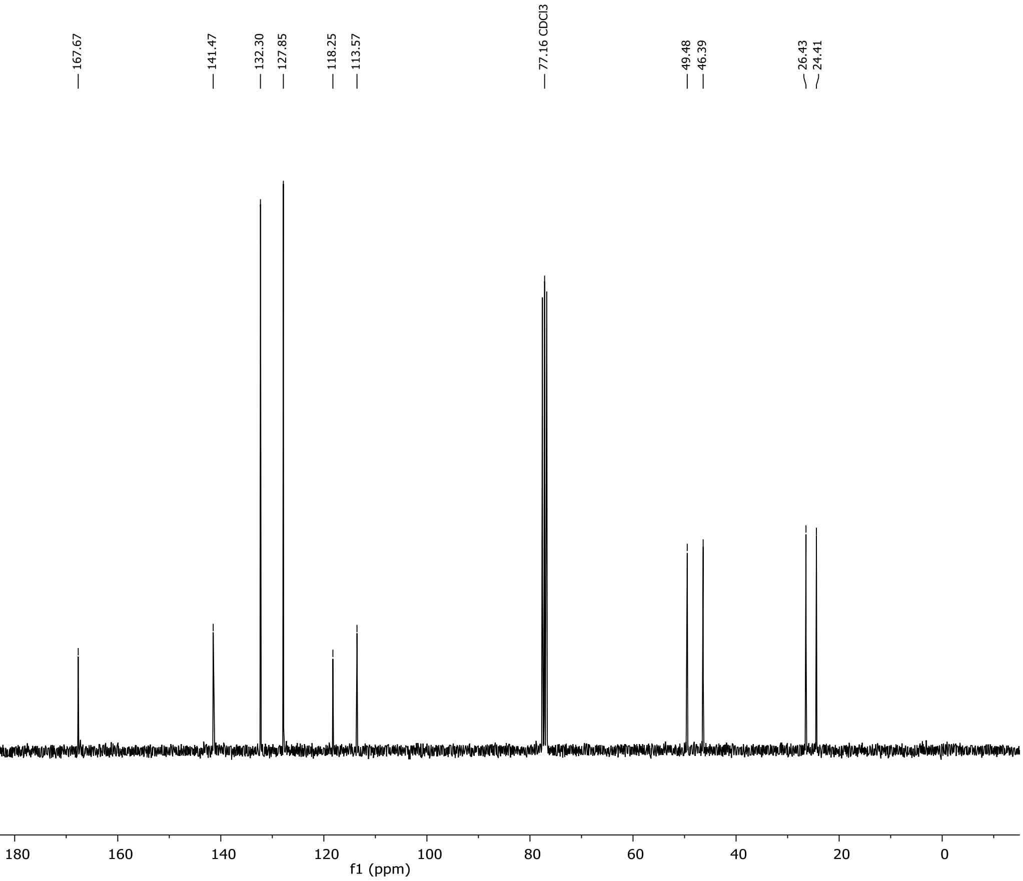

^{1}H NMR (300 MHz, Chloroform-d)

13B

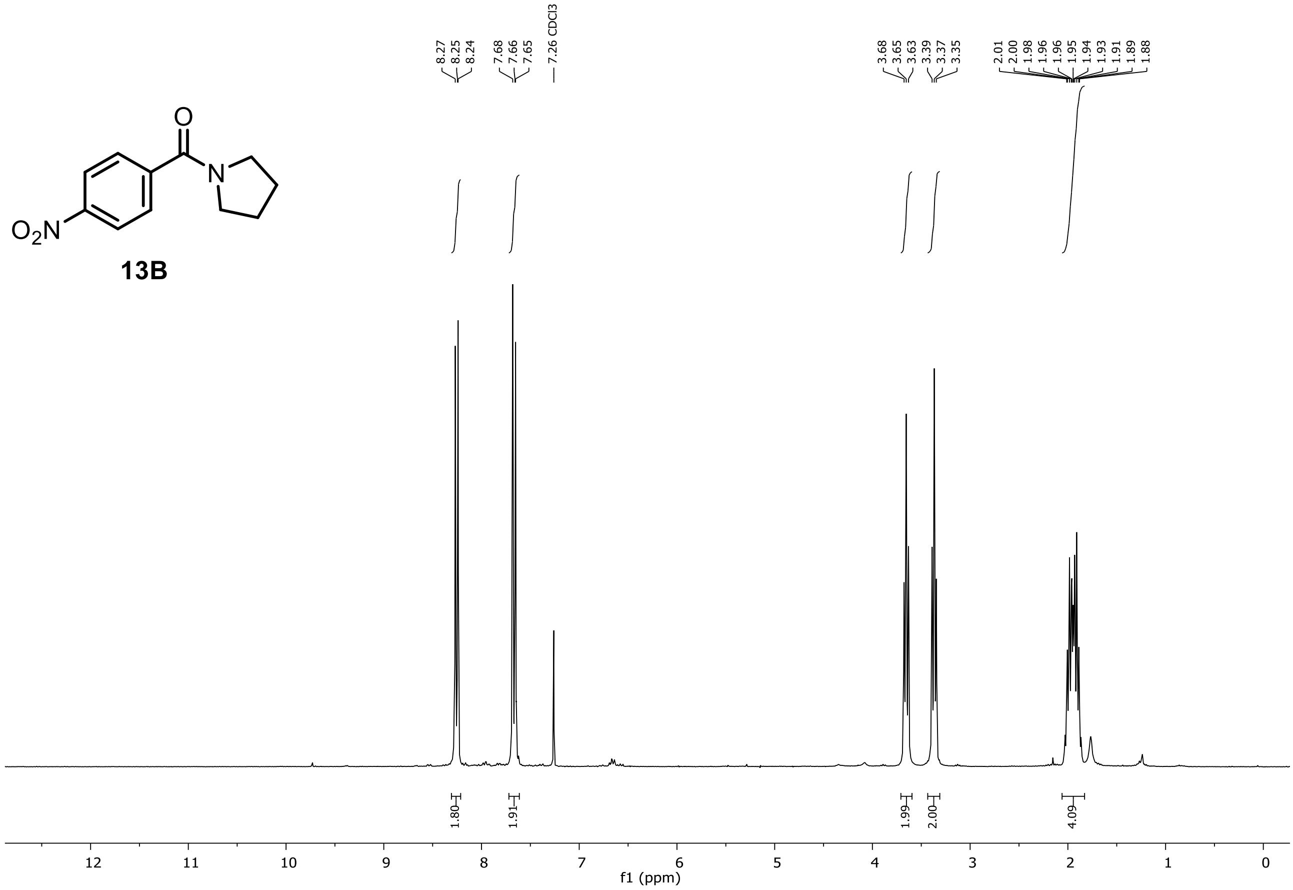

^{13}C NMR (75 MHz, Chloroform-d)

13B

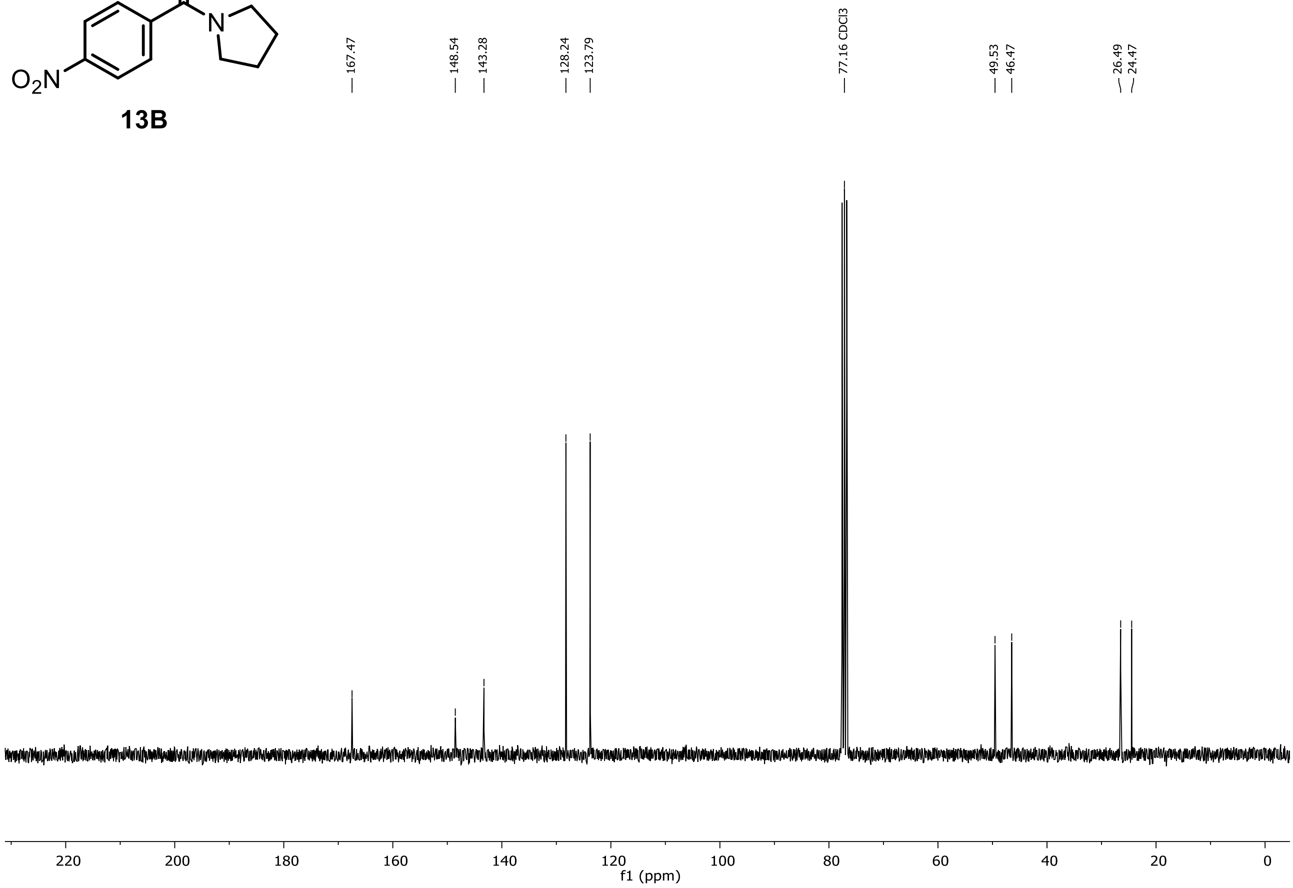

^1H NMR (300 MHz, Chloroform-d)

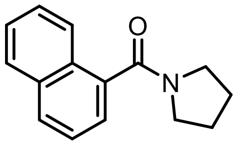

14B

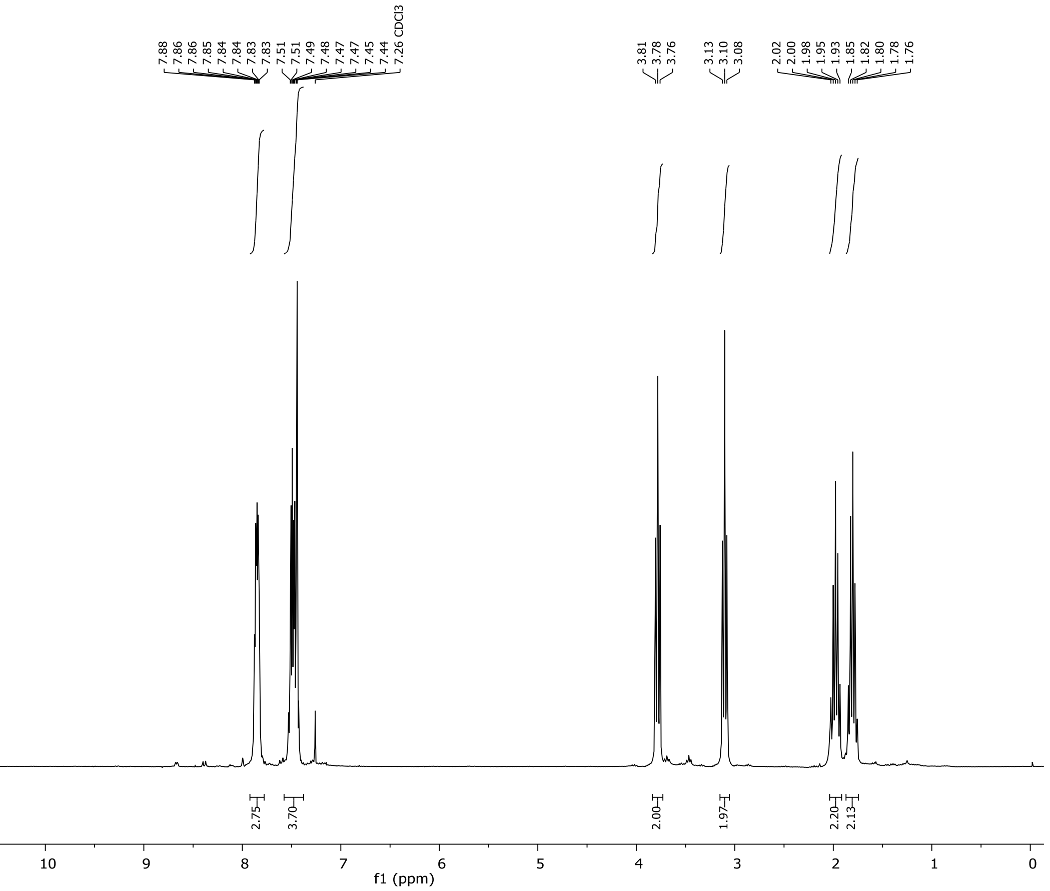

^{13}C NMR (75 MHz, Chloroform-d)

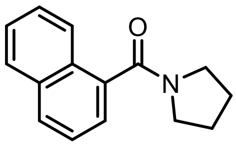

14B

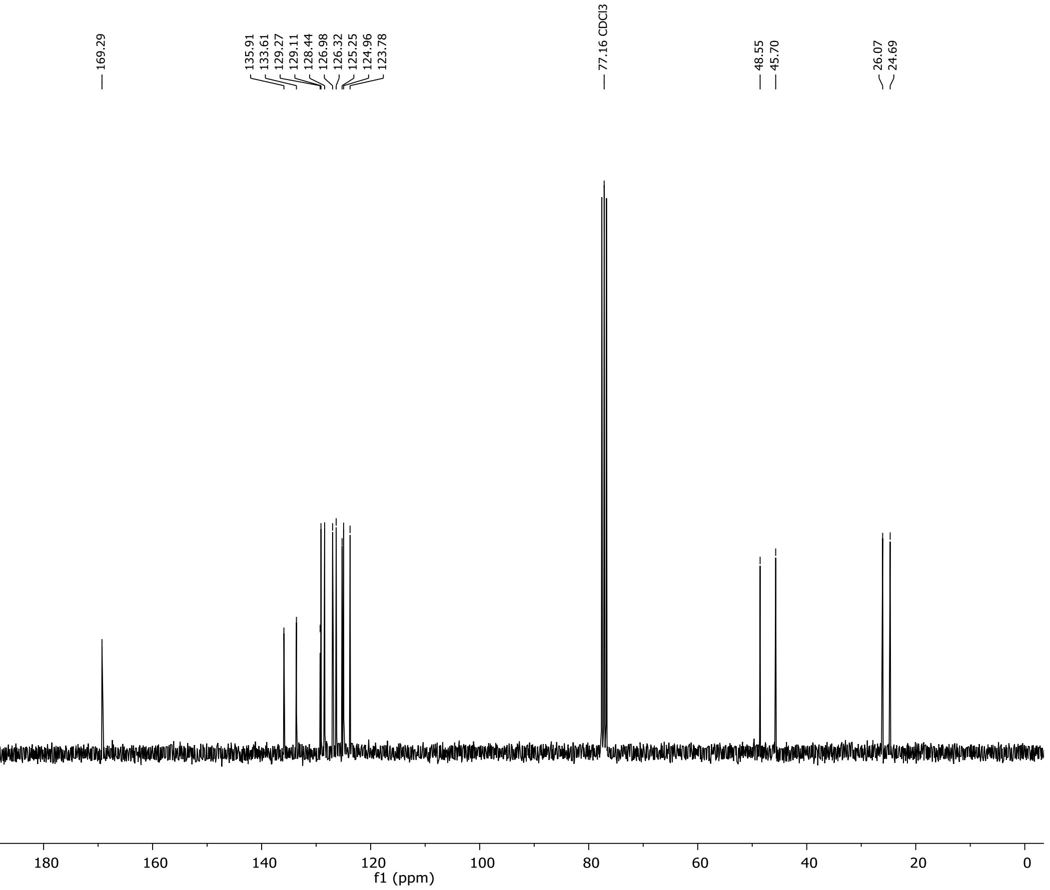

^{1}H NMR (300 MHz, Chloroform-d)

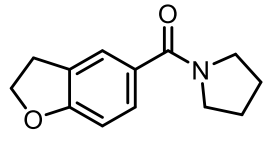

15B

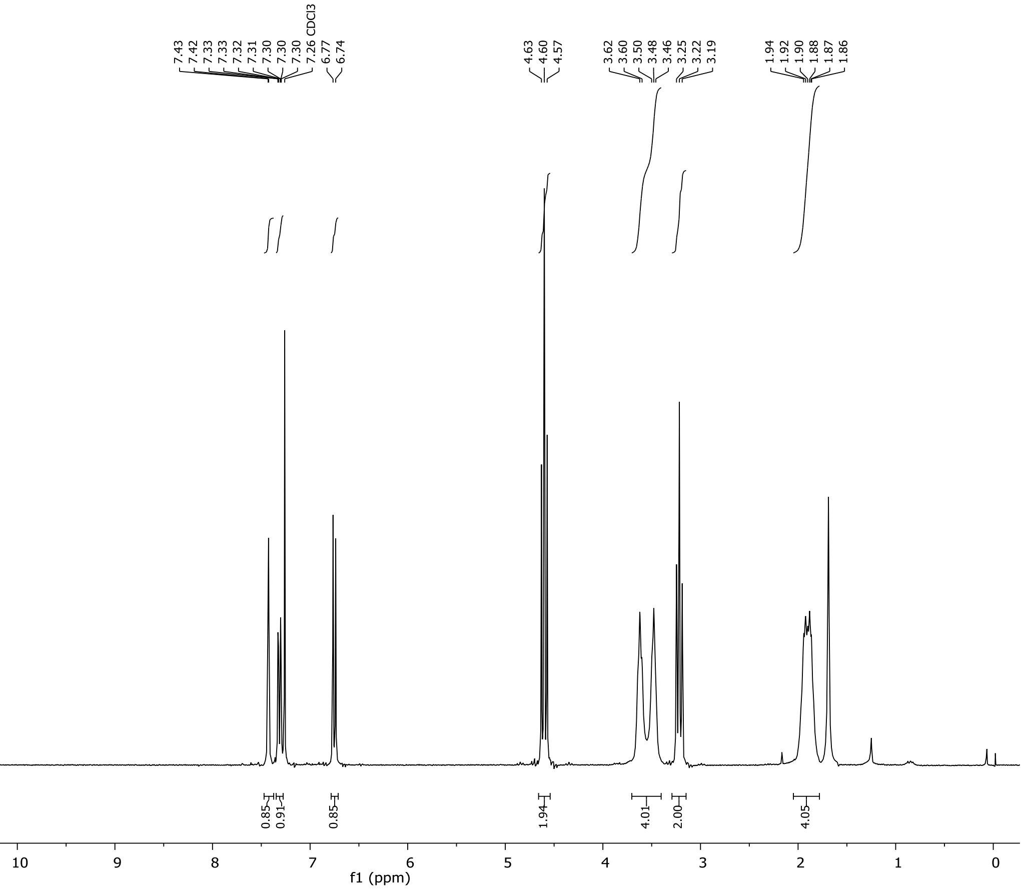

^{13}C NMR (75 MHz, Chloroform-d)

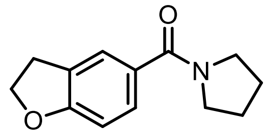

15B

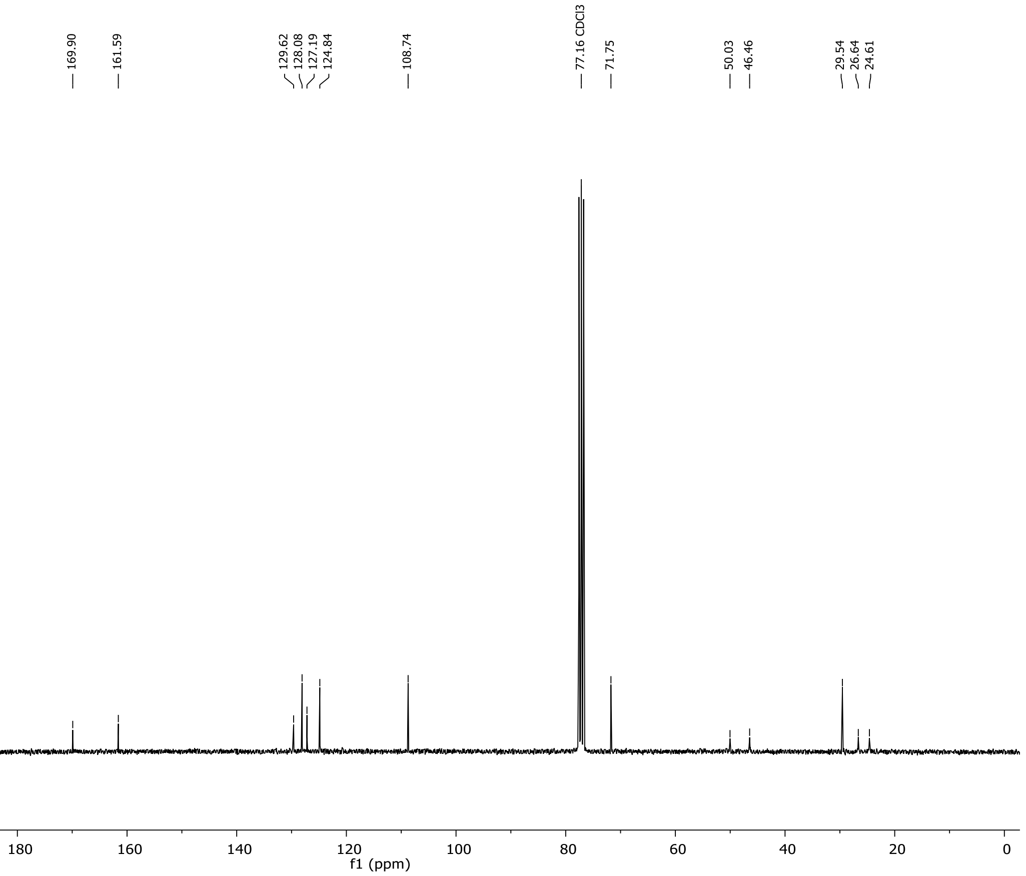

169.90
161.59
129.62
128.08
127.19
124.84
108.74
77.16 CDCl3
71.75
50.03
46.46
29.54
26.64
24.61
220
200
180
160
140
120
100
80
60
40
20
0
f1 (ppm)

^{1}H NMR (300 MHz, Chloroform-d)

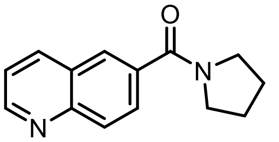

16B

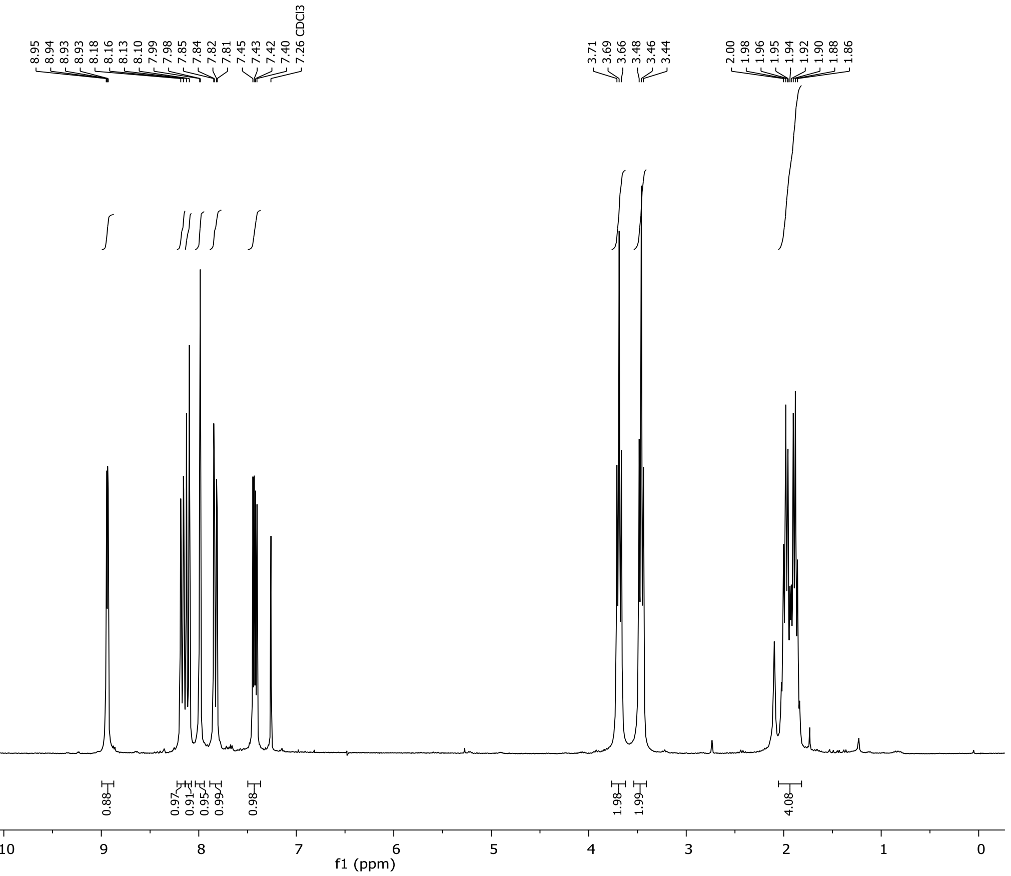

^{13}C NMR (75 MHz, Chloroform-d)

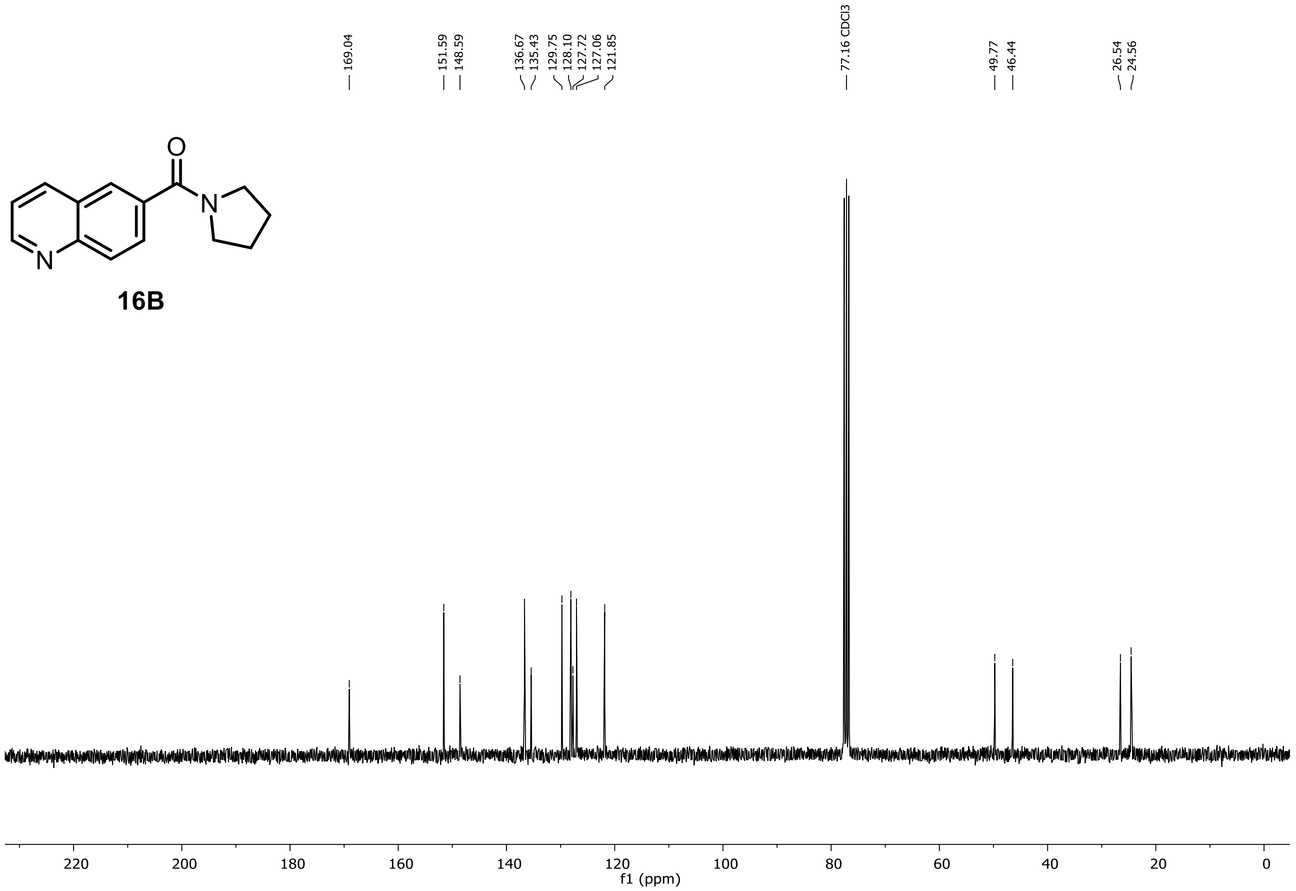
169.04
151.59
148.59
136.67
135.43
129.75
128.10
127.72
127.06
121.85
77.16 CDCl3
49.77
46.44
26.54
24.56
O
N
N
220
200
180
160
140
120
100
80
60
40
20
0
f1 (ppm)

16B

^{1}H NMR (300 MHz, Chloroform-d)

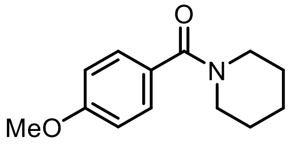

17B

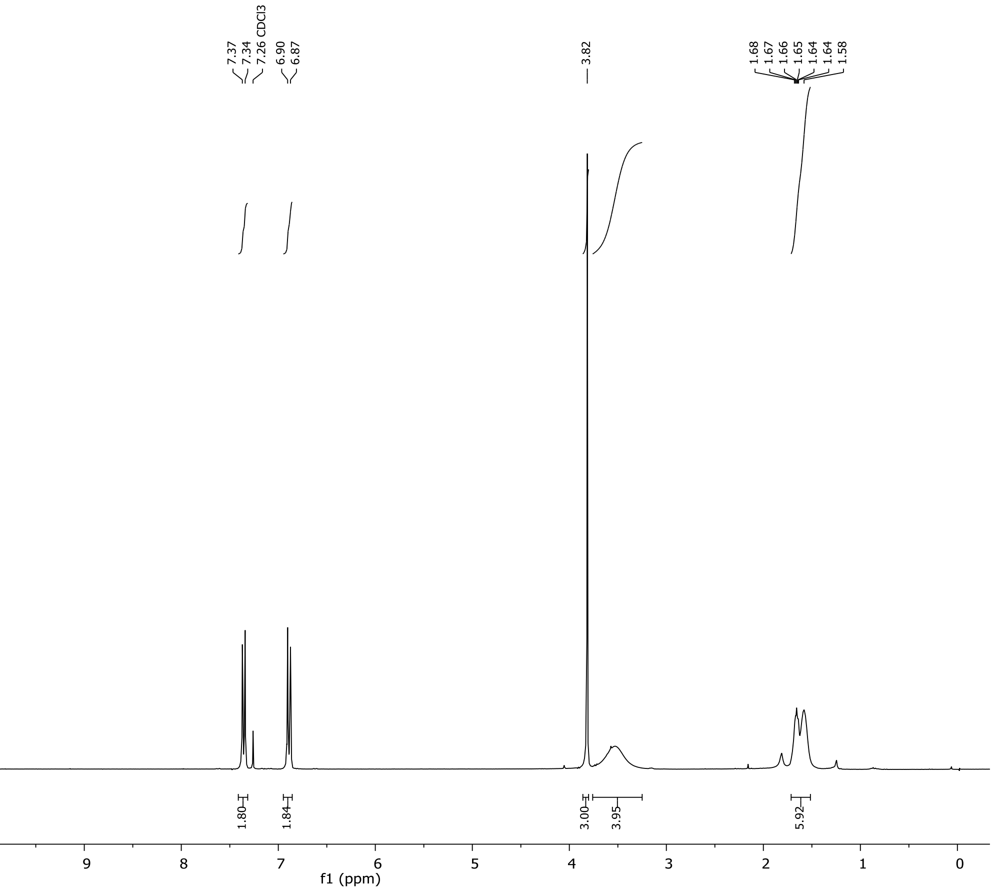

^{13}C NMR (75 MHz, Chloroform-d)

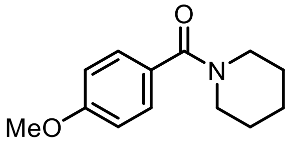

17B

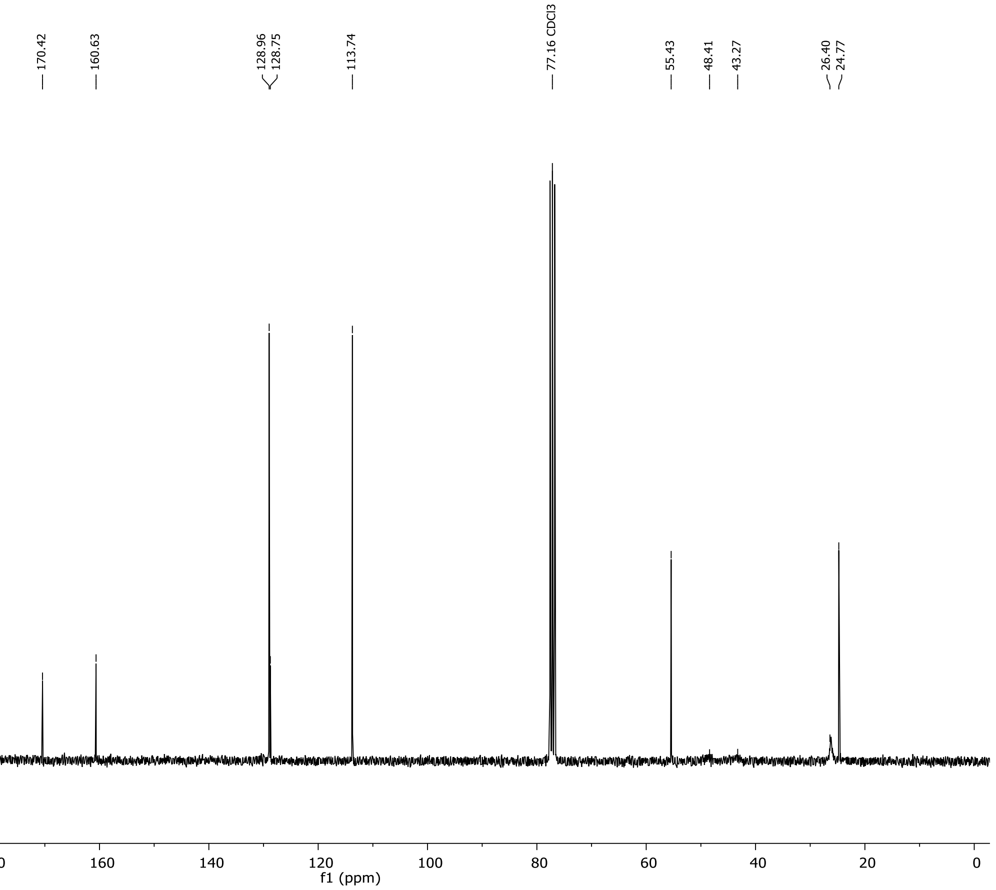

^{1}H NMR (300 MHz, Chloroform-d)

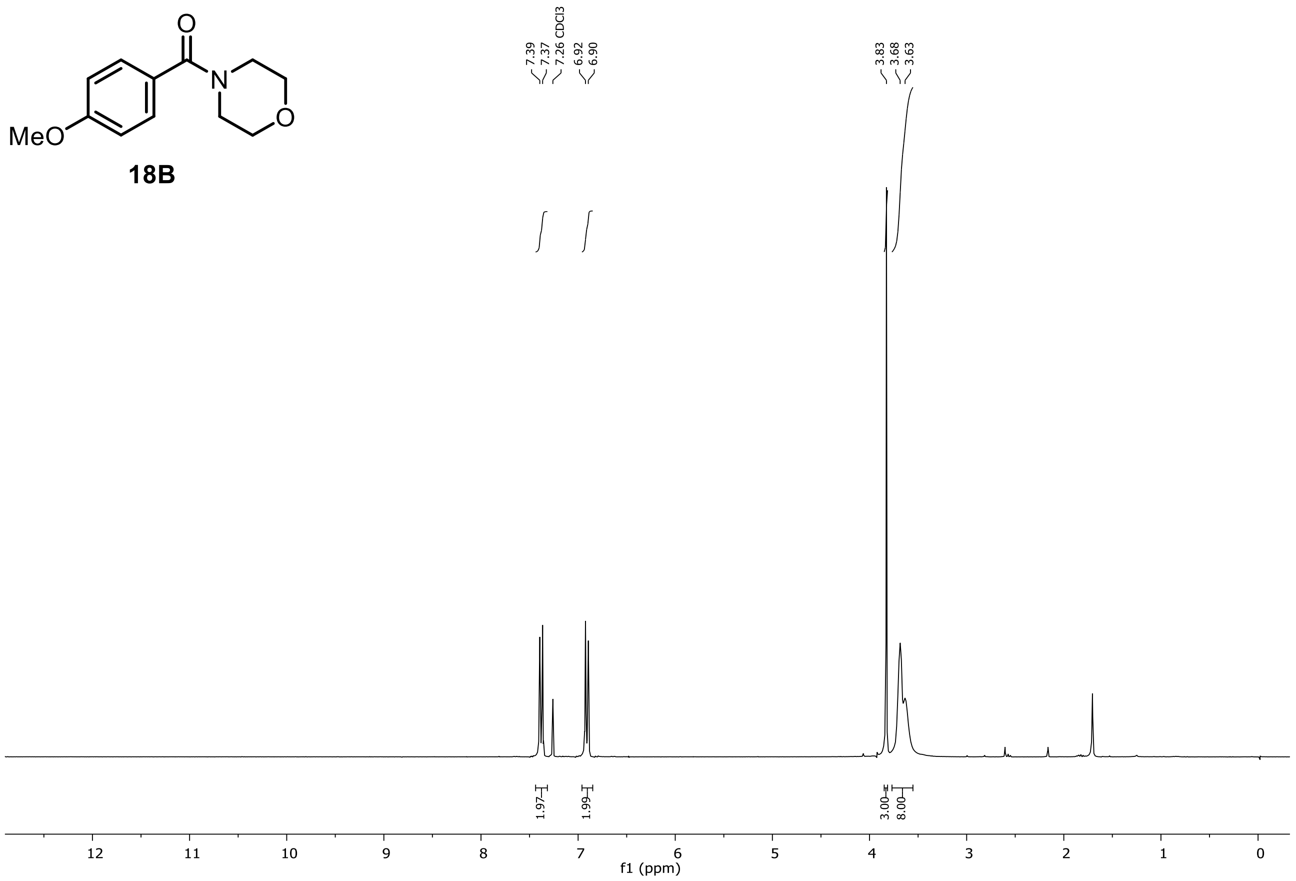

^{13}C NMR (75 MHz, Chloroform-d)

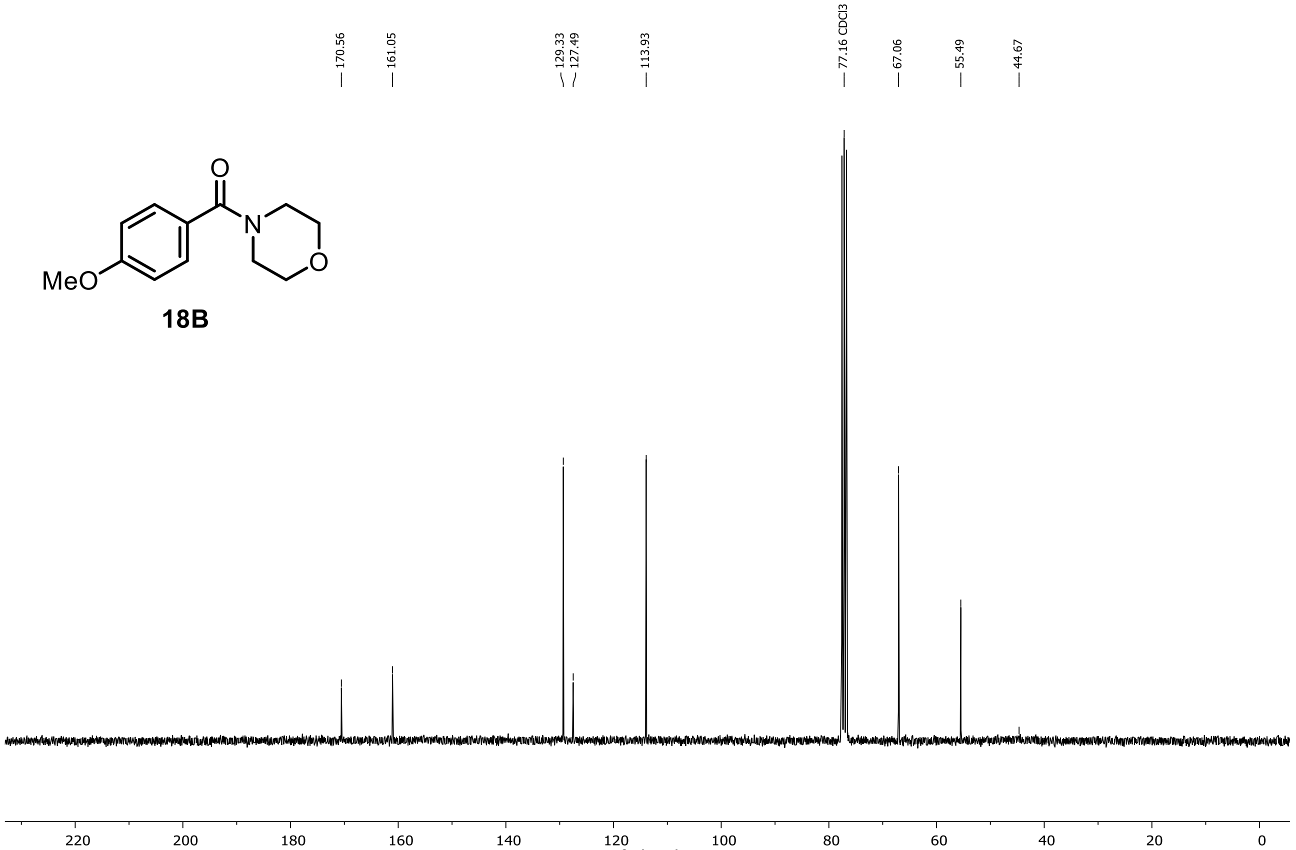

^1H NMR (300 MHz, Chloroform-d)

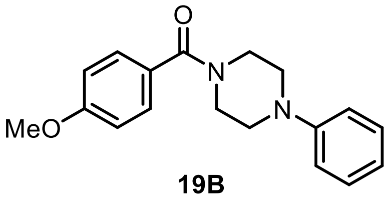

19B

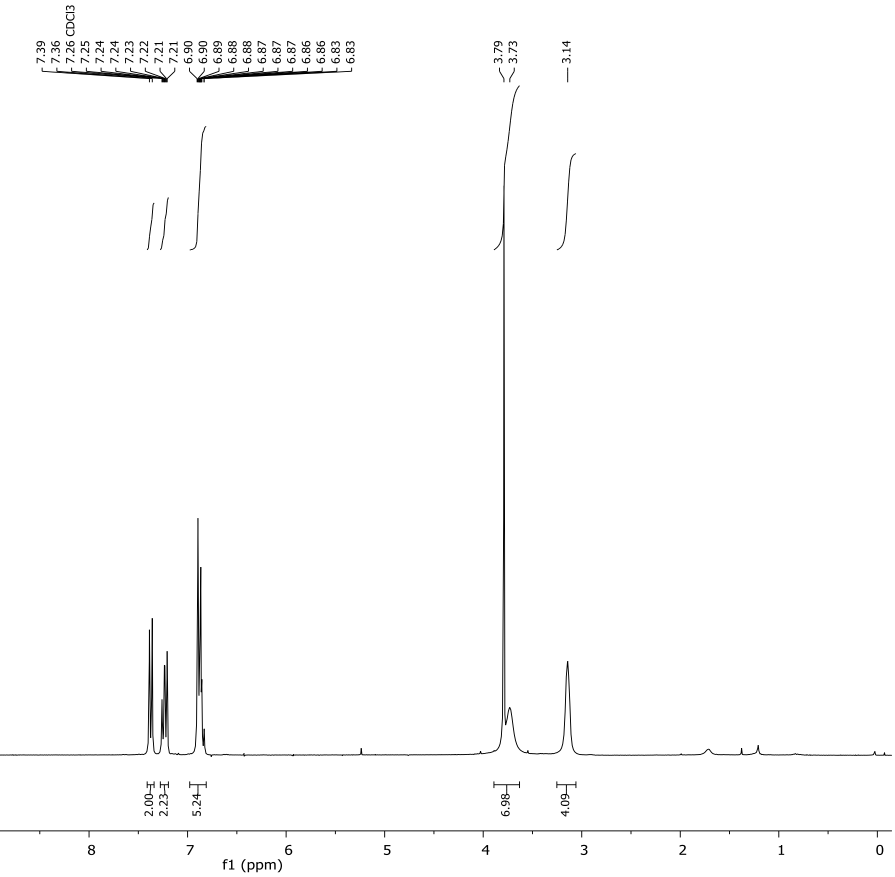

^{13}C NMR (75 MHz, Chloroform-d)

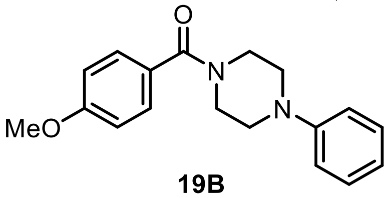

19B

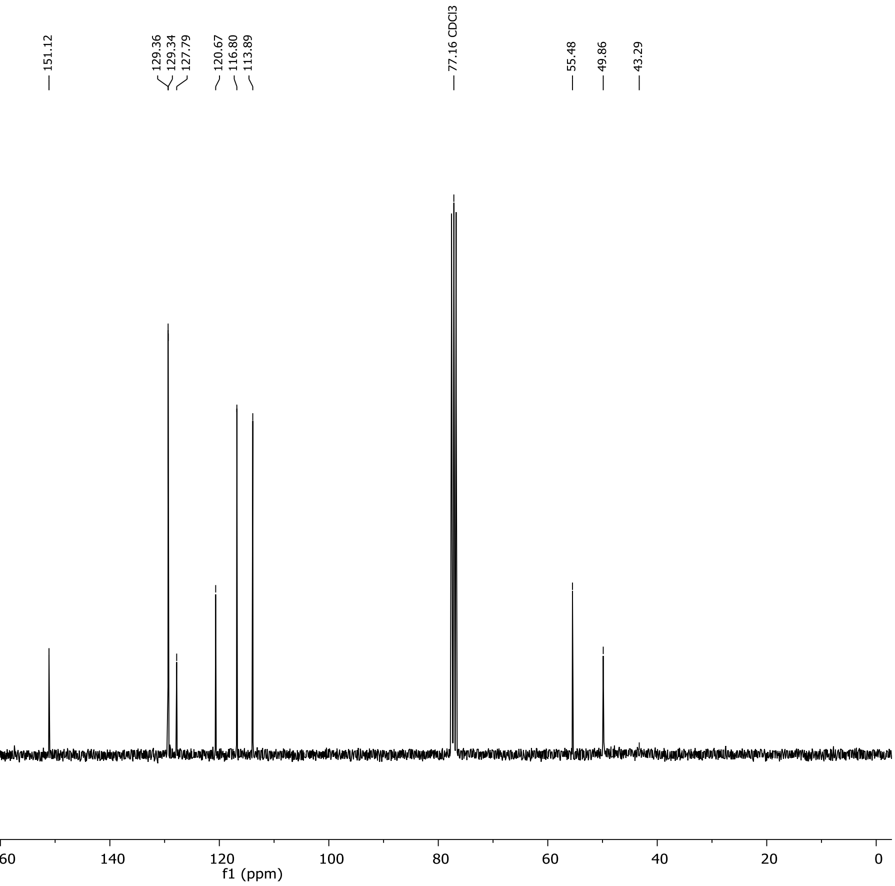

^{1}H NMR (300 MHz, Chloroform-d)

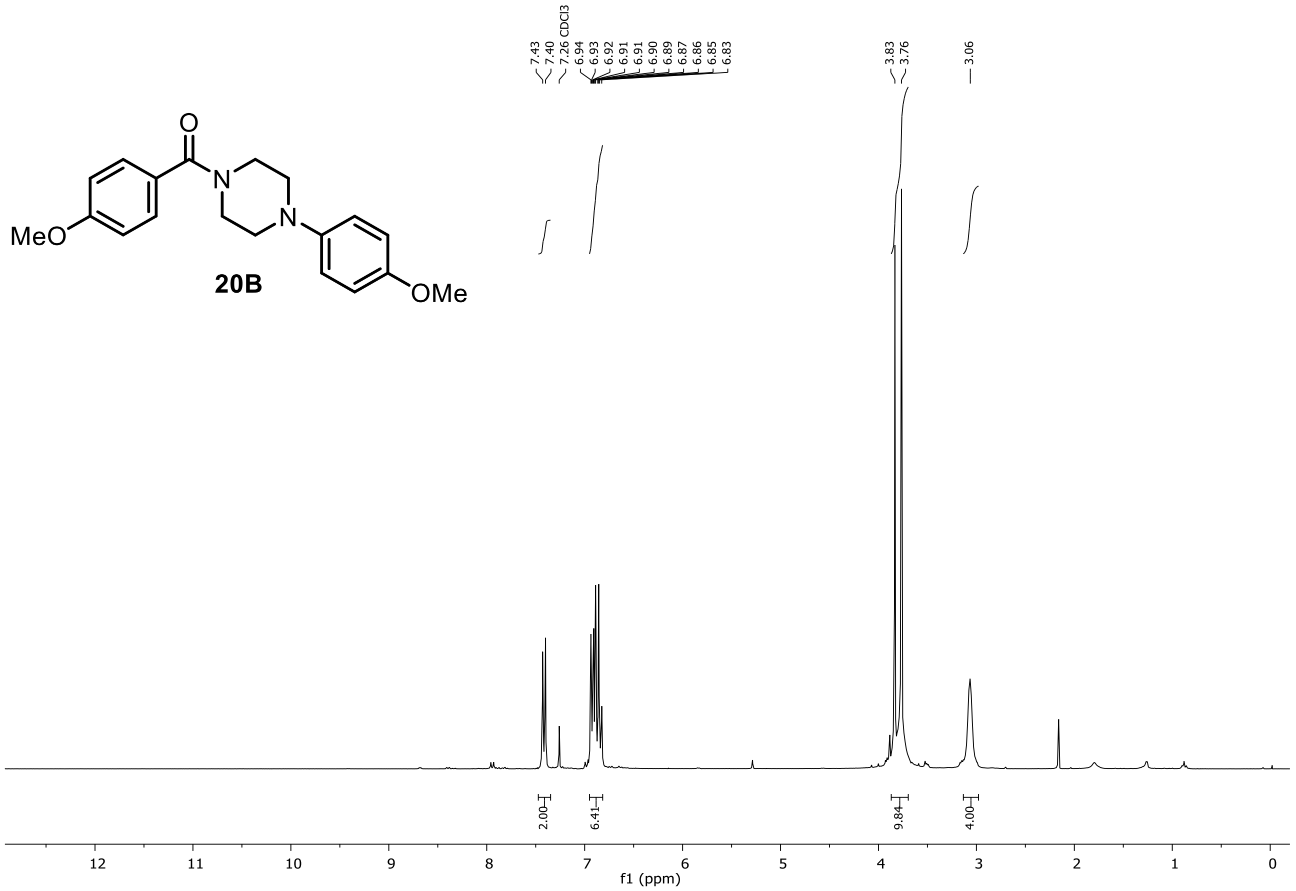

^{13}C NMR (75 MHz, Chloroform-d)

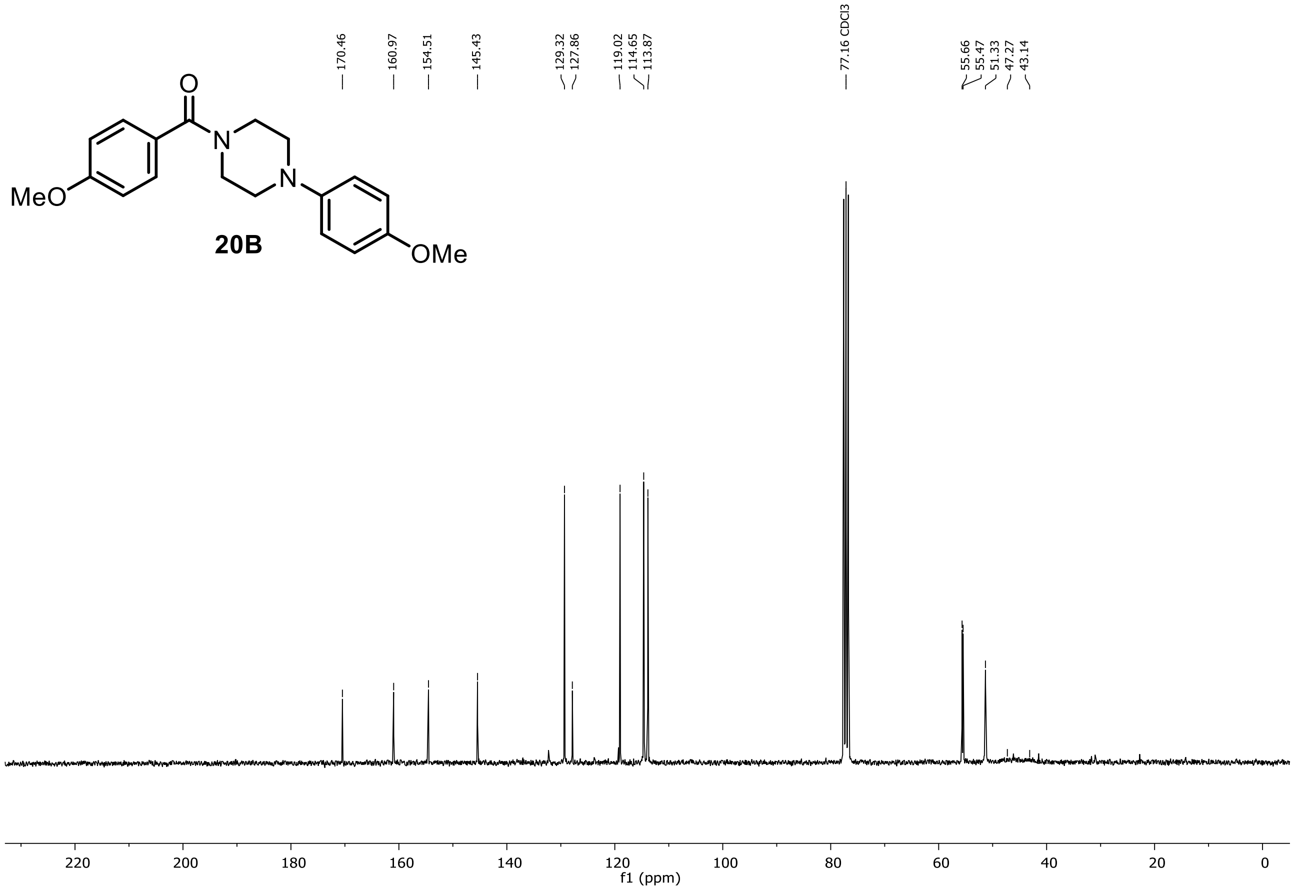

^{1}H NMR (300 MHz, Chloroform-d)

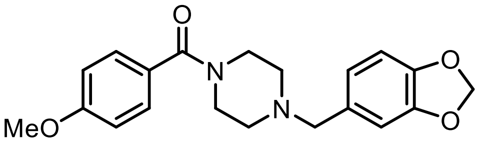

21B

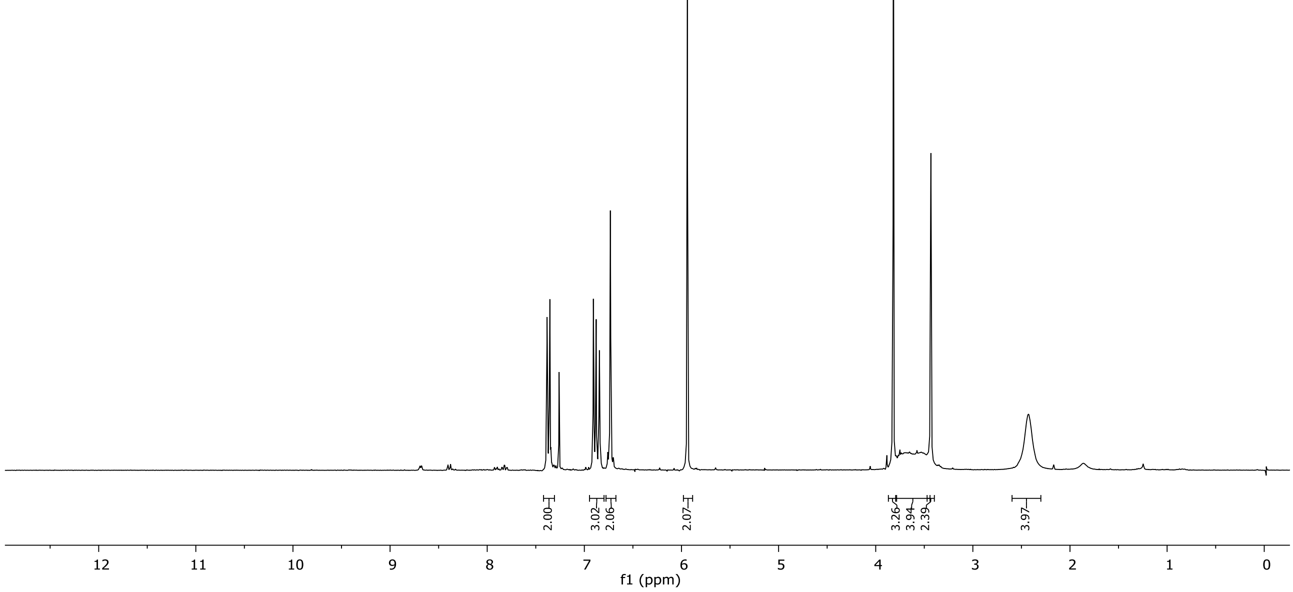

^{13}C NMR (75 MHz, Chloroform-d)

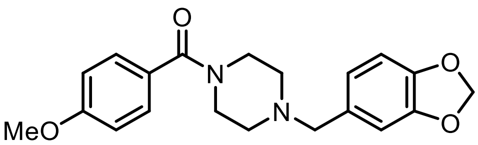

21B

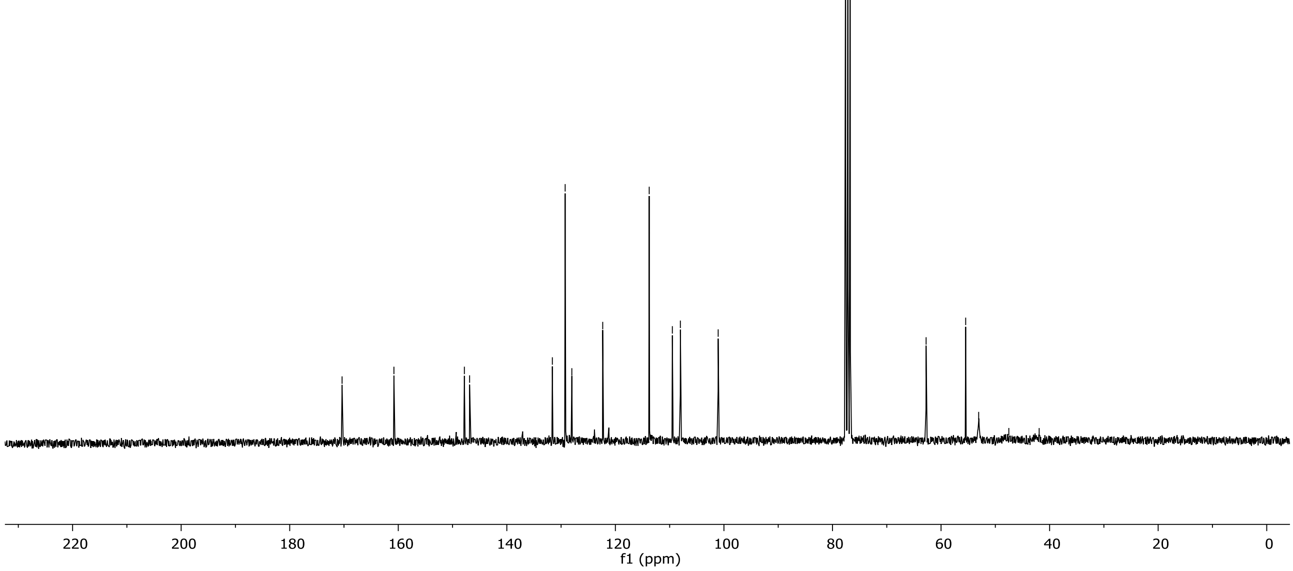

^{1}H NMR (300 MHz, Chloroform-d)

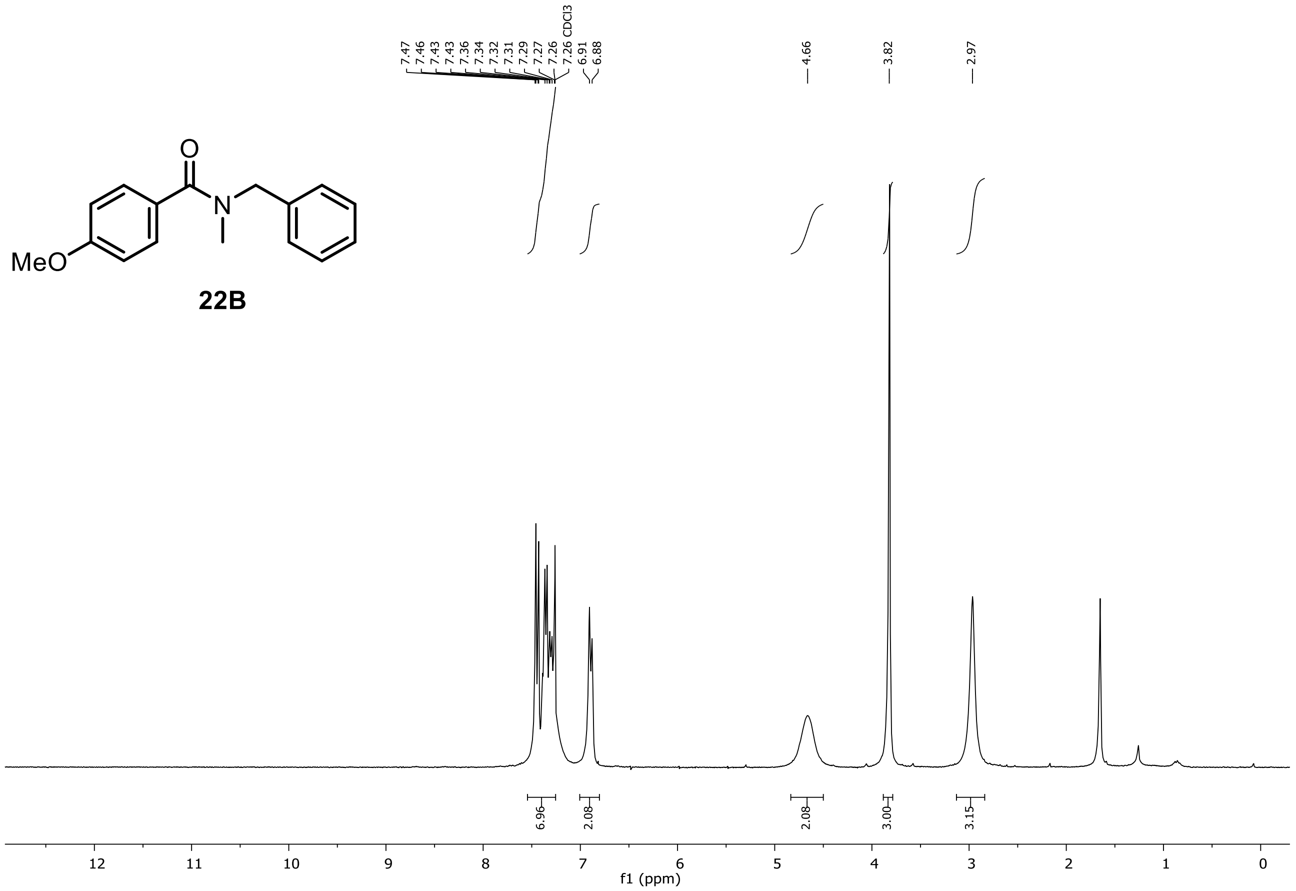

^{13}C NMR (75 MHz, Chloroform-d)

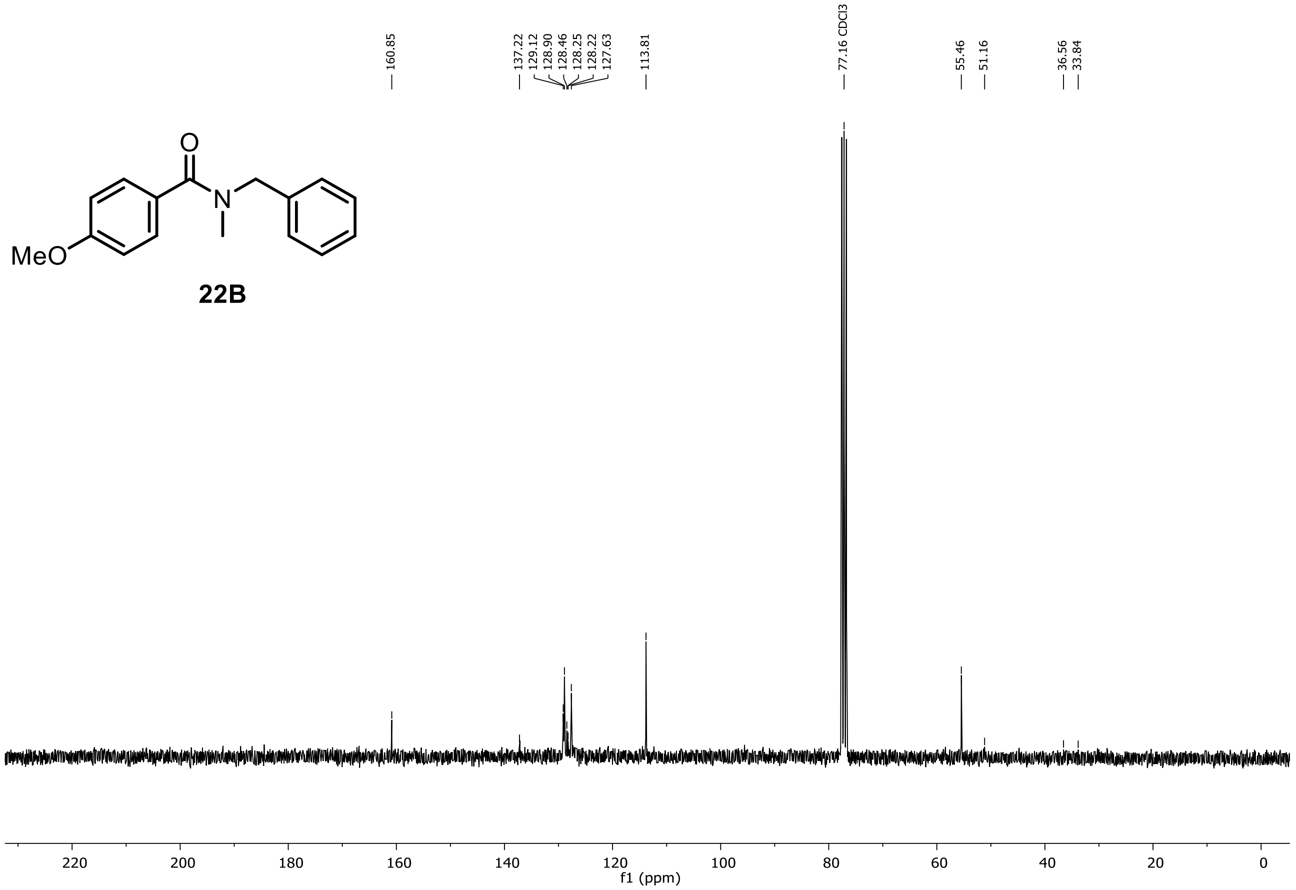

^1H NMR (300 MHz, Chloroform-d)

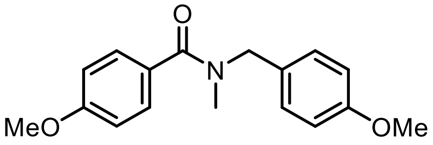

23B

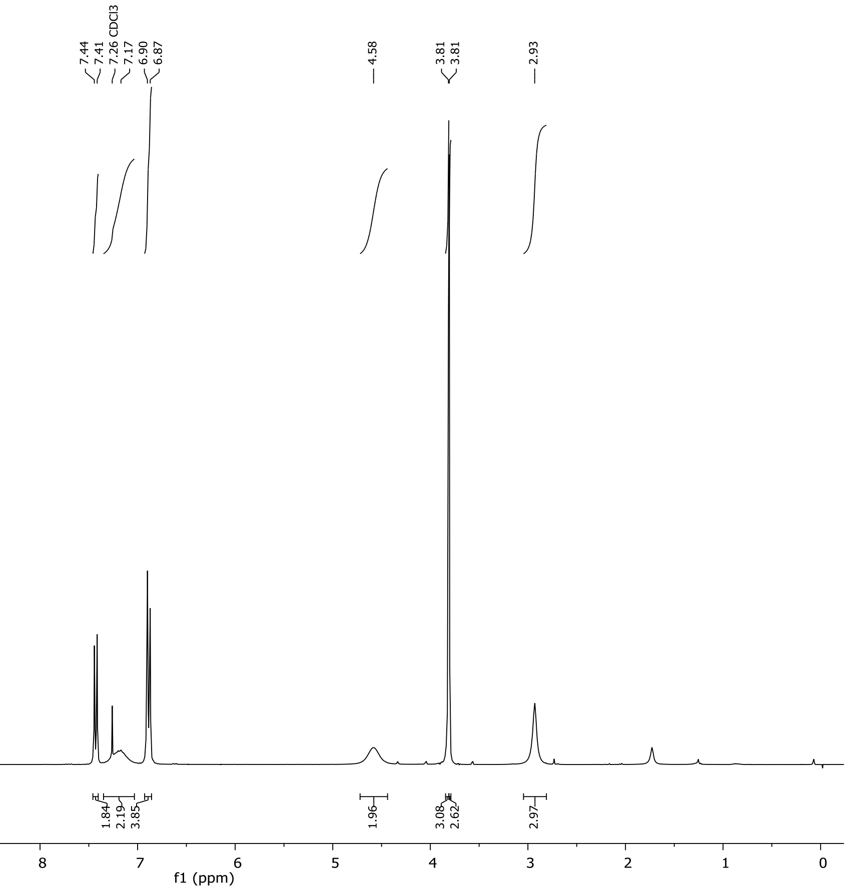

^{13}C NMR (75 MHz, Chloroform-d)

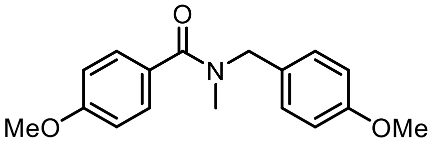

23B

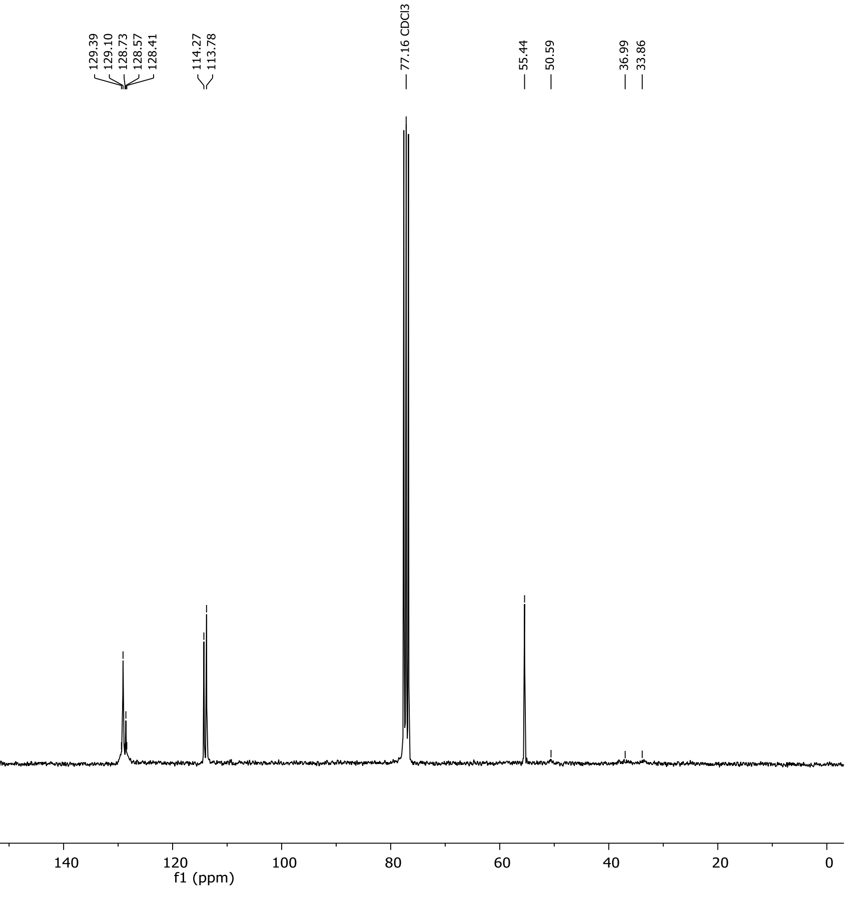

^{1}H NMR (300 MHz, Chloroform-d)

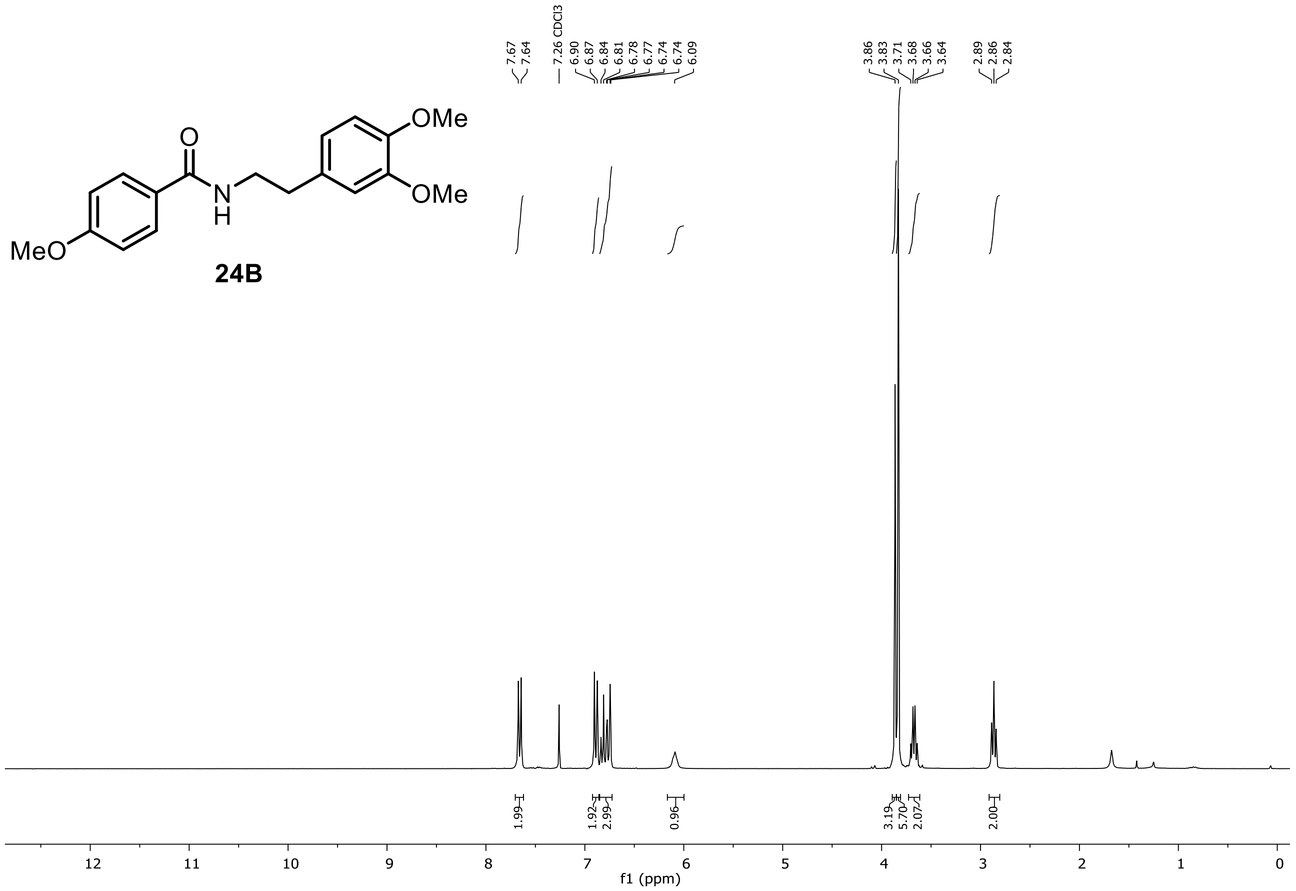

^{13}C NMR (75 MHz, Chloroform-d)

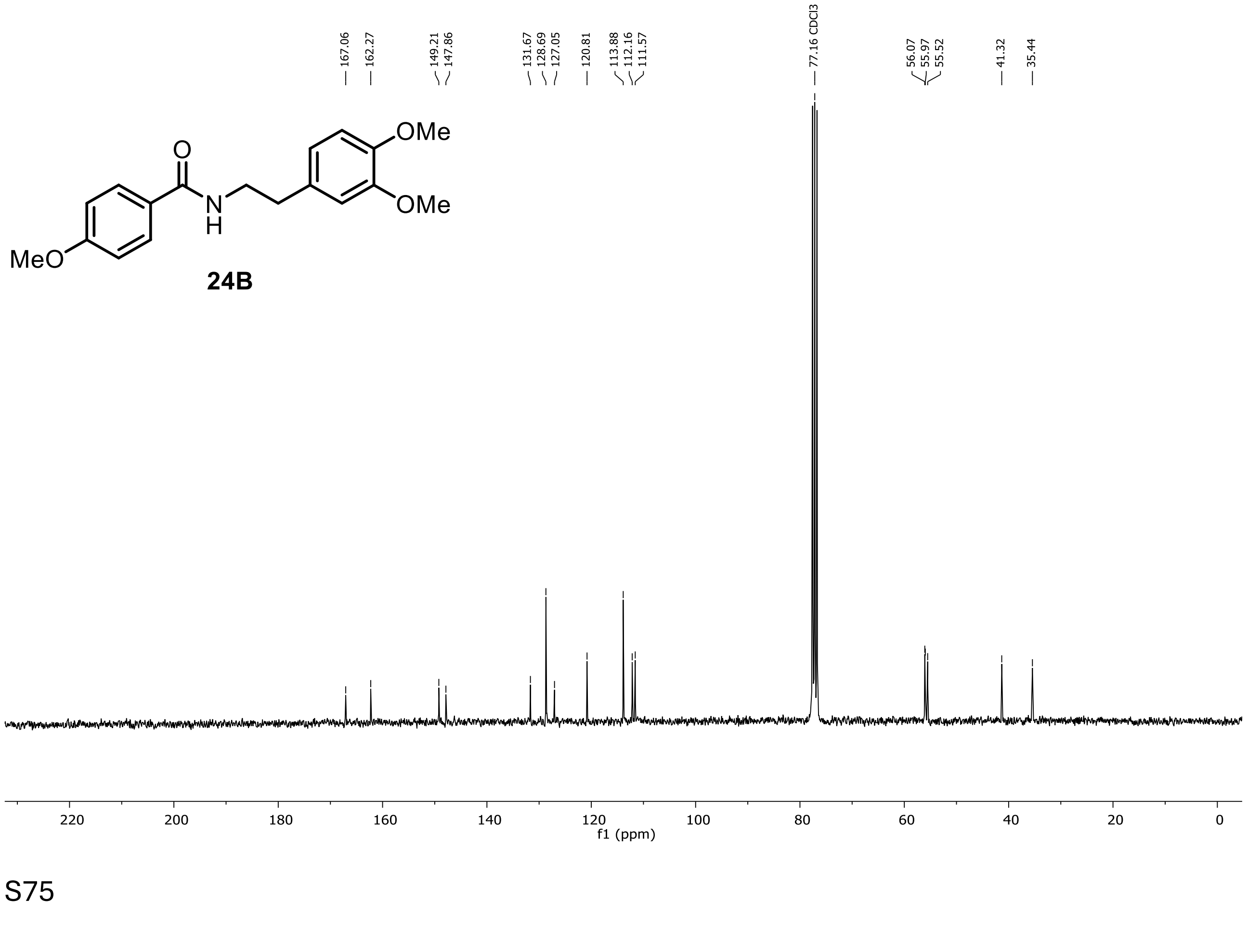

^{1}H NMR (300 MHz, Chloroform-d)

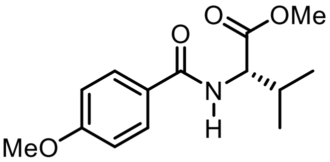

25B

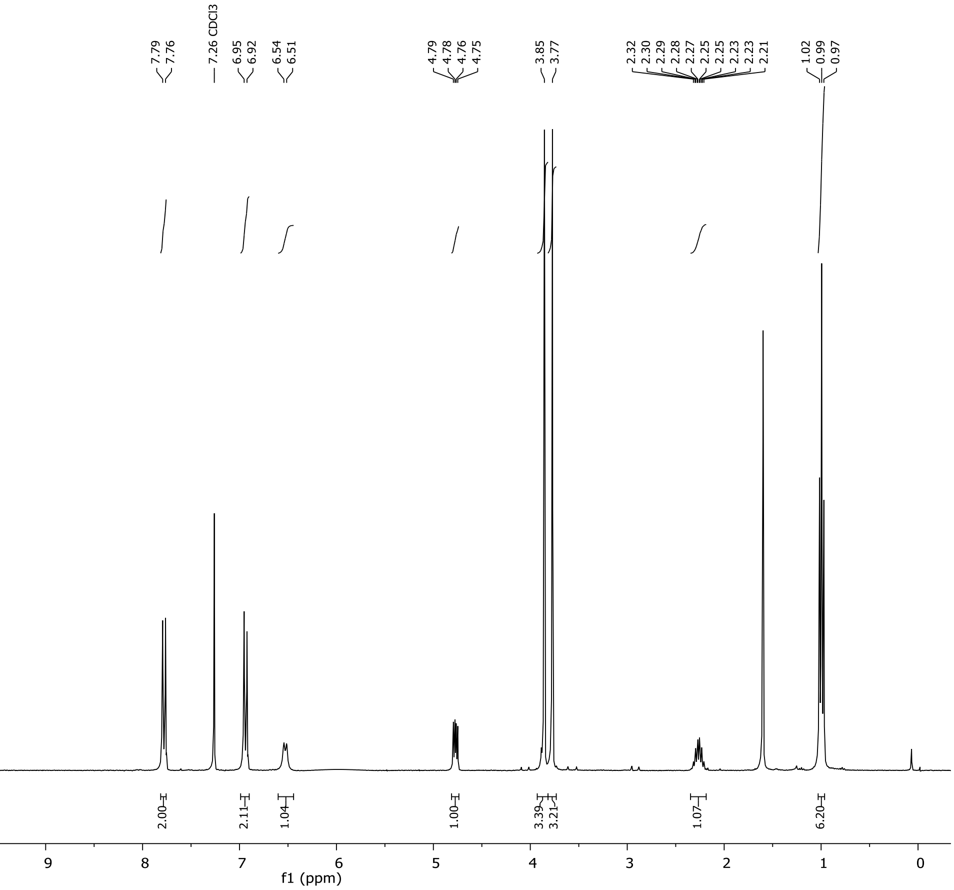

^{13}C NMR (75 MHz, Chloroform-d)

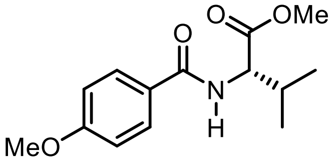

25B

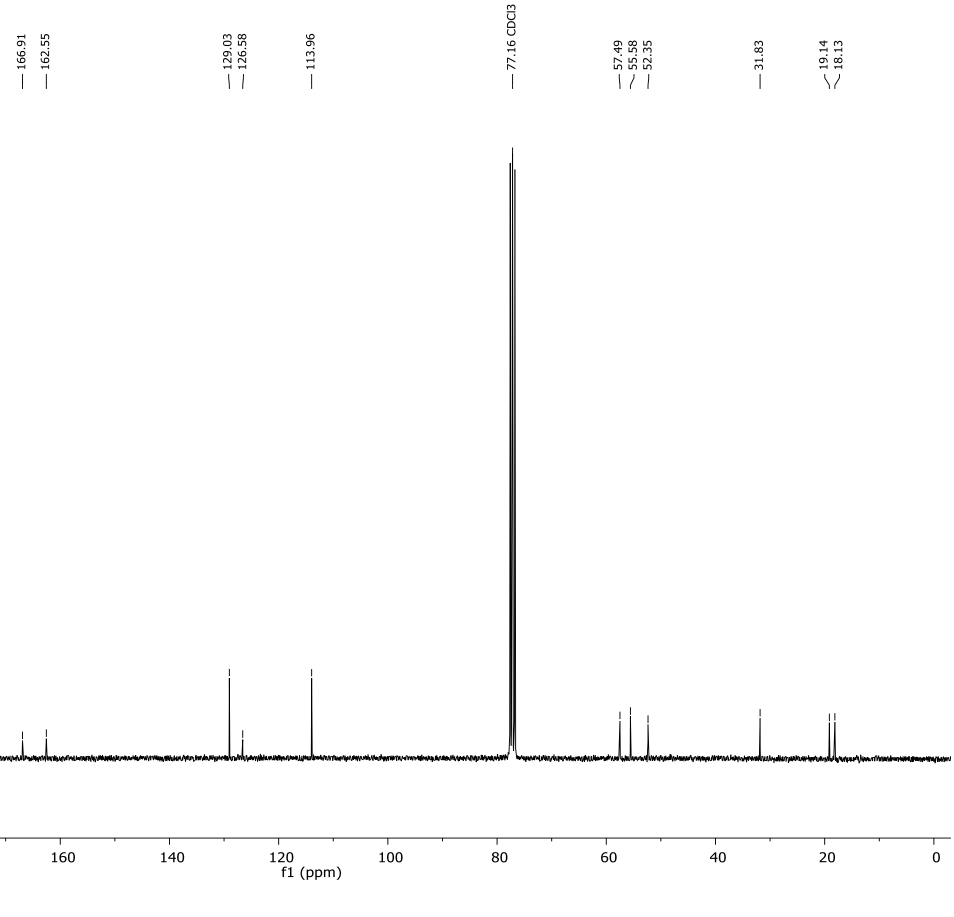

Supplement: Supplementary file 1 [file ol5c03618_si_001.pdf]
